# Tpz1-Ccq1 and Tpz1-Poz1 Interactions within Fission Yeast Shelterin Modulate Ccq1 Thr93 Phosphorylation and Telomerase Recruitment

**DOI:** 10.1371/journal.pgen.1004708

**Published:** 2014-10-16

**Authors:** Jennifer L. Harland, Ya-Ting Chang, Bettina A. Moser, Toru M. Nakamura

**Affiliations:** Department of Biochemistry and Molecular Genetics, College of Medicine, University of Illinois at Chicago, Chicago, Illinois, United States of America; National Cancer Institute, United States of America

## Abstract

In both fission yeast and humans, the shelterin complex plays central roles in regulation of telomerase recruitment, protection of telomeres against DNA damage response factors, and formation of heterochromatin at telomeres. While shelterin is essential for limiting activation of the DNA damage checkpoint kinases ATR and ATM at telomeres, these kinases are required for stable maintenance of telomeres. In fission yeast, Rad3^ATR^ and Tel1^ATM^ kinases are redundantly required for telomerase recruitment, since Rad3^ATR^/Tel1^ATM^-dependent phosphorylation of the shelterin subunit Ccq1 at Thr93 promotes interaction between Ccq1 and the telomerase subunit Est1. However, it remained unclear how protein-protein interactions within the shelterin complex (consisting of Taz1, Rap1, Poz1, Tpz1, Pot1 and Ccq1) contribute to the regulation of Ccq1 Thr93 phosphorylation and telomerase recruitment. In this study, we identify domains and amino acid residues that are critical for mediating Tpz1-Ccq1 and Tpz1-Poz1 interaction within the fission yeast shelterin complex. Using separation of function Tpz1 mutants that maintain Tpz1-Pot1 interaction but specifically disrupt either Tpz1-Ccq1 or Tpz1-Poz1 interaction, we then establish that Tpz1-Ccq1 interaction promotes Ccq1 Thr93 phosphorylation, telomerase recruitment, checkpoint inhibition and telomeric heterochromatin formation. Furthermore, we demonstrate that Tpz1-Poz1 interaction promotes telomere association of Poz1, and loss of Poz1 from telomeres leads to increases in Ccq1 Thr93 phosphorylation and telomerase recruitment, and telomeric heterochromatin formation defect. In addition, our studies establish that Tpz1-Poz1 and Tpz1-Ccq1 interactions redundantly fulfill the essential telomere protection function of the shelterin complex, since simultaneous loss of both interactions caused immediate loss of cell viability for the majority of cells and generation of survivors with circular chromosomes. Based on these findings, we suggest that the negative regulatory function of Tpz1-Poz1 interaction works upstream of Rad3^ATR^ kinase, while Tpz1-Ccq1 interaction works downstream of Rad3^ATR^ kinase to facilitate Ccq1 Thr93 phosphorylation and telomerase recruitment.

## Author Summary

Proper maintenance of telomeres is essential for maintaining genomic stability, and genomic instability caused by dysfunctional telomeres could lead to accumulation of mutations that may drive tumor formation. Telomere dysfunction has also been linked to premature aging caused by depletion of stem cells. Therefore, it is important to understand how cells ensure proper maintenance of telomeres. Mammalian cells and fission yeast cells utilize an evolutionarily conserved multi-subunit telomere protection complex called shelterin to ensure protection against telomere fusions by DNA repair factors and cell cycle arrest by DNA damage checkpoint kinases. However, previous studies have not yet fully established how protein-protein interactions within the shelterin complex contribute to the regulation of DNA damage checkpoint signaling and telomerase recruitment. By utilizing separation of function mutations that specifically disrupt either Tpz1-Ccq1 or Tpz1-Poz1 interaction within the fission yeast shelterin, we establish that Tpz1-Ccq1 interaction is essential for phosphorylation of Ccq1 by the DNA damage checkpoint kinases Rad3^ATR^ and Tel1^ATM^ that is needed for telomerase recruitment to telomeres, while Tpz1-Poz1 interaction prevents Ccq1 phosphorylation by promoting Poz1 association with telomeres. These findings thus establish for the first time how protein-protein interactions within the shelterin complex modulate checkpoint kinase-dependent phosphorylation essential for telomerase recruitment.

## Introduction

Telomeres are protective nucleoprotein structures found at the natural ends of eukaryotic linear chromosomes [1]. In most eukaryotes, telomeric DNA consists of GT-rich repeat sequences, primarily double-stranded but terminating in a single-stranded 3′ overhang, known as G-tail [1]. Telomerase, a specialized reverse transcriptase, solves the “end-replication problem” by *de novo* addition of GT-rich repeats to chromosome ends [2], [3]. While its catalytic subunit TERT (Trt1 in fission yeast *Schizosaccharomyces pombe*, Est2 in budding yeast *Saccharomyces cerevisiae*, and hTERT in humans) and telomerase RNA (*TER1* in *S. pombe*, *TLC1* in *S. cerevisiae*, and hTR in humans) [4]–[7] are sufficient for generating telomerase activity that can be monitored *in vitro* [8], [9], additional regulatory subunits are necessary for telomere maintenance *in vivo*. For example, a regulatory subunit Est1 is required for telomere maintenance in both budding and fission yeasts, as it contributes to efficient recruitment and/or activation of telomerase in late S-phase [10]–[12].

In mammalian cells, the shelterin complex (consisting of TRF1, TRF2, RAP1, TIN2, TPP1 and POT1) ensures stable maintenance of telomeres [1]. While TRF1 and TRF2 specifically recognize and bind to the double-stranded telomeric repeats, POT1 specifically binds to the G-tail [1]. TIN2 and TPP1 are essential for assembly of the shelterin complex, since these two proteins, via TRF1-TIN2, TRF2-TIN2, TIN2-TPP1, and TPP-POT1 interactions, connect the double-stranded DNA (dsDNA) binding proteins TRF1/TRF2 to the single-stranded DNA (ssDNA) binding protein POT1 [13]. On the other hand, a fully assembled shelterin complex might occur only transiently during cell cycle, since TRF1, TRF2 and POT1 show distinct cell cycle-regulated telomere binding patterns [14], and distinct sub-complexes of shelterin components have been identified in cell extracts [15]. In addition, a careful quantitative western blot analysis indicated that protein expression levels of TPP1 and POT1 are significantly lower than TRF1, TRF2, RAP1 and TIN2, suggesting that a majority of shelterin subunits may be assembled only as the TRF1-TRF2-RAP1-TIN2 sub-complex [16].

Previous studies have found that TRF1, along with TIN2, TPP1 and POT1, function as negative regulators of telomerase-dependent telomere elongation [17]–[20], but TIN2, POT1 and TPP1 also play roles in promoting telomere extension by facilitating telomerase recruitment [21]–[25]. The shelterin complex is also important for preventing telomeres from being recognized as broken DNA ends, which can undergo chromosome rearrangements and fusions by various DNA repair proteins, and cause cell cycle arrest mediated by the DNA damage checkpoint kinases ATM and ATR [26]–[28]. Mutations in telomerase and shelterin subunits have been linked to genomic instability and human diseases, highlighting the importance of understanding how the shelterin complex regulates telomerase and DNA damage response factors [29], [30].

**Figure 1:**
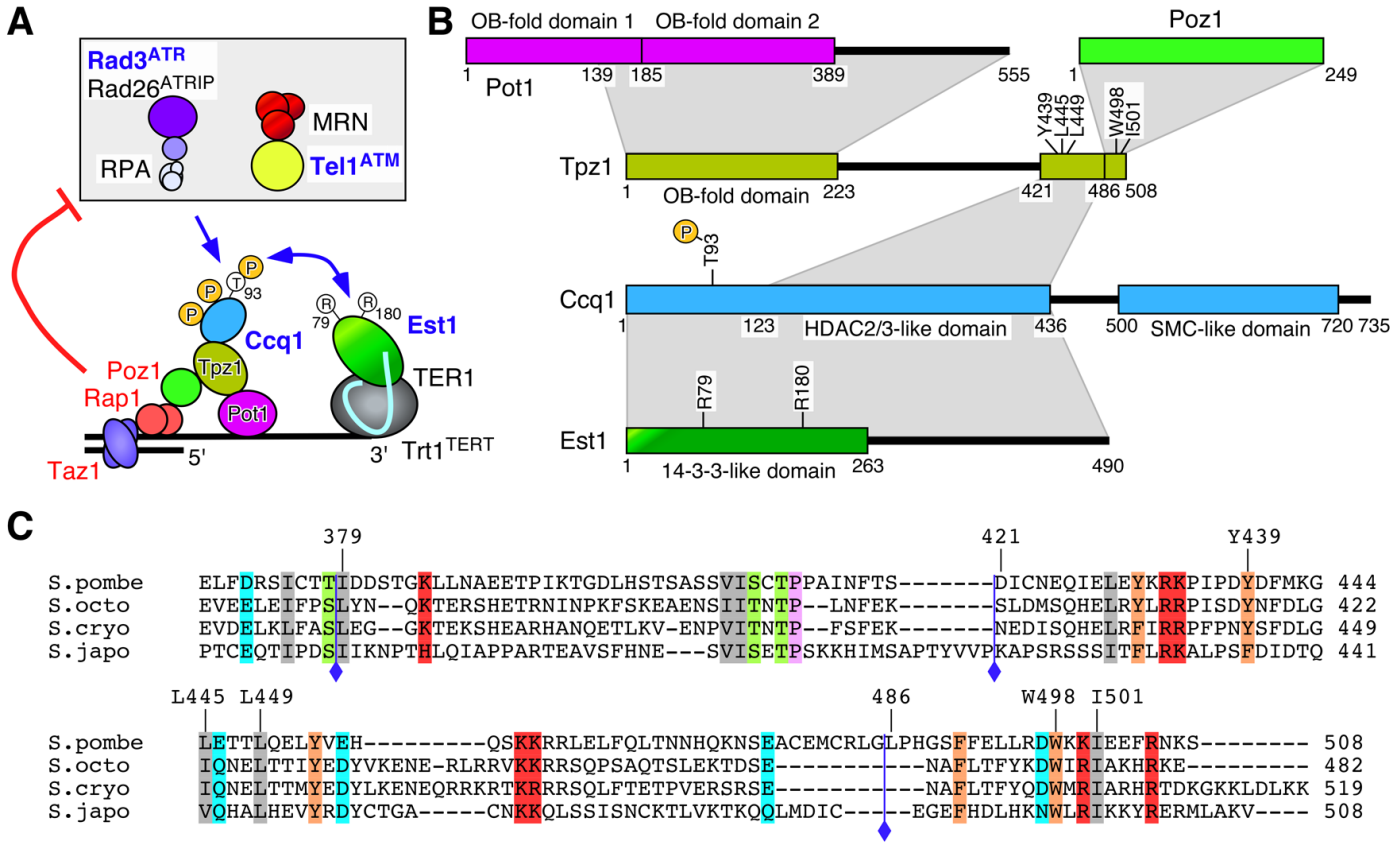
Summary of Tpz1-Ccq1 and Tpz1-Poz1 interaction domains. (**A**) A model of telomere maintenance regulation in fission yeast [12]. Taz1, Rap1 and Poz1 negatively regulate telomere extension by limiting accumulation of Rad3^ATR^ kinase, Ccq1 Thr93 phosphorylation and telomerase recruitment, while recognition of Ccq1 Thr93 phosphorylation by the 14-3-3-like domain of Est1 promotes telomerase recruitment [12]. (**B**) Summary of protein-protein interaction domains for shelterin and telomerase subunits determined by Y2H assays. The current study identified Tpz1 Y439, L445 and L449 as critical for Tpz1-Ccq1 interaction and Tpz1 W498 and I501 as critical for Tpz1-Poz1 interaction. A previous study has identified Ccq1 T93 and Est1 R79 and R180 as critical for Ccq1-Est1 interaction [12]. (**C**) Sequence alignment of Tpz1 region responsible for Tpz1-Ccq1 and Tpz1-Poz1 interaction in *S. pombe* and corresponding regions from three additional *Schizosaccharomyces* species (*S. octosporus*, *S. cryophilus* and *S. japonicus*). Blue diamonds indicate a previously defined Ccq1/Poz1 binding domain (379–508) [31], and newly defined Ccq1 binding (421–485) and Poz1 binding (486–508) domains. In addition, amino acid residues critical for Tpz1-Ccq1 (Y439, L445 and L449) and Tpz1-Poz1 (W498 and I501) interactions are indicated.

Fission yeast *Schizosaccharomyces pombe* serves as an attractive model for understanding how cells regulate telomere maintenance, since its shelterin complex (composed of Taz1, Rap1, Poz1, Tpz1, Pot1 and Ccq1) (Figure 1A) shares many conserved features with the mammalian shelterin complex [31], [32], and fission yeast cells are highly amenable to genetic and biochemical analyses. Pot1, the ortholog of mammalian POT1, binds directly to the G-tail and protects telomeres against chromosome fusions and Rad3^ATR^-dependent checkpoint activation [33], [34]. Due to loss of telomere protection, deletions of Pot1 or the Pot1-interacting protein Tpz1 (TPP1 ortholog) lead to immediate cell death for the majority of cells, while rare survivor cells carrying circularized chromosomes can be recovered [31], [33]. Poz1, proposed to be a functional analog of TIN2, forms a bridge between the ssDNA binding protein Pot1 and dsDNA binding protein Taz1 through its interactions with both Tpz1 and the Taz1-interacting protein Rap1 [31]. Much like in mammalian cells, different subunits of the fission yeast shelterin complex also show distinct cell cycle-regulated telomere association patterns [35], [36], suggesting that a commonly drawn fully connected shelterin complex (Figure 1A) might never be formed or formed only transiently during the cell cycle.

Taz1, Rap1, and Poz1 are all important for the negative regulation of telomerase-dependent telomere elongation, as deletion of any of these three proteins causes telomerase-dependent massive elongation of telomeres [31], [37]–[39]. We have previously shown that Taz1, Rap1 and Poz1 are necessary to properly coordinate leading and lagging strand synthesis and to limit accumulation of the DNA damage checkpoint kinase Rad3^ATR^ at telomeres [36]. Since Rad3^ATR^/Tel1^ATM^-dependent phosphorylation of Ccq1 at Thr93 is recognized by the 14-3-3-like domain of Est1 to promote Est1-Ccq1 interaction and telomerase recruitment [12], increased accumulation of Rad3^ATR^ in *taz1Δ*, *rap1Δ* and *poz1Δ* cells leads to increased Ccq1 Thr93 phosphorylation and telomerase recruitment [12], [36].

Ccq1 interacts not only with Tpz1 and Est1, but also with the Clr3 subunit of SHREC (Snf2/HDAC-Containing Repressor Complex), a mediator of heterochromatic gene silencing, to facilitate heterochromatin formation at telomeres [31], [40]. While yeast two-hybrid (Y2H) assays have detected Tpz1-Ccq1 and Ccq1-Clr3 interactions and co-immunoprecipitation (co-IP) assays have detected association of Pot1 with Ccq1 and association of Clr3 with Ccq1, co-IP assays failed to detect association of Pot1 with Clr3 [31], suggesting that shelterin and SHREC likely represent two distinct complexes with the shared subunit Ccq1. In addition to its roles as telomerase recruiter and promoter of heterochromatin formation, Ccq1 also acts to repress checkpoint activation and recombination at telomeres [31], [41]. However, previous studies have utilized *ccq1Δ* strains to examine its roles at telomeres, and thus did not address which of these previously described Ccq1 functions is dependent on the proper formation of Tpz1-Ccq1 interaction.

Poz1, besides functioning as a negative regulator of telomerase recruitment, is also required for heterochromatin formation at telomeres [32], and disruption of Poz1-Rap1 interaction leads to massively elongated telomeres and loss of heterochromatin [42]. However, it remained unclear if Poz1-Tpz1 interaction might play any functional role in this negative regulation of telomerase recruitment or heterochromatin formation at telomeres, as previous studies have utilized only *poz1Δ* strains [12], [31]. Simultaneous deletion of *ccq1* and *poz1* genes leads to severe telomere de-protection phenotypes reminiscent of *tpz1Δ* and *pot1Δ* cells, suggesting that Ccq1 and Poz1 play redundant roles in allowing Pot1-Tpz1 to protect telomeres against DNA repair and checkpoint proteins [31]. However, it had not been established if Tpz1-Poz1 and Tpz1-Ccq1 interactions are necessary for Poz1 and Ccq1 to protect telomeres, or the presence of these proteins per se are necessary for telomere capping.

Therefore, we have decided to map the domain(s) of Tpz1 that are responsible for dictating Tpz1-Poz1 and Tpz1-Ccq1 interaction, and to generate a series of *tpz1* mutations that are specifically defective in Tpz1-Poz1 and/or Tpz1-Ccq1 interaction while retaining Tpz1-Pot1 interaction. Using these separation-of-function mutants of Tpz1, we will establish that Tpz1-Ccq1 interaction is essential for Ccq1 Thr93 phosphorylation and telomerase recruitment, heterochromatin formation, and checkpoint suppression at telomeres, while Tpz1-Poz1 interaction is required for efficient localization of Poz1 at telomeres, heterochromatin formation, and inhibition of Ccq1 Thr93 phosphorylation and telomerase recruitment. Furthermore, we will establish that Tpz1-Poz1 and Tpz1-Ccq1 interactions are redundantly required for telomere protection. These conclusions are in conflict with a recent study that concluded that Tpz1-Ccq1 interaction is not necessary for telomerase recruitment and Tpz1-Poz1 interaction does not contribute to Poz1 recruitment to telomeres [43]. Therefore, we will also discuss contrasting observations between our current study and the previous findings.

## Results

### Identification of Tpz1-Ccq1 and Tpz1-Poz1 interaction domains within Tpz1

**Figure 2:**
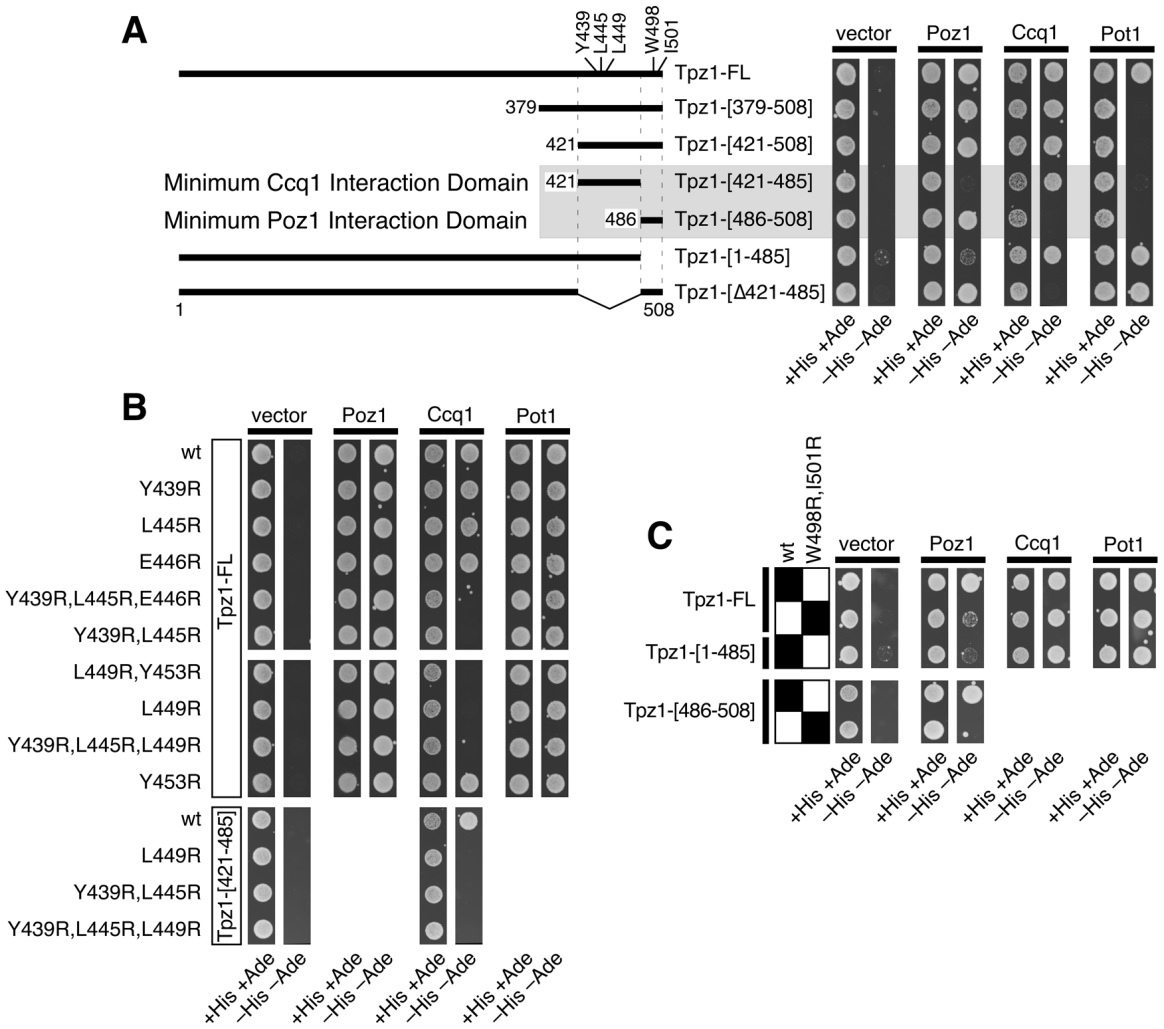
Determination of domains and amino acid residues critical for mediating Tpz1-Ccq1 and Tpz1-Poz1 interaction in Y2H assays. (**A**) Identification of minimum Tpz1-Ccq1 and Tpz1-Poz1 interaction domains by Y2H assay. A positive interaction is identified by growth on −His −Ade plates. (**B**) Identification of mutations that specifically disrupt Tpz1-Ccq1 interaction by Y2H assay. Indicated mutations were tested in full length Tpz1 (Tpz1-FL) or the minimum Ccq1 interaction domain construct of Tpz1 (Tpz1-[421–485]). (**C**) Identification of mutations that specifically disrupt Tpz1-Poz1 interaction by Y2H assay. The effect of W498R,I501R mutation was tested in full length Tpz1 (Tpz1-FL) or the minimum Poz1 interaction domain construct of Tpz1 (Tpz1-[486–508]).

A previous study that discovered the fission yeast shelterin complex also identified amino acids 379–508 of Tpz1 as the domain that mediates both Tpz1-Ccq1 and Tpz1-Poz1 interactions by Y2H assay [31] (Figure 1). In an attempt to identify smaller regions sufficient for mediating either Tpz1-Ccq1 or Tpz1-Poz1 interaction, we further truncated this fragment and found that amino acid residues 421–485 are sufficient for Tpz1-Ccq1 interaction while amino acid residues 486–508 are sufficient for Tpz1-Poz1 interaction by Y2H assay (Figure 2A).

Analysis of Tpz1 sequences from four *Schizosaccharomyces* species [44] identified highly conserved amino acid residues within the minimum Tpz1-Ccq1 and Tpz1-Poz1 interaction domains (Figures 1C and S1A). Therefore, we decided to mutate them to determine if these conserved residues play any role in promoting Tpz1-Ccq1 or Tpz1-Poz1 interaction. For charged residues (Lys, Arg, Asp and Glu), we introduced either a charge swap (Lys/Arg to Glu) or Alanine mutation (Lys/Arg/Asp/Glu to Ala), but failed to find any mutation that disrupt Tpz1-Poz1 or Tpz1-Ccq1 Y2H interaction (Figure S1B). On the other hand, we were able to identify mutations that specifically disrupt either Tpz1-Ccq1 or Tpz1-Poz1 Y2H interaction by changing hydrophobic residues (Tyr/Trp/Leu/Ile) to Arginine (Figure 2B–C).

Within its Ccq1 interaction domain, simultaneous mutations of Tyr439 and Leu445 or a single mutation of Leu449 to Arg disrupted the Tpz1-Ccq1 Y2H interaction without affecting Tpz1-Poz1 and Tpz1-Pot1 interactions (Figure 2B). Within its Poz1 interaction domain, simultaneous mutations of Trp498 and Ile501 to Arg significantly weakened the Tpz1-Poz1 Y2H interaction for full-length Tpz1, and completely eliminated the interaction when introduced into the minimal Poz1-binding domain construct (residues 486–508) (Figure 2C). When mutated to Ala, *tpz1-W498A,I501A* only mildly affected Tpz1-Poz1 Y2H interaction, and *tpz1-Y439A,I445A* failed to disrupt Tpz1-Ccq1 Y2H interaction (Figure S2B). While *tpz1-L449A* mutation also caused disruption of Tpz1-Ccq1 Y2H interaction (Figure S2B), the disruption was more severe for *tpz1-L449R* than *tpz1-L449A* (Figure S2C). Since we were interested in generating mutants that fully disrupt Tpz1-Ccq1 or Tpz1-Poz1 interaction, we then decided to focus our efforts on characterizing hydrophobic to Arg mutants rather than Ala mutants, except for *tpz1-L449A*.

**Figure 3:**
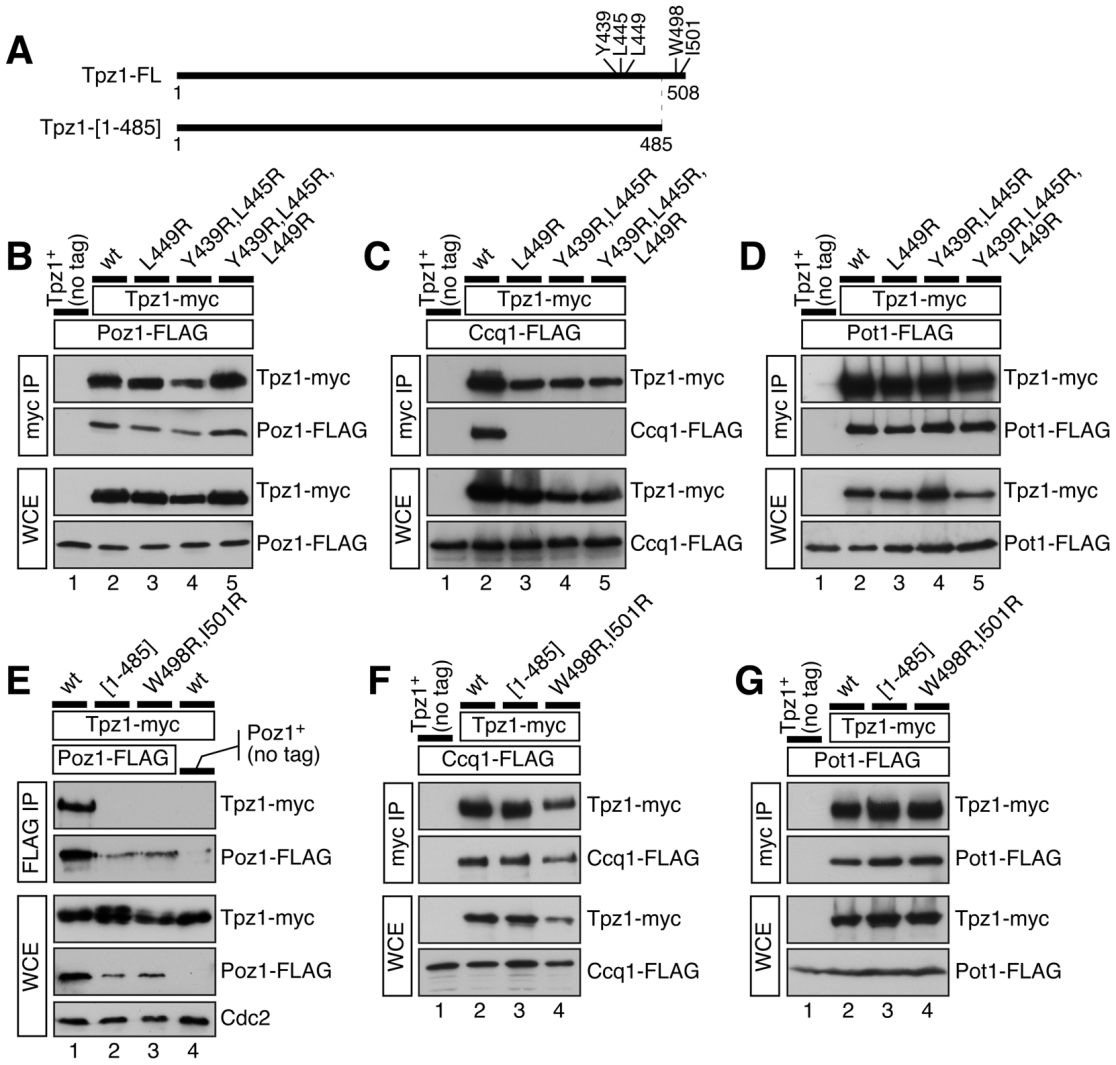
Co-IP experiments to examine how various Tpz1 mutations affect Tpz1-Poz1, Tpz1-Ccq1 and Tpz1-Pot1 interactions in fission yeast cells. (**A**) A schematic representation of full length (Tpz1-FL) or C-terminally truncated (Tpz1-[1–485]) Tpz1 constructs used in co-IP experiments. Locations of mutated amino acid residues are also indicated. (**B**–**D**) Examination of Tpz1-Poz1 (B), Tpz1-Ccq1 (C) and Tpz1-Pot1 (D) interactions by co-IP for indicated Tpz1-Ccq1 interaction disruption mutants. (**E**–**G**) Examination of Tpz1-Poz1 (E), Tpz1-Ccq1 (F) and Tpz1-Pot1 (G) interactions by co-IP for indicated Tpz1-Poz1 interaction disruption mutants. For strains carrying Poz1-FLAG, Cdc2 western blot was included to serve as loading control to show reduced expression of Poz1 in *tpz1-[1–485]* and *tpz1-W498R,I501R* backgrounds.

Based on our results from Y2H analysis, we integrated mutant *tpz1* alleles into their endogenous locus in fission yeast, and characterized their effects on Tpz1-Ccq1, Tpz1-Poz1 and Tpz1-Pot1 interactions by co-IP (Figures 3 and S3A). As predicted by Y2H assays, we found that Tpz1-L449R, Tpz1-Y439R,L445R and Tpz1-Y439R,L445R,L449R mutant proteins specifically lose Tpz1-Ccq1 interaction but retain Tpz1-Poz1 and Tpz1-Pot1 interactions in fission yeast cells (Figure 3B–D). In addition, co-IP analysis revealed that both L449R and L449A mutations both completely disrupt Tpz1-Ccq1 interaction even when lower salt (60 mM NaCl) than our standard salt (150 mM NaCl) lysis and wash conditions were used (Figure S3A). Furthermore, we found that both Tpz1-W498R,I501R and the C-terminally truncated Tpz1-[1–485] (Figure 3A) specifically disrupt Tpz1-Poz1 interaction without affecting Tpz1-Ccq1 and Tpz1-Pot1 interactions (Figure 3E–G). While all *tpz1* mutant alleles showed comparable expression levels of Tpz1 protein and did not affect protein levels of Pot1 or Ccq1 (Figure 3B–G), we found that Poz1 expression levels are reduced in cells expressing Tpz1-W498R,I501R or Tpz1-[1–485], suggesting that Poz1 stability partially depends on its interaction with Tpz1 (Figure 3E). Taken together, our results indicated that two distinct C-terminal regions of Tpz1 mediate its interaction with either Ccq1 or Poz1.

### Disruption of Tpz1-Ccq1 interaction resembles Ccq1 deletion

**Figure 4:**
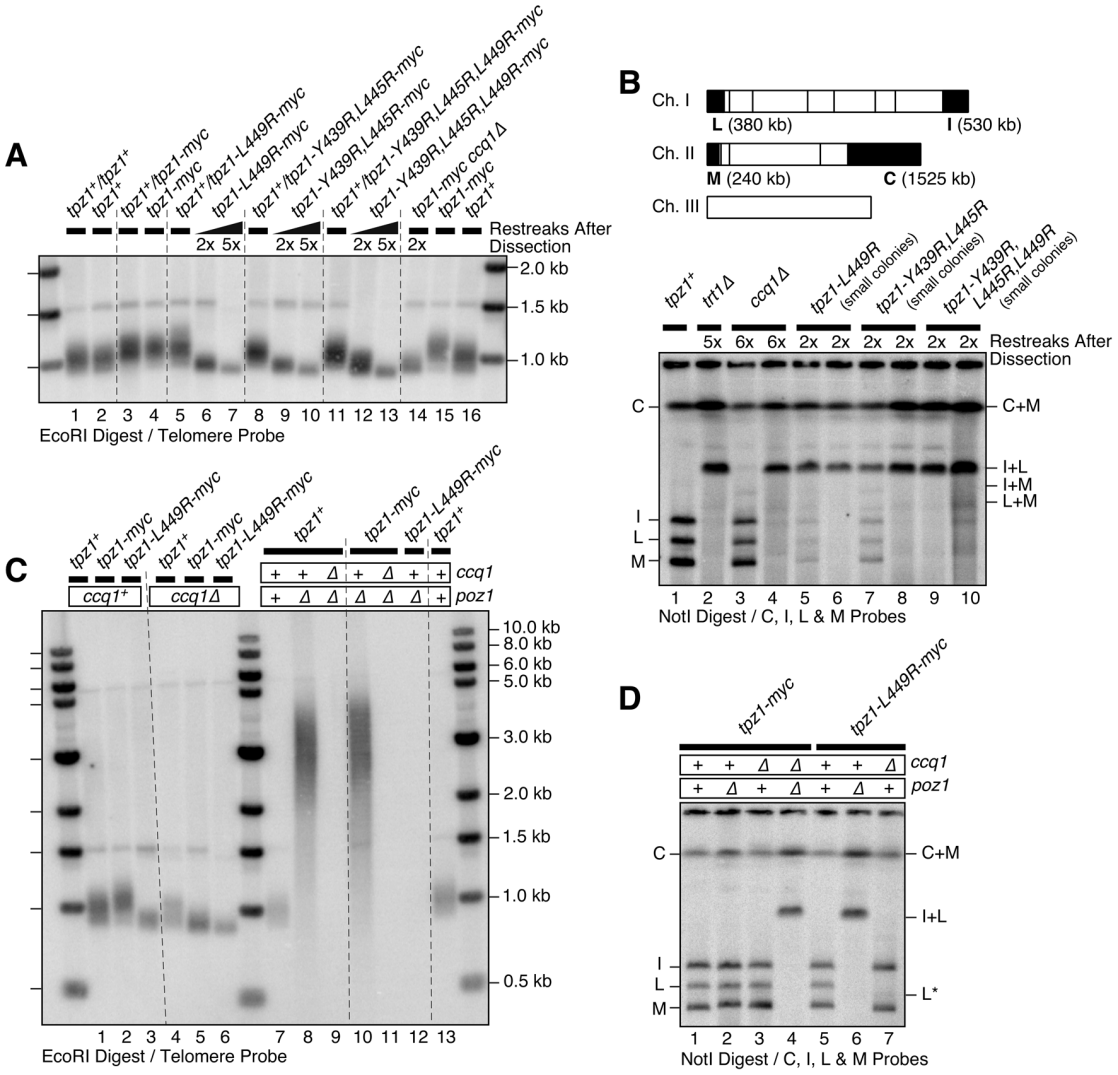
Effects of Tpz1-Ccq1 interaction disruption mutations on telomere maintenance. (**A**) Southern blot analysis of telomere length for indicated Tpz1-Ccq1 interaction disruption mutants. Haploid cells were generated by dissection of spores derived from heterozygous *tpz1^+^*/mutated *tpz1* diploid cells, and restreaked twice or 5 times on plates prior to preparation of genomic DNA. For each round of restreak, several faster growing colonies were combined and streaked for single colonies on YES plates. (**B**) Pulsed-field gel analysis of telomere fusions for early generation small colonies of Tpz1-Ccq1 interaction disruption mutants, which showed prominent I+L fusion band as well as much fainter bands for I+M, L+M, I, L and M bands. C and C+M bands could not be distinguished by size. A NotI restriction map of fission yeast chromosomes is shown on top with telomeric fragments from chromosomes I and II marked with black boxes (C, I, L and M). (**C**) Epistasis analysis for telomere maintenance phenotype of Tpz1-Ccq1 disruption mutants against *ccq1Δ* or *poz1Δ* by Southern blot. Cells were restreaked 5 times on plates prior to preparation of genomic DNA to achieve steady state telomere length, except for *tpz1-myc ccq1Δ poz1Δ* and *tpz1-L449R-myc poz1Δ* cells where DNA from survivors with circular chromosomes were made after restreaked twice on plates. (**D**) Epistasis analysis for telomere fusions by pulsed-field gel for indicated combination of tpz1 mutants, *ccq1Δ* and *poz1Δ*. For (C–D), samples were prepared from early generation cells after strains were generated by genetic cross of parental haploid strains and dissection of resulting double mutant spores.

Identification of Tpz1 mutants that specifically disrupt either Tpz1-Ccq1 or Tpz1-Poz1 interaction allowed us to investigate the functional significance of these interactions for fission yeast telomere maintenance. For all four Tpz1-Ccq1 interaction mutants tested (*tpz1-L449R*, *tpz1-L449A*, *tpz1-Y439R,L445R* and *tpz1-Y439R,L445R,L449R*), Southern blot analysis indicated that newly generated haploid *tpz1* mutant cells (derived from heterozygous diploid cells) show progressive telomere shortening when restreaked on agar plates, very much like *ccq1Δ* cells (Figure 4A lanes 6, 7, 9, 10, 12 and 13, and Figure S3B lanes 2–9) [31], [41]. However, colony sizes of *tpz1* mutant cells on plates were highly variable, and when smaller colonies were selectively picked to monitor telomere structures, even early generation colonies (restreaked twice) showed very strong signals for a band corresponding to intra-chromosome fusion (I+L), and faint signals for bands corresponding to inter-chromosome fusions (I+M and L+M) on a pulsed-field gel (Figures 4B and S3C). Conversely, when faster growing colonies were chosen for successive restreaks on plates, they maintained short telomeres even after restreaked 11 times (estimated to be 220–275 cell divisions, assuming 20–25 cell divisions per restreak on agar plates) (Figure S4), suggesting that those cells have adapted to the loss of Tpz1-Ccq1 interaction and managed to stably maintain short telomeres. Furthermore, *tpz1-L449R* or *tpz1-L449A* cells derived from larger colonies also showed no telomere fusions when analyzed on a pulsed field gel (Figure 4D lane 5, and Figure S3E lanes 2, 3 and 7). Taken together, we thus concluded that the loss of Tpz1-Ccq1 interaction causes heterogeneous phenotypes with either immediate loss of cell viability due to elevated telomere fusions or frequent emergence of cells that manage to maintain short telomeres for many generations.

In liquid culture, *tpz1-L449R* and *ccq1Δ* cells showed similar changes in cell growth rate. The slowest cell growth occurred on days 6–8 after haploid colonies derived from heterozygous diploid cells were first inoculated and subsequently serially diluted every 24 hours (Figure S5). Progressive telomere shortening of *tpz1-L449R* and *ccq1Δ* cells during days 1–7 correlated with progressive loss of growth, but both cultures eventually generated survivors that recover in growth rate and carry highly elongated and rearranged telomeres (Figure S5C–D). Intensities of telomere signals for *tpz1-L449R* and *ccq1Δ* survivor cells were much more intense than for *trt1Δ* survivor cells (Figure S5B–D), suggesting that elimination of Tpz1-Ccq1 interaction or Ccq1 protein may allow for more efficient telomere elongation by telomere-telomere recombination in survivor cells [41], [45]. The appearance of survivor cells with highly elongated telomeres in liquid culture is reminiscent of the situation in budding yeast, where rare but faster growing budding yeast Type II recombination survivors with long telomeres predominate in liquid culture, and more common but slower growing Type I survivors with short telomeres predominate when selected on agar plates [46], [47].

Double mutant *tpz1-L449R ccq1Δ* cells grew comparably to *tpz1-L449R* and *ccq1Δ* single mutant cells, and Southern blot analysis revealed that *tpz1-L449R ccq1Δ* double mutant cells exhibit a similar extent of telomere shortening as *tpz1-L449R* and *ccq1Δ* single mutant cells (Figure 4C lanes 3, 5 and 6). By contrast, the majority of *tpz1-L449R poz1Δ* and *tpz1-L449A poz1Δ* double mutant cells died immediately after they were generated by dissection of spores derived from heterozygous diploid cells, and rare survivor cells had lost their telomeres (Figures 4C and S3D) and carried circular chromosomes (Figures 4D and S3E), much like *ccq1Δ poz1Δ* double mutant cells. These data supported the notion that disruption of Tpz1-Ccq1 interaction mainly affects the Ccq1-dependent pathway of telomere maintenance.

Much like *ccq1Δ* cells [41], Tpz1-Ccq1 interaction disruption mutants immediately activated the G_2_ DNA damage checkpoint, based on the appearance of highly elongated cells and a slow mobility band corresponding to hyper-phosphorylated Chk1 on SDS PAGE (Figure S6). In addition, *tpz1-L449R* and *tpz1-Y439R,L445R* cells, much like *ccq1Δ* cells, failed to repress the *his3^+^* gene inserted adjacent to telomere repeats, suggesting that Tpz1-Ccq1 interaction is essential for heterochromatin formation at telomere/sub-telomere regions (Figure S7A) [48]. Thus, disruption of Tpz1-Ccq1 interaction recapitulated all phenotypes of *ccq1Δ* cells we have examined, highlighting the importance of this interaction not only for telomerase regulation and telomere protection [12], [31], [41], [49] but also for the SHREC-dependent role of Ccq1 in heterochromatin formation at sub-telomeres [40].

### Tpz1-Ccq1 interaction is required for Ccq1 Thr93 phosphorylation and telomerase recruitment

**Figure 5:**
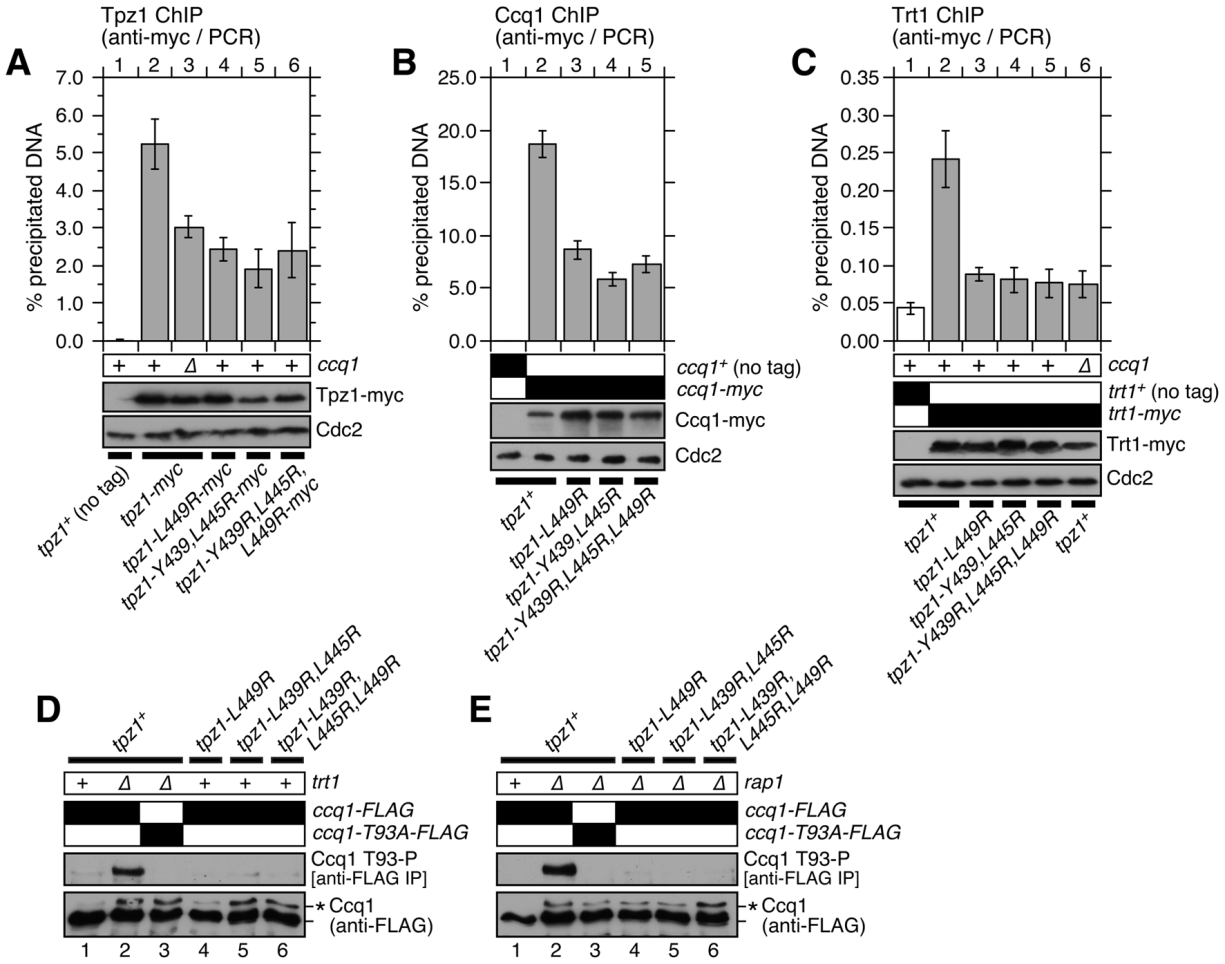
Telomere association of Tpz1, Ccq1 and Trt1^TERT^ in Tpz1-Ccq1 interaction mutant cells. Effects of disrupting Tpz1-Ccq1 interaction on telomere association for (**A**) Tpz1, (**B**) Ccq1 and (**C**) Trt1^TERT^ were monitored by ChIP assays. Error bars represent standard error of the mean from three to nine independent experiments. Statistical analysis of ChIP data by 2-tailed Student's t-test is shown in Table S5. Southern blot analyses of telomere lengths for strains used in ChIP assays are shown in Figure S8A–C. Expression levels of myc-tagged proteins were monitored by anti-myc western blot analysis of whole cell extracts. Anti-Cdc2 blots served as loading control. (**D**–**E**) Ccq1 Thr93 phosphorylation, detected by anti-phospho(S/T)Q (Phospho-(Ser/Thr) ATM/ATR substrate antibody in both *rap1^+^* (D) or *rap1Δ* (E) backgrounds, is abolished in Tpz1-Ccq1 interaction disruption mutants. For anti-FLAG blots, asterisk (*) indicates hyper-phosphorylated form of Ccq1.

Next, we performed chromatin immunoprecipitation (ChIP) assays to understand how disruption of Tpz1-Ccq1 interaction affects the association of Tpz1, Ccq1 and Trt1^TERT^ with telomeres. We utilized early generation mutant strains, and the presence of telomeres was confirmed by Southern blot analysis (Figure S8A–C). The amount of Tpz1 bound to telomeres was reduced in *tpz1* mutant cells which disrupt Tpz1-Ccq1 interaction (*tpz1-L449R*, *tpz1-Y439R,L445R* and *tpz1-Y439R,L445R,L449R*), much like in *ccq1Δ* cells (Figure 5A). Likewise, Ccq1 association with telomeres was reduced in these mutants, but retained substantial binding (Figure 5B). Furthermore, we found that *tpz1-L449A* and *tpz1-L449R* cause comparable reduction in Ccq1 binding at telomeres (Figure S9A). Thus, even though Tpz1 is the only shelterin subunit known to directly interact with Ccq1 [31], we concluded that Ccq1 could still be recruited to telomeres in the absence of detectable Tpz1-Ccq1 interaction, strongly implicating the existence of additional mechanism(s) that allow recruitment of Ccq1 to telomeres. For both Tpz1 and Ccq1, the reduction in telomere binding we observed could be a consequence of having fewer telomeric repeats. On the other hand, previous studies have found that *ccq1-T93A* cells, despite having short telomeres, do not show reduced Ccq1 association with telomeres [12], [50], suggesting that Tpz1-Ccq1 does indeed contribute to the efficient accumulation of Ccq1 at telomeres.

Ccq1 is required for recruitment of telomerase to telomeres [41], [49]. Thus, we next examined the association of telomerase with telomeres by performing ChIP assays for the catalytic subunit of telomerase Trt1^TERT^. Despite the fact that substantial amounts of Ccq1 remained bound to telomeres, we found that Trt1^TERT^ binding to telomeres is nearly eliminated in all Tpz1-Ccq1 interaction disruption mutants, much like in *ccq1Δ* cells (Figures 5C and S9B). Furthermore, we found that Tpz1 fails to co-IP with telomerase RNA *TER1* in the absence of Tpz1-Ccq1 interaction (Figure S8D).

Because Rad3^ATR^/Tel1^ATM^-dependent Ccq1 Thr93 phosphorylation is required for recruitment of telomerase to telomeres [12], [50], we hypothesized that the loss of telomerase recruitment may be explained by loss of Ccq1 Thr93 phosphorylation. We examined Ccq1 phosphorylation in both *rap1^+^* and *rap1Δ* backgrounds, since elimination of Rap1 strongly induces Rad3^ATR^/Tel1^ATM^-dependent hyper-phosphorylation of Ccq1 at multiple sites including Thr93 [12], allowing us to more robustly determine the effect of disrupting Tpz1-Ccq1 interaction on Ccq1 phosphorylation.

Based on the appearance of a phosphatase sensitive slow mobility band on SDS PAGE, we found that disruption of Tpz1-Ccq1 interaction alone, much like *trt1Δ*, is sufficient to induce hyper-phosphorylation of Ccq1, due to telomere shortening [12] (Figure 5D bottom panel). Moreover, Ccq1 was still hyper-phosphorylated when Tpz1-Ccq1 interaction was disrupted in *rap1Δ* cells (Figure 5E bottom panel). By contrast, disruption of Tpz1-Ccq1 interaction completely eliminated Ccq1 Thr93 phosphorylation in both *rap1^+^* and *rap1Δ* backgrounds (Figure 5D–E top panels). Taken together, we thus concluded that Tpz1-Ccq1 interaction plays an essential role in telomerase recruitment by facilitating Rad3^ATR^/Tel1^ATM^-dependent phosphorylation of Ccq1 Thr93. Furthermore, our data indicated that Ccq1 Thr93 phosphorylation is differentially regulated from phosphorylation of other Ccq1 sites and much more dependent on Tpz1-Ccq1 interaction.

### Disruption of Tpz1-Poz1 interaction resembles Poz1 deletion

**Figure 6:**
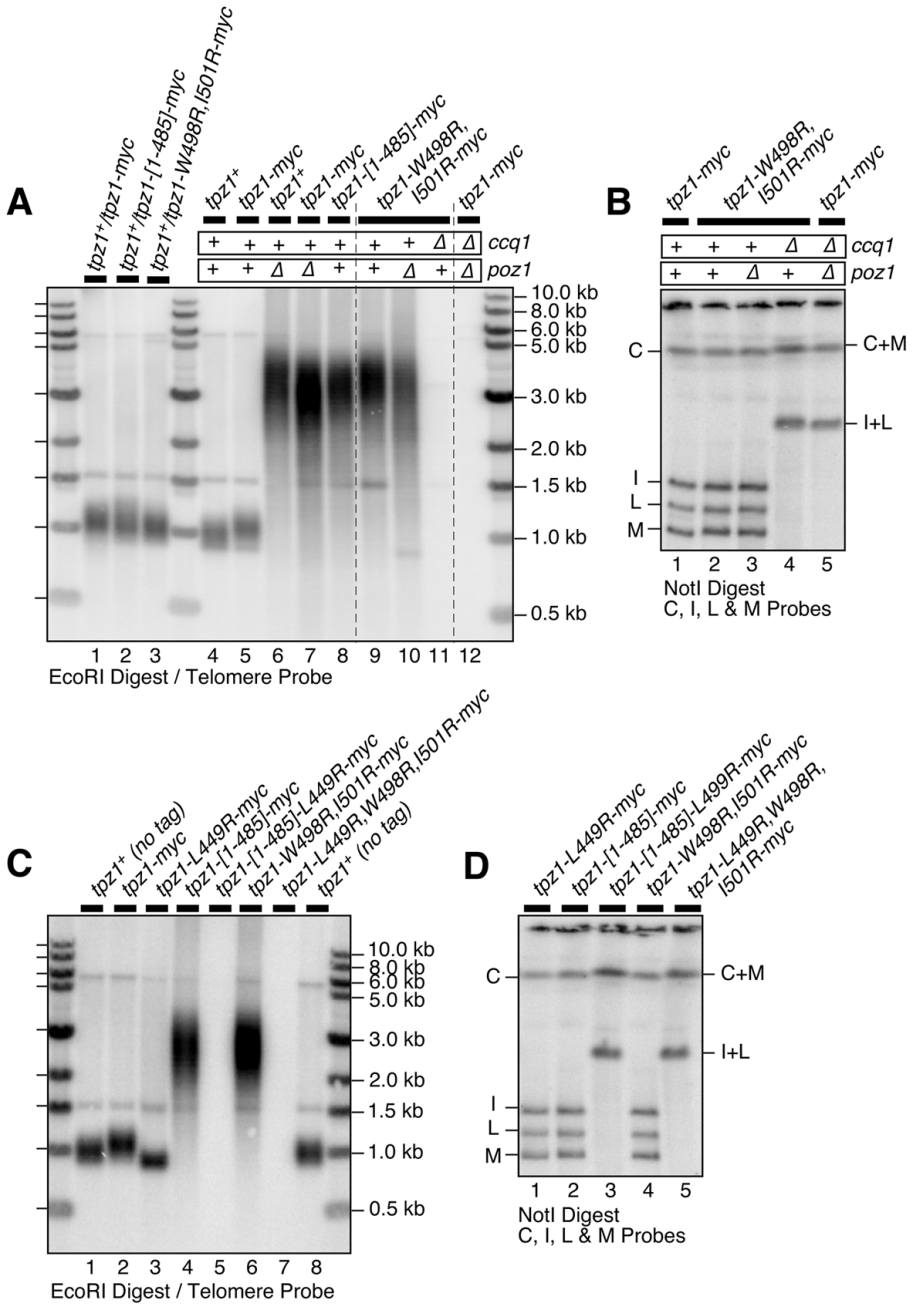
Effects of Tpz1-Poz1 interaction disruption mutations on telomere maintenance. (**A**) Southern blot analysis and (**B**) pulsed-field gel analysis for indicated Tpz1-Poz1 interaction disruption mutants. Haploid cells were generated by dissection of spores derived from heterozygous *tpz1^+^*/mutated *tpz1* diploid cells, and restreaked 5 times on plates prior to preparation of genomic DNA to achieve steady state telomere length, except for *tpz1-W499R,I501R poz1Δ* and *ccq1Δ poz1Δ* cells where DNA from survivors with circular chromosomes were made after restreaked twice on plates. For each round of restreak, several faster growing colonies were combined and streaked for single colonies on YES plates. (**C**) Southern blot analysis and (**D**) pulsed-field gel analysis for mutants that disrupted either Tpz1-Ccq1 or Tpz1-Poz1 interaction, or both Tpz1-Ccq1 and Tpz1-Poz1 interactions. DNA samples were prepared after restreaked twice on plates. Simultaneous loss of Tpz1-Ccq1 and Tpz1-Poz1 interactions resulted in complete loss of telomeres and circularization of chromosomes, much like in *ccq1Δ poz1Δ*, *tpz1-L449R poz1Δ* and *tpz1-W498R,I501R ccq1Δ* cells.

When various truncation mutants of Tpz1, which all expressed well in fission yeast based on western blot analysis (Figure S10A–B), were tested for their effects on telomere maintenance, we found that deletion of the internal Tpz1-Ccq1 interaction domain alone (*tpz1-[Δ421–485]*) or deletion of both Tpz1-Ccq1 and Tpz1-Poz1 interaction domains (*tpz1-[1–420]*) result in immediate telomere loss and chromosome circularization (Figure S10C–D). By contrast, deletion of the Tpz1-Poz1 interaction domain alone (*tpz1-[1–485]*) allowed cells to maintain highly elongated telomeres, much like in *poz1Δ* cells (Figure 6A lanes 7 and 8, and Figure S10C lane 6).

Tpz1 point mutations that disrupted Tpz1-Poz1 interaction (*tpz1-W498R,I501R*) (Figure 3E) likewise caused telomere elongation comparable to *poz1Δ*, and telomeres did not show any further elongation in *tpz1-W498R,I501R poz1Δ* cells (Figure 6A lanes 7, 9 and 10). Furthermore, *tpz1-W498R,I501R ccq1Δ* cells immediately lost telomeres, as soon as they were germinated from spores derived from heterozygous diploid (*tpz1^+^/tpz1-W498R,I501R ccq1^+^/ccq1Δ*) cells, and survived by circularizing their chromosomes, very much like in *ccq1Δ poz1Δ* cells (Figure 6A lanes 11 and 12, and Figure 6B lanes 4 and 5). We also observed that cells carrying *tpz1* mutants that incorporate disruption mutations for both Tpz1-Ccq1 and Tpz1-Poz1 interactions (*tpz1-[1–485]-L449R* and *tpz1-L449R,W498R,I501R*) fail to protect telomeres against fusions, immediately lose viability for the majority of cells, and exclusively generate survivors with circular chromosomes (Figure 6C lanes 5 and 7, and Figure 6D lanes 3 and 5).

Taken together, we thus concluded that telomere length deregulation caused by disrupting Tpz1-Poz1 interaction specifically inactivates Poz1's ability to prevent uncontrolled telomere elongation. Furthermore, we concluded that Tpz1-Poz1 and Tpz1-Ccq1 interactions redundantly provide essential telomere protection functions of Tpz1 [31]. While it remains to be established why Ccq1 and Poz1 are redundantly required to prevent telomere fusions, we can rule out the possibility that they are redundantly required for assembly of the Tpz1-Pot1 complex, since Tpz1-Pot1 interaction detected by co-IP remain intact in *ccq1Δ poz1Δ* cells (Figure S11D). Furthermore, in a Pot1-dependent *in vitro* pull down assay for Tpz1 utilizing a magnetic-bead coupled telomeric G-tail oligo, wild-type Tpz1 could still be detected in *ccq1Δ poz1Δ* cells, and both Tpz1-[1–485]-L449R and Tpz1-L449R,W498R,I501R mutant proteins, which interact with neither Ccq1 nor Poz1, were also detected (Figure S11A–C).

Disruption of Tpz1-Poz1 interaction also allowed expression of the *his3^+^* gene inserted adjacent to telomere repeats (Figure S7B), much like *poz1Δ* cells [49], suggesting that heterochromatin formation at telomeres also requires Tpz1-Poz1 interaction. However, both *tpz1-W498,I501R* and *tpz1-[1–485]* cells grew slower than *poz1Δ* cells on selective media lacking histidine, suggesting that Poz1, even in the absence of Tpz1-Poz1 interaction, weakly contributes to the formation of heterochromatin at telomeres.

### Disruption of Tpz1-Poz1 interaction causes strong reduction in Poz1 binding to telomeres, and increases Ccq1 Thr93 phosphorylation and telomerase recruitment

In order to gain insight into how the disruption of Tpz1-Poz1 interaction affects the association of shelterin subunits and telomerase with telomeres, we next carried out ChIP assays for Tpz1, Ccq1, Poz1 and Trt1^TERT^. It was necessary to utilize dot blot-based ChIP assays, rather than quantitative real-time PCR-based ChIP assays, since *tpz1-W498R,I501R* caused massive elongation of telomere repeats (Figures 6A and S12) and thus putting the sub-telomeric annealing sites for our PCR primers too far away from actual telomeric ends [12], [36].

By quantifying hybridization intensities of precipitated and input DNA to a telomeric repeat DNA probe, we first established % DNA that was precipitated by ChIP relative to input (raw % precipitated DNA) (Figure S13). Changes in raw % precipitated DNA values more closely reflect changes in density of a given protein on telomeric repeats, rather than changes in total amount of protein associated per chromosome end. Thus, it became necessary to correct raw % precipitated DNA values for telomere length to better represent changes in amount of protein bound per chromosome end, especially for cells carrying highly elongated telomeres.

To account for changes in telomere length, we first established “telomere length correction factors” for individual strains by measuring changes in telomere/rDNA hybridization intensity ratios compared to wild-type cells (Table S1) [36]. We then established “telomere length corrected” ChIP values by multiplying background subtracted % precipitated DNA values (raw % precipitated DNA from epitope tagged strain – no tag control % precipitated DNA) with the telomere length correction factors, and normalizing them to wild-type ChIP values (plotted as “relative ChIP signal”) [36]. Although not perfect, this adjustment for variations in telomere length allowed us to better estimate changes in amount of protein localized per chromosome end.

**Figure 7:**
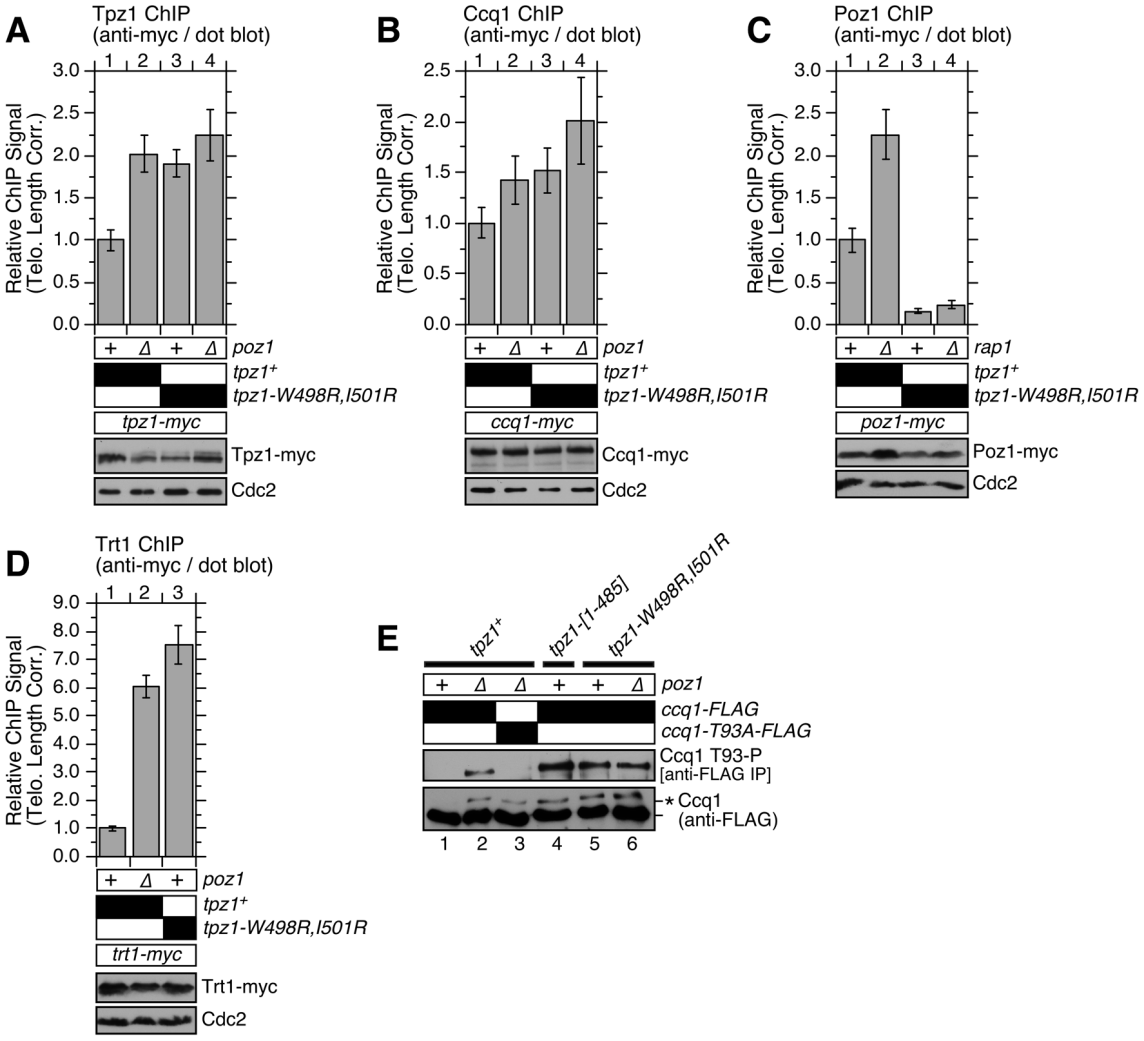
Telomere association of Tpz1, Ccq1, Poz1 and Trt1^TERT^ in Tpz1-Poz1 interaction mutant cells. Effects of disrupting Tpz1-Poz1 interaction on telomere association for (**A**) Tpz1, (**B**) Ccq1, (**C**) Poz1 and (**D**) Trt1^TERT^ were monitored by dot-blot ChIP assays and corrected for changes in telomere length [36]. Error bars represent standard error of the mean from three to eight independent experiments. Statistical analysis of ChIP data by 2-tailed Student's t-test is shown in Table S5. Southern blot analysis of telomere length for strains used in ChIP assays is shown in Figure S12, and raw data for ChIP assays are shown in Figure S13. Expression levels of myc-tagged proteins were monitored by anti-myc western blot analysis of whole cell extracts. Anti-Cdc2 blots served as loading control. (**E**) Ccq1 Thr93 phosphorylation, detected by anti-phospho(S/T)Q (Phospho-(Ser/Thr) ATM/ATR substrate antibody, is enhanced in mutant cells lacking Tpz1-Poz1 interaction. For anti-FLAG blots, asterisk (*) indicates hyper-phosphorylated form of Ccq1.

Analysis of ChIP data revealed that *tpz1-W498R,I501R*, *poz1Δ* and *tpz1-W498R,I501R poz1Δ* cells show comparable increases in amount of Tpz1 and Ccq1 per chromosome end over wild-type cells when corrected for telomere elongation in these mutant cells (Figure 7A–B). Since single and double mutants for *tpz1-W498R,I501R* and *poz1Δ* showed comparable changes in Tpz1 and Ccq1 association with telomeres, these ChIP data further confirmed that the loss of Tpz1-Poz1 interaction solely disrupts Poz1 function at telomeres.

Further analysis of Poz1 ChIP data indicated that Tpz1-Poz1 interaction is crucial for efficient accumulation of Poz1 at telomeres, as *tpz1-W498R,I501R* or *tpz1-W498R,I501R rap1Δ* cells showed a substantial reduction in Poz1 association with telomeres compared to wild-type cells (Figure 7C). By contrast, *rap1Δ* cells showed an increase in Poz1 association (Figure 7C) [36]. We have previously shown that *rap1Δ* also causes a comparable increase in telomere association for Tpz1 and Ccq1 [36]. Taken together, we concluded that Poz1 association with telomeres is primarily facilitated by Tpz1-Poz1 interaction, and that Poz1-Rap1 interaction does not play a significant role in association of Poz1 with telomeres.

On the other hand, it should be noted that Poz1 association, although significantly decreased, is not completely eliminated even in *tpz1-W498R,I501R rap1Δ* cells (Figures 7C and S13C). As noted earlier, we found that the presence of Poz1 protein appears to contribute weakly to the transcriptional repression of the *his3^+^* marker in *tpz1-W498R,I501R* cells (Figure S7B). Therefore, residual Rap1- and Tpz1-independent association of Poz1 with telomeres may also be functionally important. Alternatively, since Tpz1-W498R,I501R protein still showed residual interaction with Poz1 in Y2H assay (Figure 2C), it might also retain a residual weak interaction *in vivo* (not detected by co-IP) that is responsible for its residual localization to telomeres.

Since introduction of *tpz1-W498R,I501R* or *tpz1-[1–485]* caused telomere extensions comparable to *poz1Δ* (Figure 6A), we expected that loss of Tpz1-Poz1 interaction would cause increases in both telomerase association with telomeres and Ccq1 Thr93 phosphorylation, as previously established for *poz1Δ* cells [12], [36]. Indeed, ChIP assays for the telomerase catalytic subunit Trt1^TERT^ revealed that *tpz1-W498R,I501R* causes a comparable increase in Trt1^TERT^ binding to telomeres as *poz1Δ* cells (Figures 7D and S13D). In addition, we found that both *tpz1-W498R,I501R* and *tpz1-[1–485]* mutations cause hyper-phosphorylation at Thr93 and other sites of Ccq1 (Figure 7E). Thus, we concluded that Tpz1-Poz1 interaction-dependent recruitment of Poz1 is essential for enforcing a negative regulation on Ccq1 Thr93 phosphorylation-dependent recruitment of telomerase.

## Discussion

### Tpz1-Ccq1 and Tpz1-Poz1 interactions modulate Ccq1 Thr93 phosphorylation and telomerase recruitment

**Figure 8:**
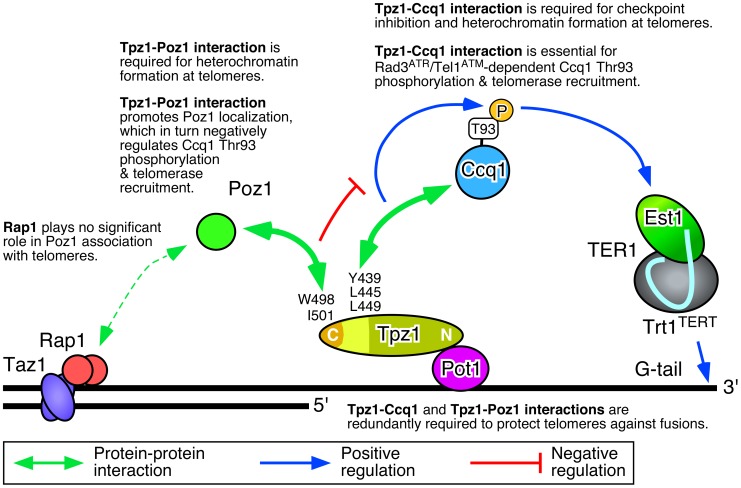
Summary of key findings from the current study. Our current study establish that (1) Tpz1-Ccq1 interaction is essential for Ccq1 Thr93 phosphorylation and telomerase recruitment, (2) Tpz1-Poz1 interaction promotes efficient accumulation of Poz1 to inhibit Ccq1 Thr93 phosphorylation and telomerase recruitment, and (3) Tpz1-Ccq1 and Tpz1-Poz1 interactions are redundantly required for protection against telomere fusions.

In this study, we determined amino acid residues within two distinct C-terminal domains of Tpz1 that are responsible for mediating either Tpz1-Ccq1 or Tpz1-Poz1 interaction, and characterized how these interactions individually or in combination affect the ability of the shelterin complex to ensure telomere maintenance and protection in fission yeast. (Key findings are summarized in Figure 8). Our results indicated that disruption of Tpz1-Ccq1 interaction causes telomere phenotypes that are essentially identical to those of *ccq1Δ* cells (Figures 4, S3, and S5). Cells lacking Tpz1-Ccq1 interaction fail to efficiently recruit telomerase to telomeres, due to loss of Rad3^ATR^/Tel1^ATM^ kinase-dependent Ccq1 Thr93 phosphorylation (Figure 5C–E), which is essential for promoting Est1-Ccq1 interaction and telomerase recruitment [12] (Figure 8).

Although Ccq1 association with telomeres was reduced, significant amounts were still detectable in the absence of Tpz1-Ccq1 interaction (Figure 5B), implicating the existence of an alternative mechanism that allows recruitment of Ccq1 to telomeres. Ccq1 also interacts with the SHREC complex that facilitates heterochromatin formation at telomeres [40] and heterochromatin-dependent recruitment of Ccq1 has been proposed as a mechanism to allow recruitment of Pot1 to protect chromosome ends in HATTI survivor cells that lack telomere repeats at chromosome ends [51]. Thus, it is possible that the SHREC complex is responsible for allowing Ccq1 localization at telomeres in the absence of Tpz1-Ccq1 interaction. On the other hand, we cannot completely rule out the possibility that a weak residual Tpz1-Ccq1 interaction, as detected under less stringent (−His) Y2H assay conditions (Figure S2C) but not by co-IP experiments (Figure 3C), may still contribute to Ccq1 binding at telomeres. In any case, our results established that localization of Ccq1 at telomeres alone is not sufficient for telomerase recruitment to telomeres, due to the fact that Tpz1-Ccq1 interaction is essential for Rad3^ATR^/Tel1^ATM^-dependent phosphorylation of Ccq1 Thr93.

Furthermore, we have shown that disruption of Tpz1-Poz1 interaction causes a dramatic reduction in Poz1 association with telomeres (Figure 7C), and results in phenotypes that are essentially identical to those of *poz1Δ* cells, including strong induction of Ccq1 Thr93 phosphorylation (Figure 7E), enhanced telomerase recruitment (Figure 7D) and massive elongation of telomeres (Figure 6). Since we have previously shown that *poz1Δ* cells accumulate more RPA (Replication Protein A) and Rad3^ATR^-Rad26^ATRIP^ kinase complex at telomeres [36], it is likely that increased Ccq1 Thr93 phosphorylation in Tpz1-Poz1 interaction disruption mutant cells is also caused by a failure to limit the accumulation of Rad3^ATR^ kinase.

Consistent with the notion that Tpz1-Ccq1 disruption and Tpz1-Poz1 disruption cause telomere defects nearly identical to *ccq1Δ* and *poz1Δ* respectively, we also found that (1) simultaneous disruption of both Tpz1-Ccq1 and Tpz1-Poz1 interaction, (2) combining the Tpz1-Ccq1 disruption mutation with *poz1Δ*, and (3) combining the Tpz1-Poz1 disruption mutation with *ccq1Δ*, all cause a strong synergistic loss of telomere protection and immediate telomere fusion phenotype (Figures 4, 6, and S3D–E), much like in *ccq1Δ poz1Δ* cells [31]. Thus, our current study establishes that Tpz1-Poz1 and Tpz1-Ccq1 interactions redundantly fulfill the essential telomere protection function of the shelterin complex. Taken together, our data from current and previous studies [12], [36] suggest that the negative regulatory function of Tpz1-Poz1 interaction works upstream of Rad3^ATR^ kinase to limit Rad3^ATR^/Tel1^ATM^-dependent phosphorylation of Ccq1 Thr93, while Tpz1-Ccq1 interaction works downstream of Rad3^ATR^ kinase to facilitate Ccq1 Thr93 phosphorylation and telomerase recruitment (Figure 8).

While our studies were in progress, another study, which also identified distinct Tpz1 C-terminal domains critical for mediating either Tpz1-Ccq1 or Tpz1-Poz1 interactions, was published [43]. While their results agreed well with our current study for the domains and amino acid residues that promote Tpz1-Ccq1 or Tpz1-Poz1 interaction, our conclusions differ significantly with regard to the functional significance of these interactions for fission yeast telomere regulation.

While their ChIP analysis of a Tpz1-Ccq1 disruption mutant (*tpz1-L449A*) showed no effect on Ccq1 or Trt1^TERT^ association with telomeres [43], our current findings clearly showed that all Tpz1-Ccq1 interaction mutants (including *tpz1-L449A*) reduce Ccq1 binding and almost entirely eliminate Trt1^TERT^ recruitment at telomeres (Figure 5B–D). Even more strikingly, they reported that *tpz1-L449A poz1Δ* cells carry highly elongated telomeres [43], unlike our finding that *tpz1-L449A poz1Δ* and *tpz1-L449R poz1Δ* cells immediately lose telomeres and survive by circularizing their chromosomes (Figures 4C–D and S3D–E). Furthermore, their ChIP analysis also indicated that disruption of Tpz1-Poz1 does not affect localization of Poz1 (for *tpz1-I501R* and *tpz1-I501A,R505E*) or Trt1^TERT^ (for *tpz1-I501R*) at telomeres [43], in contrast to our current study, which found that Tpz1-Poz1 interaction promotes Poz1 localization and prevent telomerase recruitment by limiting Ccq1 Thr93 phosphorylation.

Unfortunately, we are not entirely sure why our findings are so different from the recent study, especially with regard to *tpz1-L449A* where both studies have in theory analyzed the effect of the same single amino acid mutation. A direct comparison of the Tpz1-Poz1 results is more complicated due to the fact that mutant alleles analyzed in two studies are not identical. However, we do note that our Tpz1-Poz1 disruption mutants (*tpz1-[1–485]* and *tpz1-W498R,I501R*) appears to destabilize Poz1 (Figure 3E), and behave essentially identical to *poz1Δ* cells. In contrast, their mutants showed significantly less telomere elongation than *poz1Δ* cells and did not affect Poz1 stability [43], raising the possibility that their mutants have retained residual Tpz1-Poz1 interaction not detected by their co-IP analysis. Another potential weakness of the previous study was that their ChIP data for Trt1^TERT^ localization was quantified with real-time PCR primers that anneal to the sub-telomeric sequence adjacent to telomeric repeats, even for *tpz1-I501A* cells, which carry long telomeres [43]. In contrast, we performed dot blot-based Trt1^TERT^ ChIP analysis and corrected for telomere length for *tpz1-W498R,I501R* cells.

In any case, our results are incompatible with the model proposed by Jun *et al.* [43], which suggested that Tpz1-Ccq1 interaction works “upstream” (rather than downstream as we propose here) of Tpz1-Poz1 interaction to overcome a “non-extendible” shelterin complex status that is defined by the fully connected Taz1-Rap1-Poz1-Tpz1-Pot1 linkage, based primarily on their observation that *tpz1-L449A poz1Δ* and *tpz1-L449A-I501R* cells carry highly elongated telomeres in their hand [43]. Furthermore, it should be noted that their proposed model did not even attempt to explain how the shelterin complex enforces late S-phase specific recruitment of telomerase to telomeres [35], or how it regulates Rad3^ATR^/Tel1^ATM^-dependent Ccq1 Thr93 phosphorylation [12], [50] to allow preferential recruitment of telomerase to shorter telomeres (Figure S9B) [36]. In contrast, our current model (Figure 8) [36] provides an explanation for all previous observations [12], [31], [36], [41], [49] with regard to how telomerase association and telomere extensions are controlled by the shelterin complex and Rad3^ATR^/Tel1^ATM^ kinases in fission yeast. Furthermore, since our detailed cell cycle ChIP analyses have recently identified Poz1 as a critical regulatory factor that promotes the timely arrival of the lagging strand DNA polymerase α at telomeres to limit accumulation of ssDNA and Rad3^ATR^ kinase in late S-phase [36], we suggest that Tpz1-Poz1 interaction-dependent localization of Poz1 to telomeres is required to negatively regulate telomere extension during late-S phase by ensuring proper coordination of leading and lagging strand synthesis at telomeres to limit Ccq1 Thr93 phosphorylation and telomerase recruitment (Figure 8) [36].

### Possible implications for regulation of telomere maintenance by the mammalian shelterin complex

While the shelterin complex in mammalian cells has been found to negatively regulate the DNA damage checkpoint kinases ATM and ATR [26], [27], ATM and ATR kinases also contribute to telomere protection and maintenance [52]–[54]. Furthermore, much like in fission yeast, ATM and ATR specifically associate with telomeres during S/G_2_-phases [55], and lagging strand synthesis at mammalian telomeres is significantly delayed compared to leading strand synthesis [56]–[58]. In addition, mammalian TPP1 (Tpz1 ortholog), in collaboration with TRF1, TIN2 and POT1, has been shown to play both positive and negative roles in extension of telomeres [18], [19], [22]–, much like Tpz1 in fission yeast.

Studies have also found that TIN2 plays a major role in POT1-dependent inhibition of ATR activation at telomeres [60]. Therefore, TIN2-TPP1 interaction (much like Poz1-Tpz1 interaction) is also likely to function upstream of ATR kinase to ensure telomere stability. Since the mammalian shelterin complex subunit TRF1 (much like Taz1) has been found to promote semi-conservative replication of telomeres by replicative DNA polymerases [36], [61], [62], TRF1-TIN2-TPP1 interaction might control accumulation of ATM and ATR kinases at telomeres by regulating ssDNA accumulation at telomeres by coordinating leading and lagging strand synthesis. Thus, our current findings, which established roles of Tpz1-Poz1 and Tpz1-Ccq1 interactions in regulating Rad3^ATR^/Tel1^ATM^-dependent Ccq1 Thr93 phosphorylation and telomerase recruitment, may be relevant in understanding how the mammalian shelterin complex can collaborate with DNA damage checkpoint kinases to ensure telomere maintenance.

## Materials and Methods

### Yeast strains and plasmids

Fission yeast and budding yeast strains were generated and cultured using standard techniques and protocols [63], [64]. Growth analysis of serially diluted liquid cultures was performed as previously described [45] with cells diluted to 4×10^4^ cells/ml every 24 hours in YES (Yeast Extract Supplemented) media [63]. Effects of various *tpz1* mutations on telomere heterochromatin formation were monitored by utilizing *his3^+^::tel(1L)* strains that carry an integrated *his3^+^* marker adjacent to telomere repeats of chromosome I left arm, as previously described [48].

Fission yeast strains used in this study are listed in Table S2. For *ccq1Δ::hphMX*, *poz1Δ::natMX6*, *rap1Δ::ura4^+^*, and *trt1Δ::his3^+^*, original deletion strains were described previously [4], [38], [49], [65]. For *pot1Δ::natMX*, the drug resistance marker of *pot1Δ::kanMX* strain [33] was changed by transformation of a *natMX* fragment. For *tpz1-myc*, *trt1-myc*, *poz1-myc*, *ccq1-myc*, *ccq1-FLAG*, *pot1-FLAG* and *chk1-myc*, original tagged strains were described previously [31], [35], [49], [66], [67]. For *poz1-FLAG*, an epitope tag was introduced by PCR-based method [68]. Various myc-tagged *tpz1* mutant alleles were integrated at the endogenous *tpz1^+^* locus using a BsgI-BglI *tpz1-myc-kanMX* fragment, excised from mutagenized pBS-tpz1-13myc-kanMX plasmids listed in Table S3. Point mutations were introduced by Phusion site-directed mutagenesis (NEB, F-541) or QuikChange Lightning site-directed mutagenesis (Agilent, *210513*). Strains carrying various *tpz1* mutations without epitope tags were subsequently generated by transforming *tpz1-myc* mutant strains with a C-terminal *tpz1* fragment marked with *hphMX* within the 3′ untranslated region of the *tpz1^+^* gene.

Yeast two-hybrid (Y2H) assays were performed by mating *Saccharomyces cerevisiae MAT*a (Y2HGold: *MAT*a *trp1-901 leu2-3,-112 ura3-52 his3-200 LYS2::GAL1(UAS)-GAL1(TATA)-HIS3 GAL2(UAS)-GAL2(TATA)-ADE2 gal4Δ gal80Δ URA3::MEL1(UAS)-MEL1(TATA)-AUR1-C MEL1*) strains harboring GAL4-DBD (DNA-binding domain) plasmids with *MAT*α (Y187: *MAT*α *trp1-901 leu2-3,-112 ura3-52 his3-200 ade2-101 gal4Δ gal80Δ met^−^ URA3::GAL1(UAS)-GAL1(TATA)-LacZ MEL1*) strains harboring GAL4-AD (activation domain) plasmids, as described in the MATCHMAKER system manual (Clontech). Plasmids used in Y2H assays are listed in Table S4. Positive Y2H interactions were identified by spotting mated cells onto SD−HTL (−His) or SD−HTLA (−His −Ade) plates. To increase selection stringency, 1 mM or 5 mM 3-amino-1,2,4-triazole (3AT) was added to SD−HTL plates.

### Co-immunoprecipitation (co-IP) and western blot analysis

Co-IP experiments were performed as previously described [12], [49]. Briefly, cell extracts were prepared in lysis buffer [50 mM Tris pH 8.0, 150 mM NaCl, 10% glycerol, 5 mM EDTA, 0.5% NP40, 50 mM NaF, 1 mM DTT, 1 mM PMSF, 0.2 mM APMSF, 1 mM Na_3_VO_4_, ‘Complete’ protease inhibitor cocktail] using glass beads. For low salt co-IP experiment shown in Figure S3, lysis buffer contained 60 mM NaCl instead of 150 mM. Proteins were immunoprecipitated using either monoclonal anti-myc antibody (9B11, Cell Signaling) or monoclonal anti-FLAG antibody (M2-F1804, Sigma), and Dynabeads Protein G (Life Technologies), and washed 3× in lysis buffer. Immunoprecipitated proteins were analyzed by western blot analysis using monoclonal anti-FLAG or anti-myc as primary antibodies. In addition, anti-Cdc2 (y100.4, Abcam) was used to detect Cdc2 in whole cell extract (WCE) to serve as loading control. To detect Ccq1 Thr93 phosphorylation by anti-phospho(S/T)Q (Phospho-(Ser/Thr) ATM/ATR substrate antibody (2851, Cell Signaling), Ccq1 was first immuno-purified from whole cell extract using anti-FLAG antibody as previously described [12]. Either horseradish peroxidase (HRP)-conjugated (goat) anti-mouse (Pierce, 31430) or HRP-conjugated (goat) anti-rabbit (Pierce, 31460) was used as the secondary antibody.

### Pulsed-field gel electrophoresis and Southern blot analysis

Pulsed-field gel electrophoresis of NotI-digested chromosomal DNA was separated on a 1% agarose gel as previously described [69] in 0.5xTAE buffer at 14°C, using the CHEF-DR III system (BioRad) at 6 V/cm (200 V) and a pulse time of 60 to 120 sec for 24 hours. For telomere length analysis by Southern blotting, EcoRI-digested genomic DNA was separated on a 1% agarose gel, transferred to nylon membrane, and hybridized to a P^32^-labeled telomeric DNA probe as previously described [69], [70].

### ChIP assay, quantitative PCR and dot blot analysis

ChIP assays were carried out as previously described [36], [49], [70]. Fission yeast cells were crosslinked with 1% formaldehyde for 20 min at room temperature, and then incubated with 125 mM glycine for 5 min at room temperature. Cell extracts were then prepared in lysis buffer [50 mM Hepes-KOH pH 7.5, 140 mM NaCl, 1 mM EDTA, 1% (v/v) Triton X-100, 0.1% (w/v) sodium deoxycholate, 1 mM PMSF, ‘Complete’ protease inhibitor cocktail] using glass beads, and sonicated in an ice bath (Misonix Sonicator 3000 with cup horn device) to obtain ∼500 bp DNA fragments. 1–2 mg lysate was prepared in 200–250 µl lysis buffer and 5 µl was set aside for input. 1 µg of monoclonal anti-Myc (9B11) antibody was added to the ChIP sample and incubated for two hours at 4°C, then 30 µl of Dynabeads Protein G (Life Technologies) were added and incubated for one hour at 4°C. IPs were washed 3× with 1 ml lysis buffer, and DNA was recovered from ChIP and input samples using Chelex 100 as previously described [36], [49], [70].

Quantitative real-time PCR was performed with primers annealing to a region adjacent to the telomeric repeat (jk380 5′-TAT TTC TTT ATT CAA CTT ACC GCA CTT C-3′ and jk381 5′-CAG TAG TGC AGT GTA TTA TGA TAA TTA AAA TGG-3′) [71] utilizing SYBR green to quantify PCR products. % Precipitated DNA values were calculated based on ΔCt between Input and IP samples with the following formula: [% Precipitated DNA] = [100*E^ΔCt^]/[D*R], where E = amplification efficiency of primer pairs, ΔCt = [Ct Input]−[Ct ChIP], D = [dilution factor of ChIP]/[dilution factor of Input] and R = [Sample volume used in IP]/[Sample volume set aside for Input control].

For dot blot-based analysis of ChIP samples, DNA was denatured in 0.4 M NaOH, 10 mM EDTA for 10 min at 100°C, then rapidly cooled on ice, and subsequently blotted onto Hybond XL membrane (GE Healthcare) using a Bio-Dot Microfiltration Apparatus (Bio-Rad), and hybridized to a P^32^-labeled telomeric DNA probe as previously described [36], [49], [70]. For cells carrying highly elongated telomeres, data were corrected for changes in telomere length as previously described [36] by using correction factors for telomere length, established by measuring the hybridization signal intensity of telomere versus rDNA repeats (telomere/rDNA) (Table S1). “Telomere length corrected” ChIP values were then calculated by multiplying the background subtracted % precipitated DNA values (raw % precipitated DNA – no tag control % precipitated DNA) with the correction factors, and normalized to values from wild-type cells. SEM of telomere length corrected ChIP (SEM_Q_) was calculated as previously described [36].

### Telomere primer pull-down assay

Biotin-DNA primer coated Streptavidin beads were prepared by mixing 30 µl Dynabeads M-280 Streptavidin (Invitrogen, 10 mg/ml) with 100 pmol of biotinylated oligonucleotide primers (C-oligo: 5′ Biotin-CGT AAC CGT AAC CCT GTA ACC TGT AAC CTG TAA CCG TGT AAC C 3′; G-oligo: 5′ Biotin-GGT TAC ACG GTT ACA GGT TAC AGG TTA CAG GGT TAC GGT TAC G 3′) in Binding Buffer BW [5 mM Tris, pH 7.5, 0.5 mM EDTA, 1M NaCl] for 15 min at RT. Beads were then washed 2× with 1 ml BW and resuspended in 30 µl lysis buffer LB [50 mM Tris pH 8.0, 150 mM NaCl, 10% glycerol, 5 mM EDTA, 0.5% NP40, 50 mM NaF, 1 mM DTT, 1 mM PMSF, 0.2 mM APMSF, 1 mM Na3VO4, ‘Complete’ protein inhibitor cocktail]. 2 mg of fission yeast whole cell extract, prepared in LB, was then incubated with 30 µl Biotin-primer coated beads for 2 h at 4°C, washed 3× with 1 ml LB, and finally resuspended in 25 µl 2× Laemmli sample buffer for subsequent western blot analysis.

## Supporting Information

Figure S1 Y2H mutational analysis of charged residues within Tpz1-Ccq1 and Tpz1-Poz1 interaction domains. (**A**) Sequence alignment of Tpz1 regions responsible for Tpz1-Ccq1 and Tpz1-Poz1 interaction in *S. pombe* and corresponding regions from three additional *Schizosaccharomyces* species (*S. octosporus*, *S. cryophilus* and *S. japonicus*). Conserved charged amino acids mutated for Y2H analysis are indicated. Blue diamonds indicate various truncation mutants of Tpz1. (See Figure 1C.) (**B**) Y2H assay for mutations that did not affect Tpz1-Ccq1 or Tpz1-Poz1 interaction. Indicated mutations were tested in full length Tpz1 (Tpz1-FL). For charged residues (Lys, Arg, Asp and Glu), either a charge swap (Lys/Arg to Glu) or an Alanine mutation (Lys/Arg/Asp/Glu to Ala) was introduced. Positive interactions were identified by growth on −His −Ade and −His +5 mM 3AT plates.
(JPG)

Figure S2 Y2H analysis of hydrophobic to alanine mutations within Tpz1-Ccq1 and Tpz1-Poz1 interaction domains. (**A**) A schematic representation of Tpz1, marked with Ccq1 and Poz1 interaction domains. (**B**) Y2H assay for alanine mutations of conserved hydrophobic residues within Tpz1-Ccq1 or Tpz1-Poz1 interaction domains. Indicated mutations were tested in full length Tpz1 (Tpz1-FL). Positive interactions were identified by growth on −His −Ade and −His +5 mM 3AT plates. (**C**) Y2H assay comparison of Ccq1-Tpz1 interaction for wild-type (wt) Tpz1, Tpz1-L449R and Tpz1-L449A. 5-fold serial dilution series were spotted on indicated plates. For all selection plates tested, L449A showed a milder defect on Tpz1-Ccq1 Y2H interaction than L449R.
(JPG)

Figure S3 Comparison of *tpz1-L449R* and *tpz1-L449A* mutant alleles. (**A**) Examination of Tpz1-Ccq1 interaction by co-IP in less stringent (60 mM NaCl) or our standard (150 mM NaCl) lysis/wash conditions. We found that L449R and L449A are equally effective in disrupting Tpz1-Ccq1 interaction detected in co-IP experiments. (**B**) Southern blot analysis of telomere length for *tpz1-L449A* cells. Haploid cells were generated by dissection of spores derived from heterozygous *tpz1^+^*/mutated *tpz1* diploid cells, and restreaked twice or 5 times on plates (estimated to be 40–50 or 100–125 cell divisions, respectively) prior to preparation of genomic DNA. For each round of restreak, several faster growing colonies were combined and streaked for single colonies on YES plates. (**C**) Pulsed-field gel analysis of telomere fusions for early generation small colonies of *tpz1-L449A* cells, which showed I+L fusion band as well as much fainter bands for I+M, L+M, I, L and M bands for two out of four clones tested. (See Figure 4B for a NotI-restriction site map of fission yeast chromosomes.) Compared to the *tpz1-L449R* mutant shown in Figure 4B, tpz1-*L449A* showed less prominent early telomere fusions. (**D**–**E**) Epistasis analysis for telomere loss by Southern blot (D) or telomere fusion by pulsed-field gel (E) indicated that both *L449A* and *L449R* alleles show synergistic telomere loss and fusion phenotypes in *poz1Δ* cells. Samples were prepared from early generation cells after strains were generated by genetic cross of parental haploid strains and dissection of resulting double mutant spores.
(JPG)

Figure S4 Effects of Tpz1-Ccq1 interaction disruption mutations on telomere maintenance. Southern blot analysis of telomere length for indicated Tpz1-Ccq1 interaction disruption mutants. Multiple independent haploid cells were generated by dissection of spores derived from heterozygous *tpz1^+^*/mutated *tpz1* diploid cells, and restreaked 11 times (estimated to be 220–275 cell divisions) on plates prior to preparation of genomic DNA. For each round of restreak, several faster growing colonies were combined and streaked for single colonies on YES plates.
(JPG)

Figure S5 Liquid culture growth assay and Southern blot telomere analysis of Tpz1-Ccq1 interaction disruption mutant L449R. Wild-type (wt) (**A**), *trt1Δ* (**B**), *ccq1Δ* (**C**) and *tpz1-L449R* (**D**) haploid colonies (freshly derived by dissection of spores derived from heterozygous diploid cells) were inoculated and subsequently serially diluted to 4×10^4^ cells/ml every 24 hours in fresh YES. Cell densities were counted after 24 hours and plotted (left panels). For Southern blot analysis (right panels), cells from two cultures for each genotype were harvested and genomic DNA was prepared for days indicated.
(JPG)

Figure S6 Tpz1-Ccq1 interaction is necessary to prevent checkpoint activation. (**A**) Microscopic analysis of early generation (20–40 cell divisions) wild-type (wt), *ccq1Δ* and *tpz1*-mutant cells grown in liquid YES culture. White scale bar indicates 10 µm. (**B**) Western blot analysis of whole cell extracts from wild-type, *ccq1*Δ and *tpz1*-mutant cells expressing Chk1-myc. Wild-type cells were also exposed to 100 Gy of gamma-irradiation to induce DNA damage and Chk1 phosphorylation (*).
(JPG)

Figure S7 Heterochromatin formation at telomeres is affected in Tpz1-Ccq1 and Tpz1-Poz1 interaction mutants. Wild-type, *ccq1Δ*, *poz1Δ* and *tpz1*-mutant cells carrying the *his3^+^* marker gene directly adjacent to the chromosome 1L telomere repeats were serially diluted and spotted on YES (no selection) or PMG ULA (**−**histidine) plates. (**A**) Similar to *ccq1Δ*, all Tpz1-Ccq1 interaction disruption mutations caused loss of transcriptional silencing at telomeres. (**B**) Similar to *poz1Δ*, all Tpz1-Poz1 interaction disruption mutations caused loss of transcriptional silencing at telomeres.
(JPG)

Figure S8 Characterization of Tpz1-Ccq1 interaction disruption mutant cells. (**A**–**C**) Southern blot analysis of early generation mutant strains (20–40 cell divisions) utilized in ChIP analysis (Figure 5). (**D**) Binding of Tpz1 to telomerase RNA (*TER1*) was lost in Tpz1-Ccq1 interaction disruption mutants. Error bars represent standard error of the mean from three to four independent experiments. Statistical analysis of *TER1* co-IP data by 2-tailed Student's t-test is shown in Table S5. Expression of Tpz1 was examined by anti-myc western blot, and Cdc2 served as loading control.
(JPG)

Figure S9 Comparison of *tpz1-L449A* and *tpz1-L449R* alleles for their effects on Ccq1 and Trt1^TERT^ localization to telomeres. (**A**) Based on ChIP assay, Ccq1 showed comparable reduction in telomere association for both *L449A* and *L449R* alleles. (**B**) Comparable loss of Trt1 binding to telomeres was observed by ChIP assay for *ccq1Δ*, *tpz1-L449A* and *tpz1-L449R* cells. In contrast to these mutant cells that show telomere shortening due to loss of telomerase recruitment, catalytically dead Trt1 (*trt1-D743A*) showed increased binding at shorter telomeres [36]. Error bars represent standard error of the mean from three to eight independent experiments. Statistical analysis of ChIP data by 2-tailed Student's t-test is shown in Table S5.
(JPG)

Figure S10 Effects of Tpz1 truncations on telomere maintenance. (**A**) A schematic overview of Tpz1 truncation mutants. Minimum Ccq1 and Poz1 interaction domains are indicated. (**B**) Anti-myc western blot analysis to detect expression levels of myc-tagged wild-type and *tpz1*-truncation mutants in whole cell extract. Cdc2 served as loading control. (**C**) Southern blot analysis of wild-type and indicated truncation mutants hybridized to a telomeric repeat probe. For tpz1 truncation mutants, haploid cells derived from heterozygous diploid cells were restreaked 5 times on YES plates (estimated to be 100–125 cell divisions) prior to preparation of genomic DNA. (**D**) Pulsed-field gel analysis of wild-type and indicated *tpz1*-truncation mutants. Chromosomal DNA was prepared from cells after restreaked twice on YES plates (estimated to be 40–50 cell divisions), and telomeric NotI fragments were visualized by hybridization to C, I, L and M-specific probes. (See Figure 4B for a NotI-restriction site map of fission yeast chromosomes.)
(JPG)

Figure S11 Binding of the Tpz1-Pot1 complex to telomere oligo primer does not depend on Tpz1-Ccq1 or Tpz1-Poz1 interaction. (**A**) A schematic overview for the telomere oligonucleotide primer pull-down assay. Biotin-conjugated primers were bound to streptavidin-conjugated magnetic beads, and incubated with whole cell extracts from cells to monitor Pot1-dependent binding to Tpz1. (**B**) Tpz1 specifically associated with the telomeric G-oligo but not the complementary telomeric C-oligo. The interaction of Tpz1 with G-oligo was lost in *pot1*Δ cells. (See Materials and Methods.) (**C**) Tpz1-Pot1 interaction was not affected by Tpz1-Ccq1 or Tpz1-Poz1 interaction disruption mutants. (**D**) Tpz1-Pot1 interaction remained intact even in *ccq1*Δ *poz1*Δ cells. Interaction between Tpz1-myc and Pot1-FLAG was monitored by co-IP of Pot1-FLAG after anti-myc pull down of Tpz1-myc. For whole cell extract (WCE) western blot, Cdc2 served as a loading control.
(JPG)

Figure S12 Characterization of Tpz1-Poz1 interaction disruption mutant cells. Telomere length analysis by Southern blot was performed for strains used in (**A**) Tpz1, (**B**) Ccq1, (**C**) Poz1, and (**D**) Trt1^TERT^ ChIP assays (Figures 7A–D and S13).
(JPG)

Figure S13 Raw data for Tpz1, Ccq1, Poz1 and Trt1^TERT^ ChIP assays in Tpz1-Poz1 interaction mutant cells. Effects of disrupting Tpz1-Poz1 interaction on telomere association for (**A**) Tpz1, (**B**) Ccq1, (**C**) Poz1 and (**D**) Trt1^TERT^ were monitored by dot-blot ChIP assays and raw % precipitated DNA values were plotted. These data were then corrected for telomere length [36] to generate plots shown in Figure 7. Error bars represent standard error of the mean from three to eight independent experiments. Statistical analysis of ChIP data by 2-tailed Student's t-test is shown in Table S5.
(JPG)

Table S1 Telomere length correction factors (telomere/rDNA).
(PDF)

Table S2 Fission yeast strains used in this study.
(PDF)

Table S3 Plasmids used to integrate *tpz1* mutant alleles into fission yeast.
(PDF)

Table S4 Plasmids used in yeast 2-hybrid assays.
(PDF)

Table S5 Statistical analysis of ChIP and *TER1* co-IP data by 2-tailed Student's t-test.
(PDF)

Supporting Information S1 A single PDF file containing all Supporting Information (Figures S1, S2, S3, S4, S5, S6, S7, S8, S9, S10, S11, S12, S13 and Tables S1, S2, S3, S4, S5).
(PDF)
