# Supplementary figures and images for: Tpz1-Ccq1 and Tpz1-Poz1 Interactions within Fission Yeast Shelterin Modulate Ccq1 Thr93 Phosphorylation and Telomerase Recruitment
Source: PLoS Genet. 2014 Oct 16;10(10):e1004708. doi: 10.1371/journal.pgen.1004708 (PMC4199508; doi:10.1371/journal.pgen.1004708)

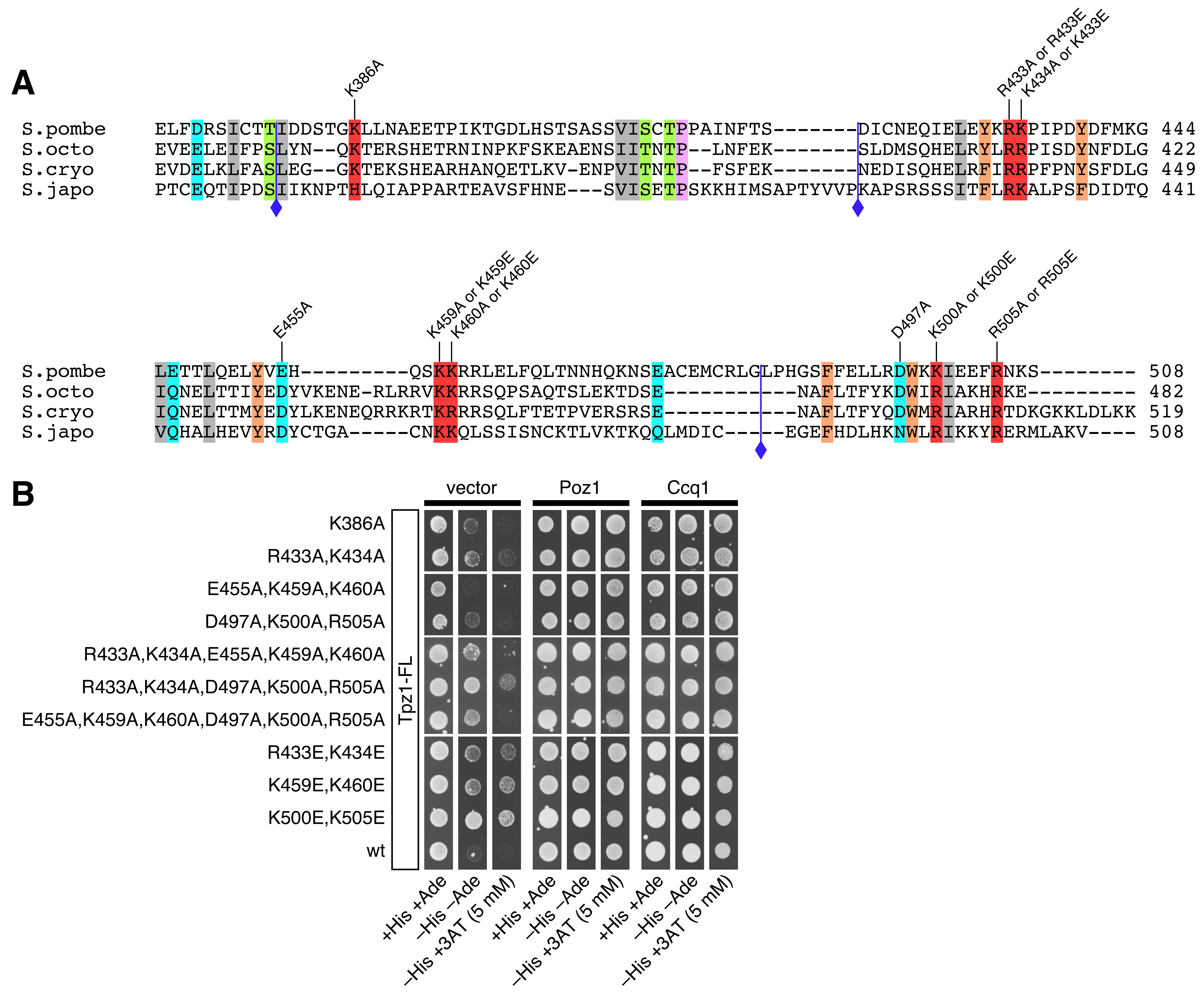

Supplement: Figure S1 — Y2H mutational analysis of charged residues within Tpz1-Ccq1 and Tpz1-Poz1 interaction domains. (A) Sequence alignment of Tpz1 regions responsible for Tpz1-Ccq1 and Tpz1-Poz1 interaction in S. pombe and corresponding regions from three additional Schizosaccharomyces species (S. octosporus, S. cryophilus and S. japonicus). Conserved charged amino acids mutated for Y2H analysis are indicated. Blue diamonds indicate various truncation mutants of Tpz1. (See Figure 1C.) (B) Y2H assay for mutations that did not affect Tpz1-Ccq1 or Tpz1-Poz1 interaction. Indicated mutations were tested in full length Tpz1 (Tpz1-FL). For charged residues (Lys, Arg, Asp and Glu), either a charge swap (Lys/Arg to Glu) or an Alanine mutation (Lys/Arg/Asp/Glu to Ala) was introduced. Positive interactions were identified by growth on −His −Ade and −His +5 mM 3AT plates. (JPG) [file pgen.1004708.s001.jpg]

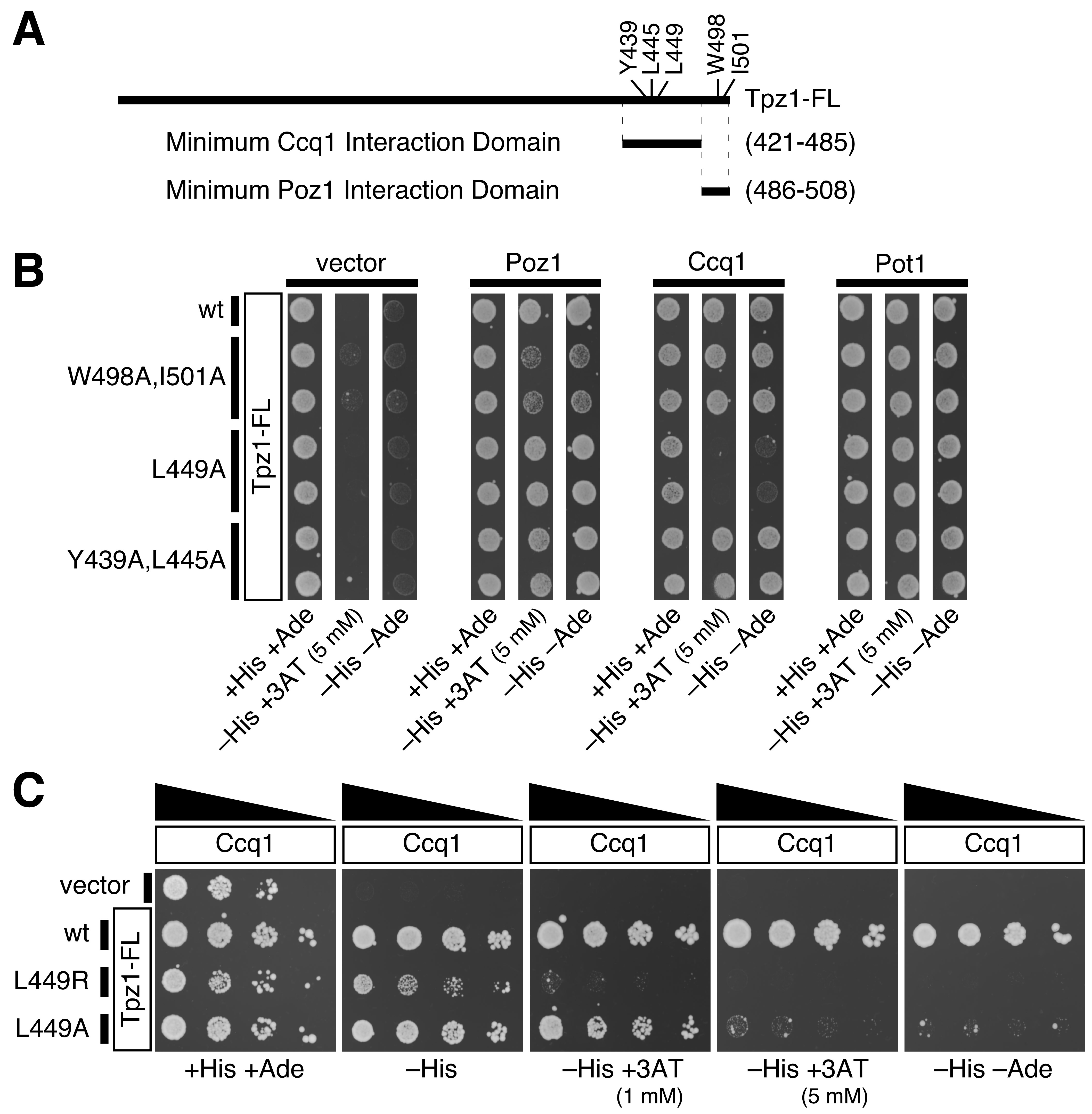

Supplement: Figure S2 — Y2H analysis of hydrophobic to alanine mutations within Tpz1-Ccq1 and Tpz1-Poz1 interaction domains. (A) A schematic representation of Tpz1, marked with Ccq1 and Poz1 interaction domains. (B) Y2H assay for alanine mutations of conserved hydrophobic residues within Tpz1-Ccq1 or Tpz1-Poz1 interaction domains. Indicated mutations were tested in full length Tpz1 (Tpz1-FL). Positive interactions were identified by growth on −His −Ade and −His +5 mM 3AT plates. (C) Y2H assay comparison of Ccq1-Tpz1 interaction for wild-type (wt) Tpz1, Tpz1-L449R and Tpz1-L449A. 5-fold serial dilution series were spotted on indicated plates. For all selection plates tested, L449A showed a milder defect on Tpz1-Ccq1 Y2H interaction than L449R. (JPG) [file pgen.1004708.s002.jpg]

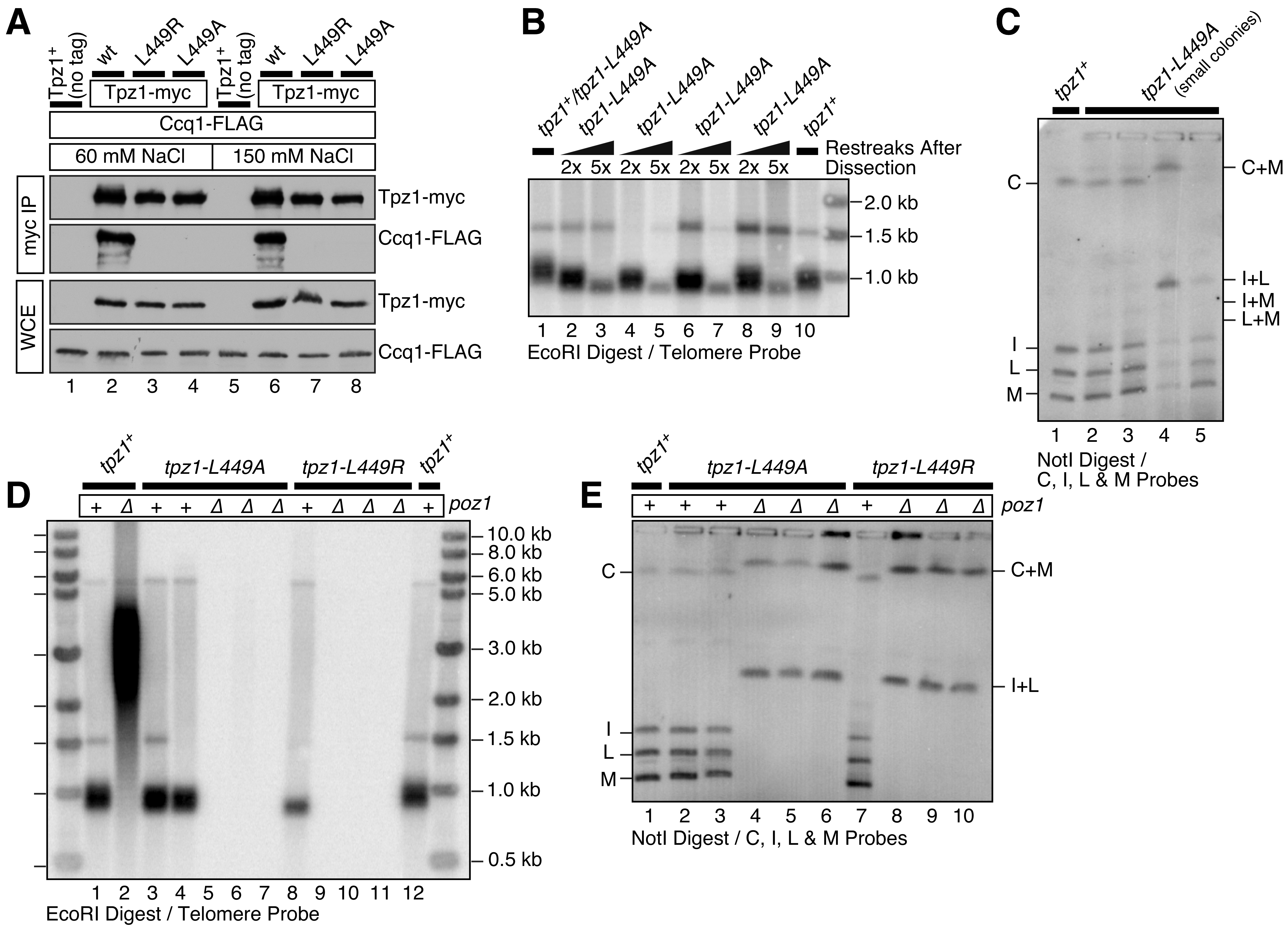

Supplement: Figure S3 — Comparison of tpz1-L449R and tpz1-L449A mutant alleles. (A) Examination of Tpz1-Ccq1 interaction by co-IP in less stringent (60 mM NaCl) or our standard (150 mM NaCl) lysis/wash conditions. We found that L449R and L449A are equally effective in disrupting Tpz1-Ccq1 interaction detected in co-IP experiments. (B) Southern blot analysis of telomere length for tpz1-L449A cells. Haploid cells were generated by dissection of spores derived from heterozygous tpz1+/mutated tpz1 diploid cells, and restreaked twice or 5 times on plates (estimated to be 40–50 or 100–125 cell divisions, respectively) prior to preparation of genomic DNA. For each round of restreak, several faster growing colonies were combined and streaked for single colonies on YES plates. (C) Pulsed-field gel analysis of telomere fusions for early generation small colonies of tpz1-L449A cells, which showed I+L fusion band as well as much fainter bands for I+M, L+M, I, L and M bands for two out of four clones tested. (See Figure 4B for a NotI-restriction site map of fission yeast chromosomes.) Compared to the tpz1-L449R mutant shown in Figure 4B, tpz1-L449A showed less prominent early telomere fusions. (D–E) Epistasis analysis for telomere loss by Southern blot (D) or telomere fusion by pulsed-field gel (E) indicated that both L449A and L449R alleles show synergistic telomere loss and fusion phenotypes in poz1Δ cells. Samples were prepared from early generation cells after strains were generated by genetic cross of parental haploid strains and dissection of resulting double mutant spores. (JPG) [file pgen.1004708.s003.jpg]

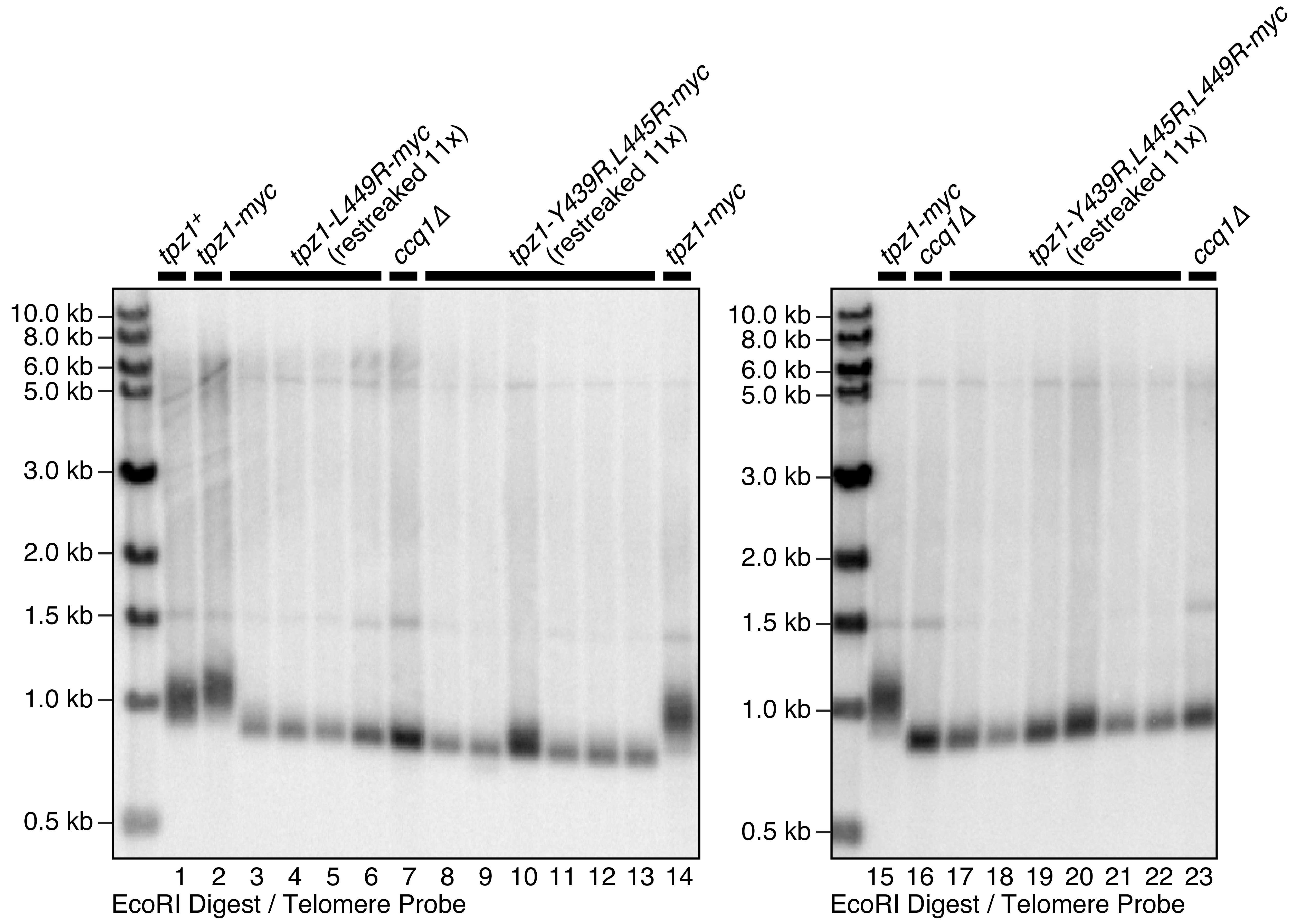

Supplement: Figure S4 — Effects of Tpz1-Ccq1 interaction disruption mutations on telomere maintenance. Southern blot analysis of telomere length for indicated Tpz1-Ccq1 interaction disruption mutants. Multiple independent haploid cells were generated by dissection of spores derived from heterozygous tpz1+/mutated tpz1 diploid cells, and restreaked 11 times (estimated to be 220–275 cell divisions) on plates prior to preparation of genomic DNA. For each round of restreak, several faster growing colonies were combined and streaked for single colonies on YES plates. (JPG) [file pgen.1004708.s004.jpg]

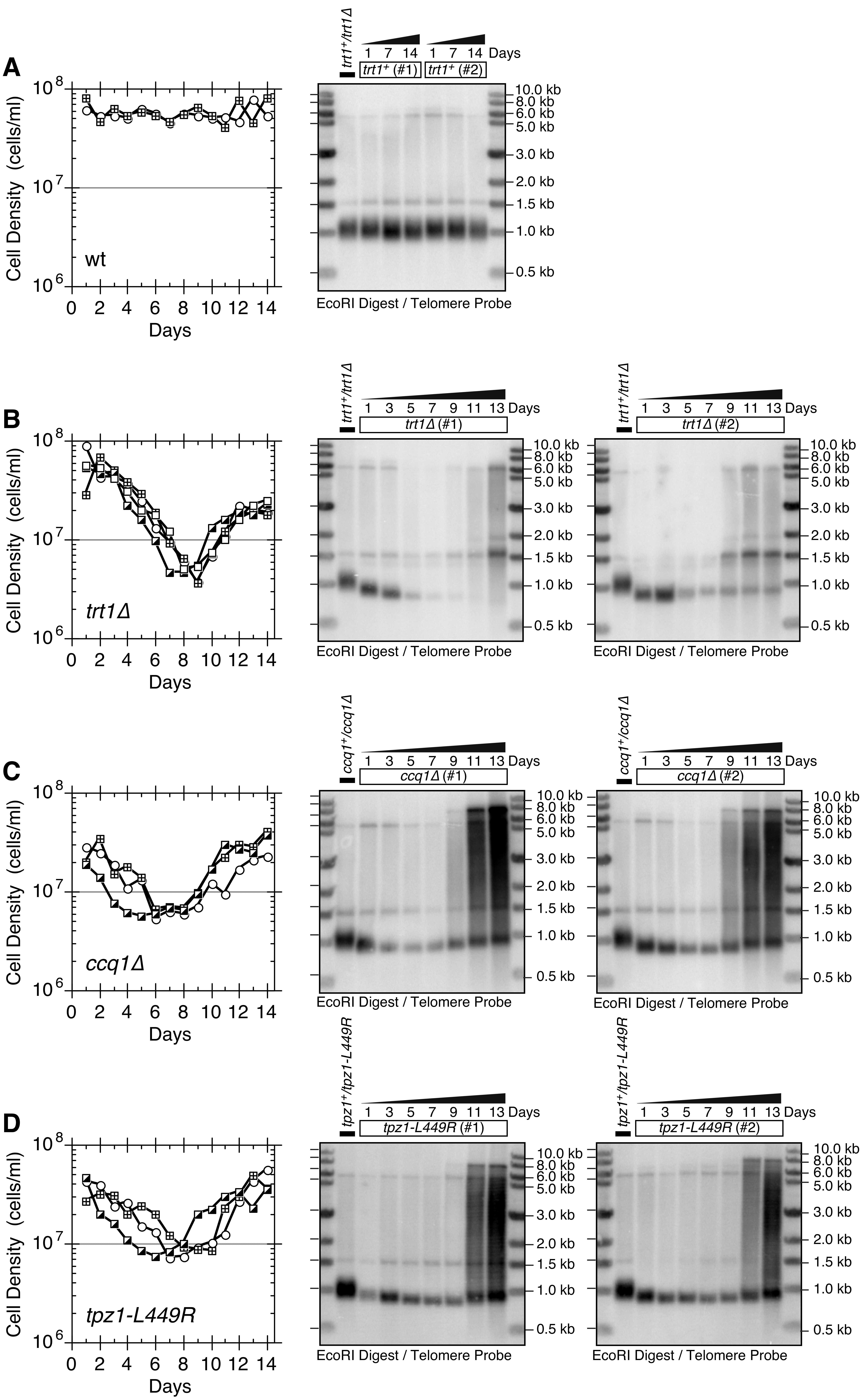

Supplement: Figure S5 — Liquid culture growth assay and Southern blot telomere analysis of Tpz1-Ccq1 interaction disruption mutant L449R. Wild-type (wt) (A), trt1Δ (B), ccq1Δ (C) and tpz1-L449R (D) haploid colonies (freshly derived by dissection of spores derived from heterozygous diploid cells) were inoculated and subsequently serially diluted to 4×10^4^ cells/ml every 24 hours in fresh YES. Cell densities were counted after 24 hours and plotted (left panels). For Southern blot analysis (right panels), cells from two cultures for each genotype were harvested and genomic DNA was prepared for days indicated. (JPG) [file pgen.1004708.s005.jpg]

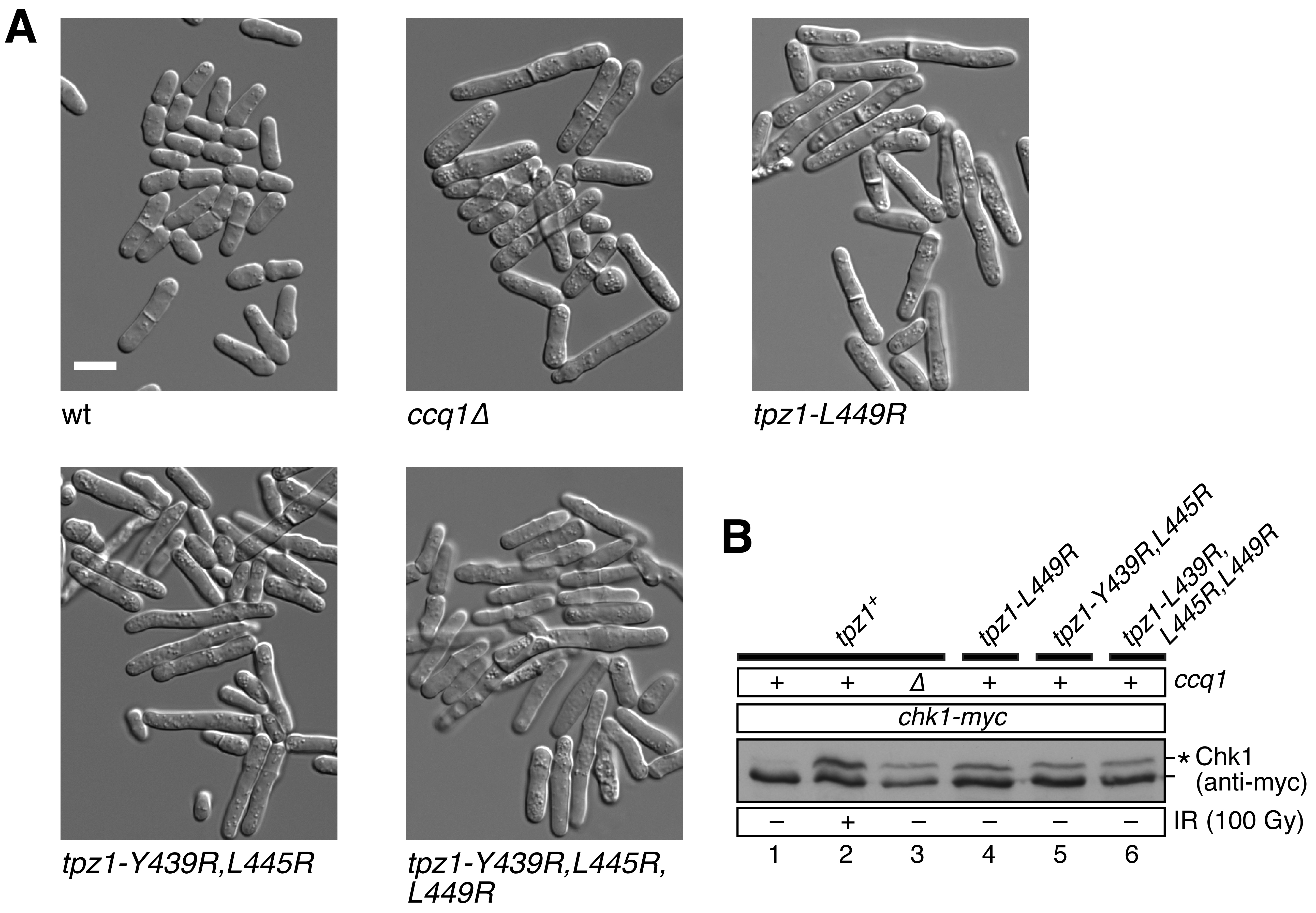

Supplement: Figure S6 — Tpz1-Ccq1 interaction is necessary to prevent checkpoint activation. (A) Microscopic analysis of early generation (20–40 cell divisions) wild-type (wt), ccq1Δ and tpz1-mutant cells grown in liquid YES culture. White scale bar indicates 10 µm. (B) Western blot analysis of whole cell extracts from wild-type, ccq1Δ and tpz1-mutant cells expressing Chk1-myc. Wild-type cells were also exposed to 100 Gy of gamma-irradiation to induce DNA damage and Chk1 phosphorylation (*). (JPG) [file pgen.1004708.s006.jpg]

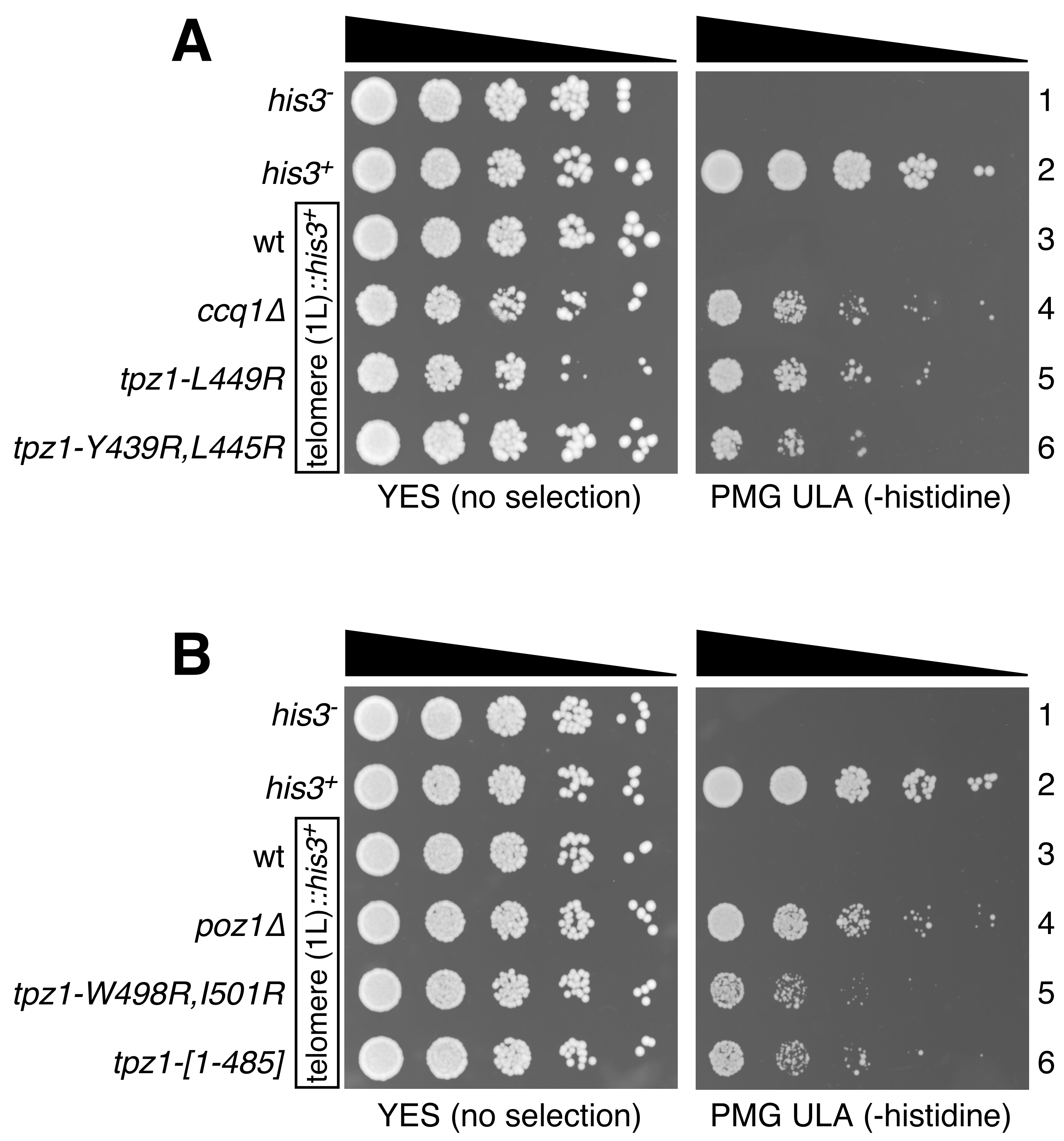

Supplement: Figure S7 — Heterochromatin formation at telomeres is affected in Tpz1-Ccq1 and Tpz1-Poz1 interaction mutants. Wild-type, ccq1Δ, poz1Δ and tpz1-mutant cells carrying the his3+ marker gene directly adjacent to the chromosome 1L telomere repeats were serially diluted and spotted on YES (no selection) or PMG ULA (−histidine) plates. (A) Similar to ccq1Δ, all Tpz1-Ccq1 interaction disruption mutations caused loss of transcriptional silencing at telomeres. (B) Similar to poz1Δ, all Tpz1-Poz1 interaction disruption mutations caused loss of transcriptional silencing at telomeres. (JPG) [file pgen.1004708.s007.jpg]

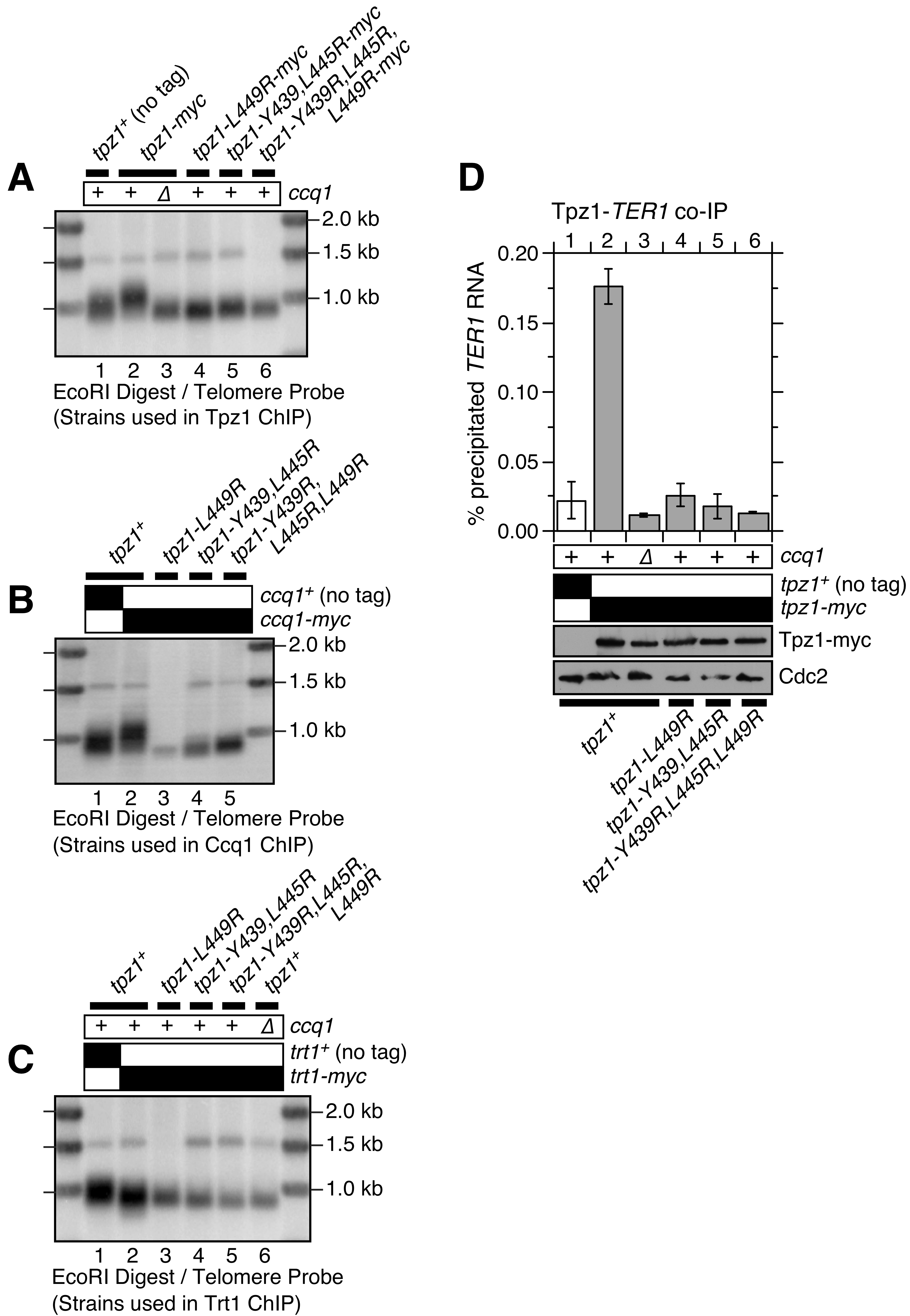

Supplement: Figure S8 — Characterization of Tpz1-Ccq1 interaction disruption mutant cells. (A–C) Southern blot analysis of early generation mutant strains (20–40 cell divisions) utilized in ChIP analysis (Figure 5). (D) Binding of Tpz1 to telomerase RNA (TER1) was lost in Tpz1-Ccq1 interaction disruption mutants. Error bars represent standard error of the mean from three to four independent experiments. Statistical analysis of TER1 co-IP data by 2-tailed Student's t-test is shown in Table S5. Expression of Tpz1 was examined by anti-myc western blot, and Cdc2 served as loading control. (JPG) [file pgen.1004708.s008.jpg]

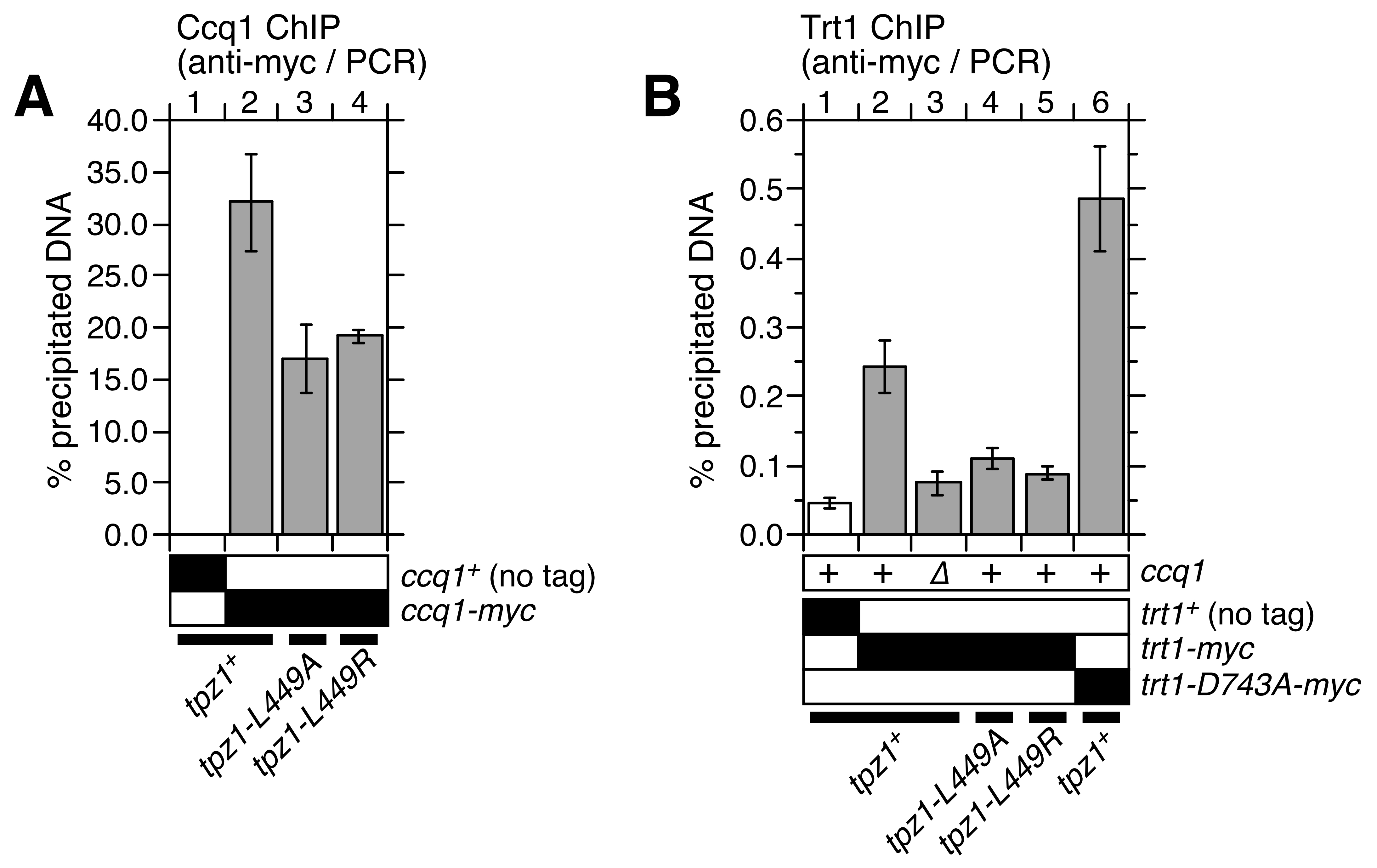

Supplement: Figure S9 — Comparison of tpz1-L449A and tpz1-L449R alleles for their effects on Ccq1 and Trt1TERT localization to telomeres. (A) Based on ChIP assay, Ccq1 showed comparable reduction in telomere association for both L449A and L449R alleles. (B) Comparable loss of Trt1 binding to telomeres was observed by ChIP assay for ccq1Δ, tpz1-L449A and tpz1-L449R cells. In contrast to these mutant cells that show telomere shortening due to loss of telomerase recruitment, catalytically dead Trt1 (trt1-D743A) showed increased binding at shorter telomeres [36]. Error bars represent standard error of the mean from three to eight independent experiments. Statistical analysis of ChIP data by 2-tailed Student's t-test is shown in Table S5. (JPG) [file pgen.1004708.s009.jpg]

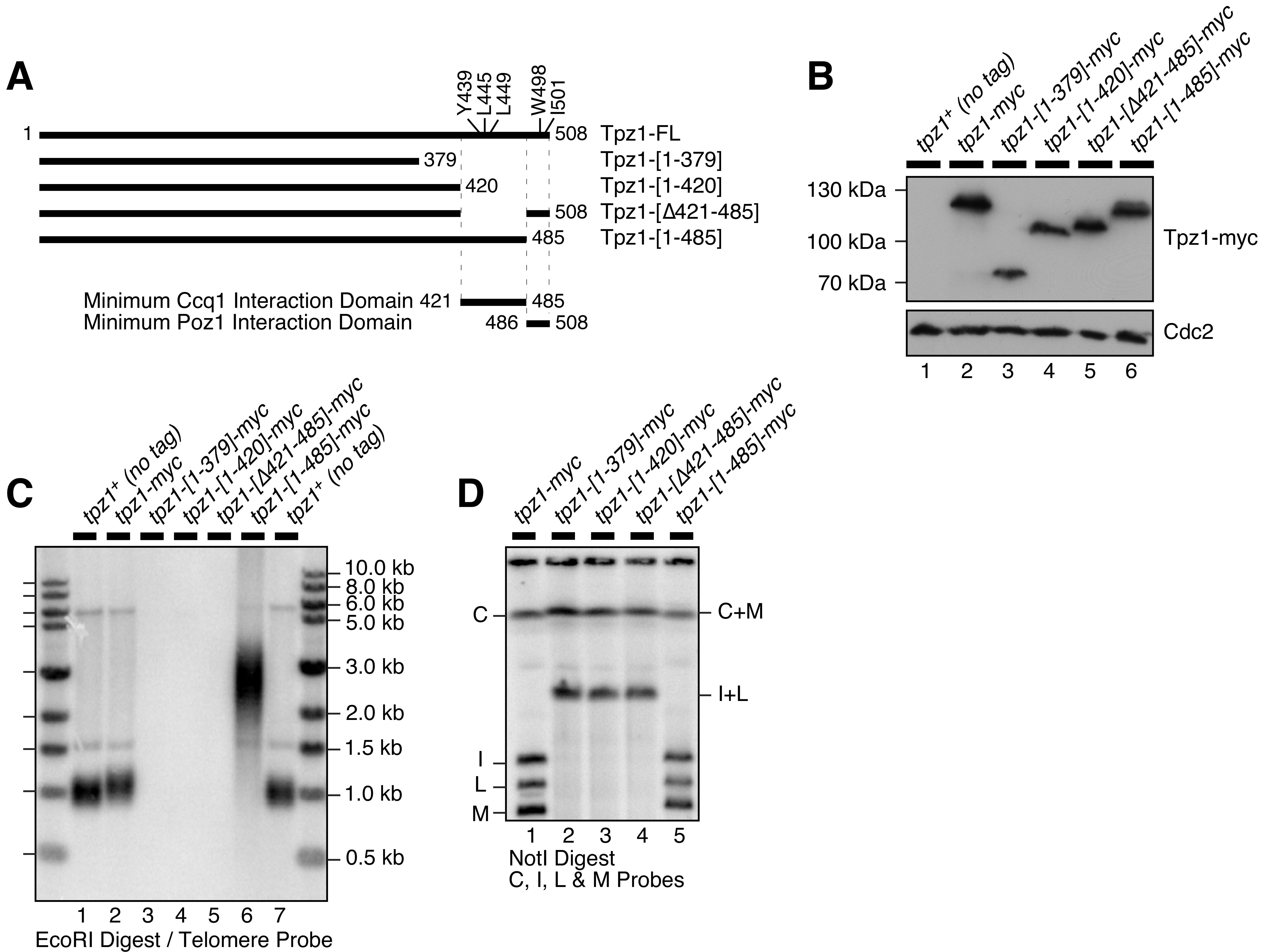

Supplement: Figure S10 — Effects of Tpz1 truncations on telomere maintenance. (A) A schematic overview of Tpz1 truncation mutants. Minimum Ccq1 and Poz1 interaction domains are indicated. (B) Anti-myc western blot analysis to detect expression levels of myc-tagged wild-type and tpz1-truncation mutants in whole cell extract. Cdc2 served as loading control. (C) Southern blot analysis of wild-type and indicated truncation mutants hybridized to a telomeric repeat probe. For tpz1 truncation mutants, haploid cells derived from heterozygous diploid cells were restreaked 5 times on YES plates (estimated to be 100–125 cell divisions) prior to preparation of genomic DNA. (D) Pulsed-field gel analysis of wild-type and indicated tpz1-truncation mutants. Chromosomal DNA was prepared from cells after restreaked twice on YES plates (estimated to be 40–50 cell divisions), and telomeric NotI fragments were visualized by hybridization to C, I, L and M-specific probes. (See Figure 4B for a NotI-restriction site map of fission yeast chromosomes.) (JPG) [file pgen.1004708.s010.jpg]

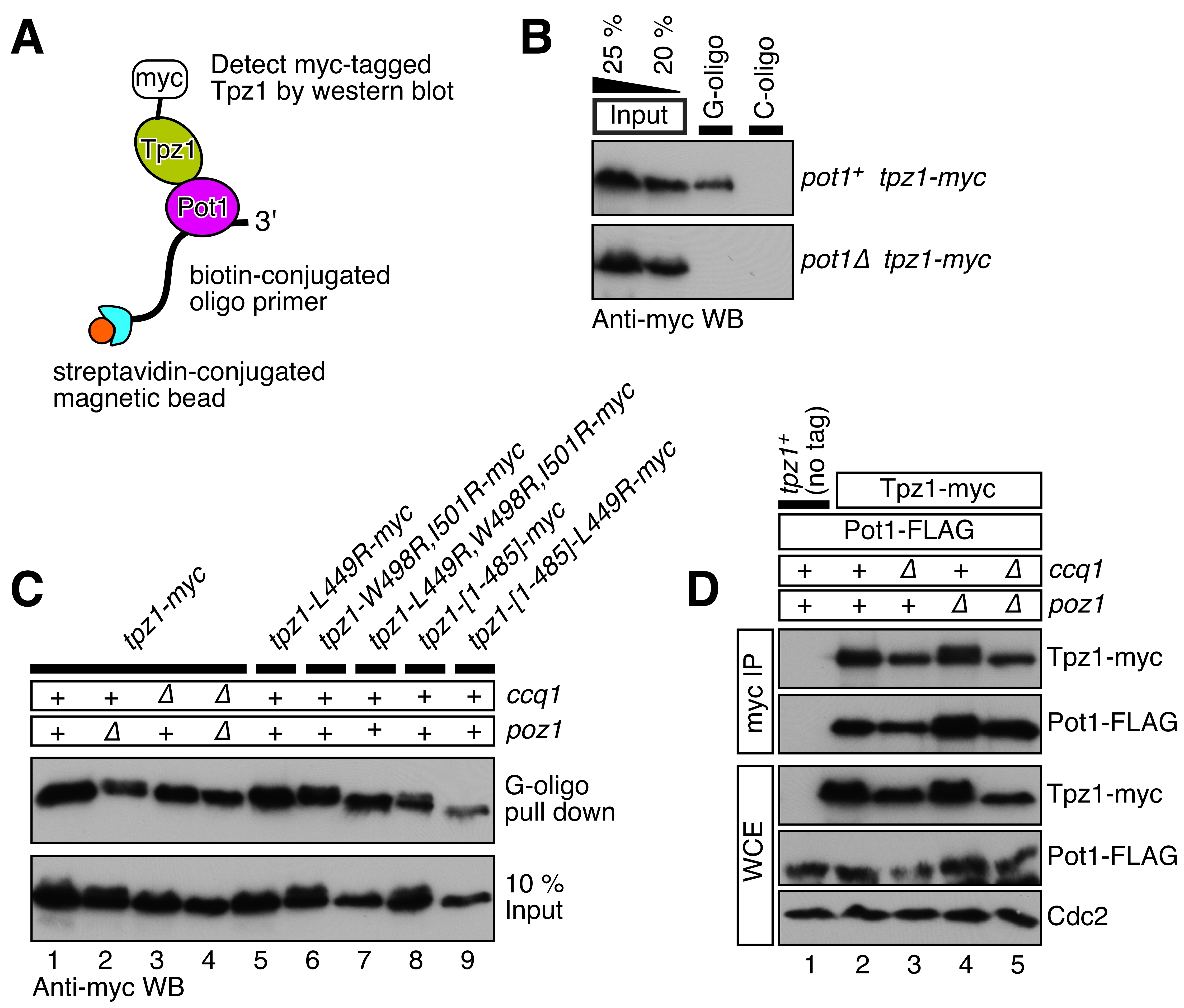

Supplement: Figure S11 — Binding of the Tpz1-Pot1 complex to telomere oligo primer does not depend on Tpz1-Ccq1 or Tpz1-Poz1 interaction. (A) A schematic overview for the telomere oligonucleotide primer pull-down assay. Biotin-conjugated primers were bound to streptavidin-conjugated magnetic beads, and incubated with whole cell extracts from cells to monitor Pot1-dependent binding to Tpz1. (B) Tpz1 specifically associated with the telomeric G-oligo but not the complementary telomeric C-oligo. The interaction of Tpz1 with G-oligo was lost in pot1Δ cells. (See Materials and Methods.) (C) Tpz1-Pot1 interaction was not affected by Tpz1-Ccq1 or Tpz1-Poz1 interaction disruption mutants. (D) Tpz1-Pot1 interaction remained intact even in ccq1Δ poz1Δ cells. Interaction between Tpz1-myc and Pot1-FLAG was monitored by co-IP of Pot1-FLAG after anti-myc pull down of Tpz1-myc. For whole cell extract (WCE) western blot, Cdc2 served as a loading control. (JPG) [file pgen.1004708.s011.jpg]

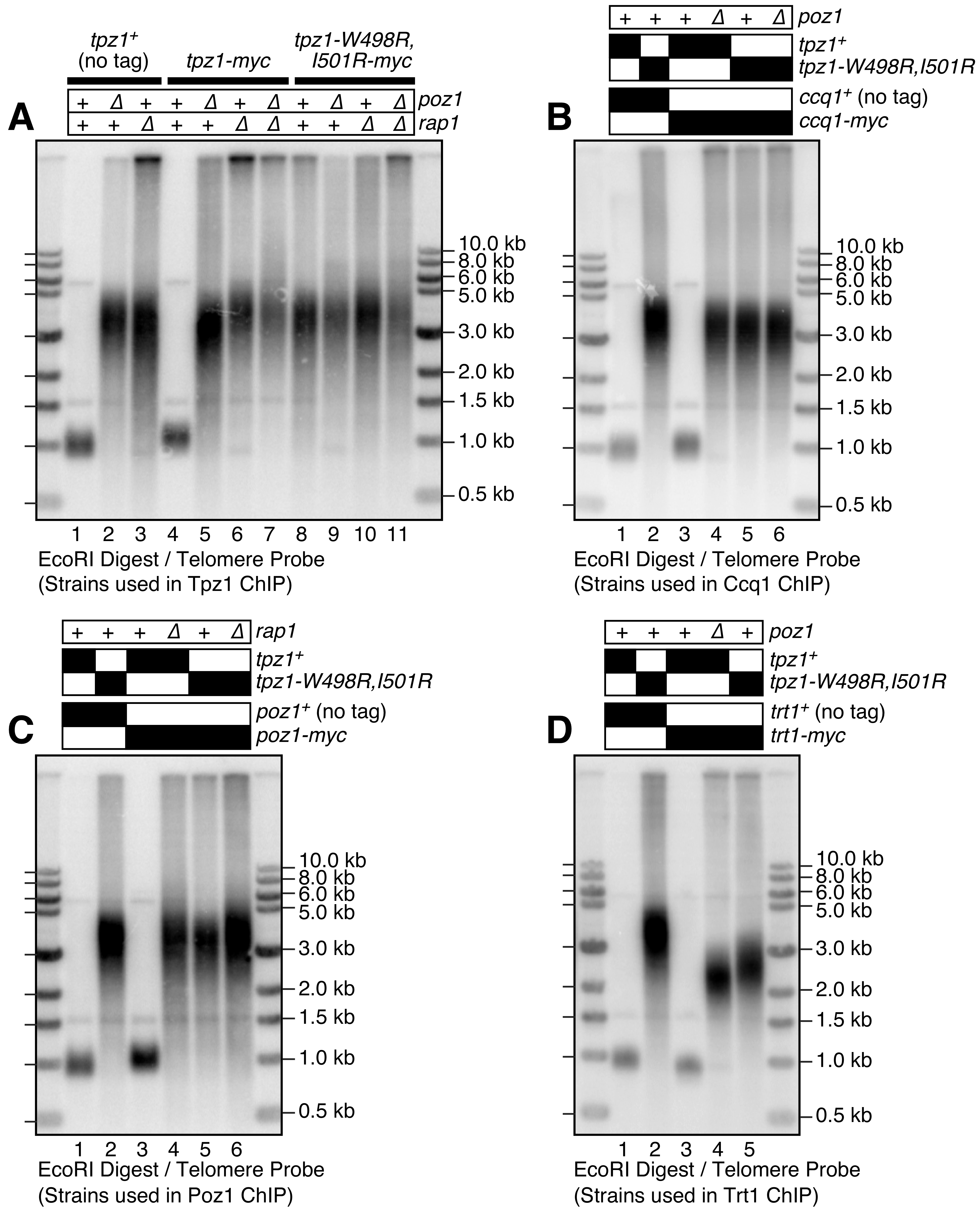

Supplement: Figure S12 — Characterization of Tpz1-Poz1 interaction disruption mutant cells. Telomere length analysis by Southern blot was performed for strains used in (A) Tpz1, (B) Ccq1, (C) Poz1, and (D) Trt1TERT ChIP assays (Figures 7A–D and S13). (JPG) [file pgen.1004708.s012.jpg]

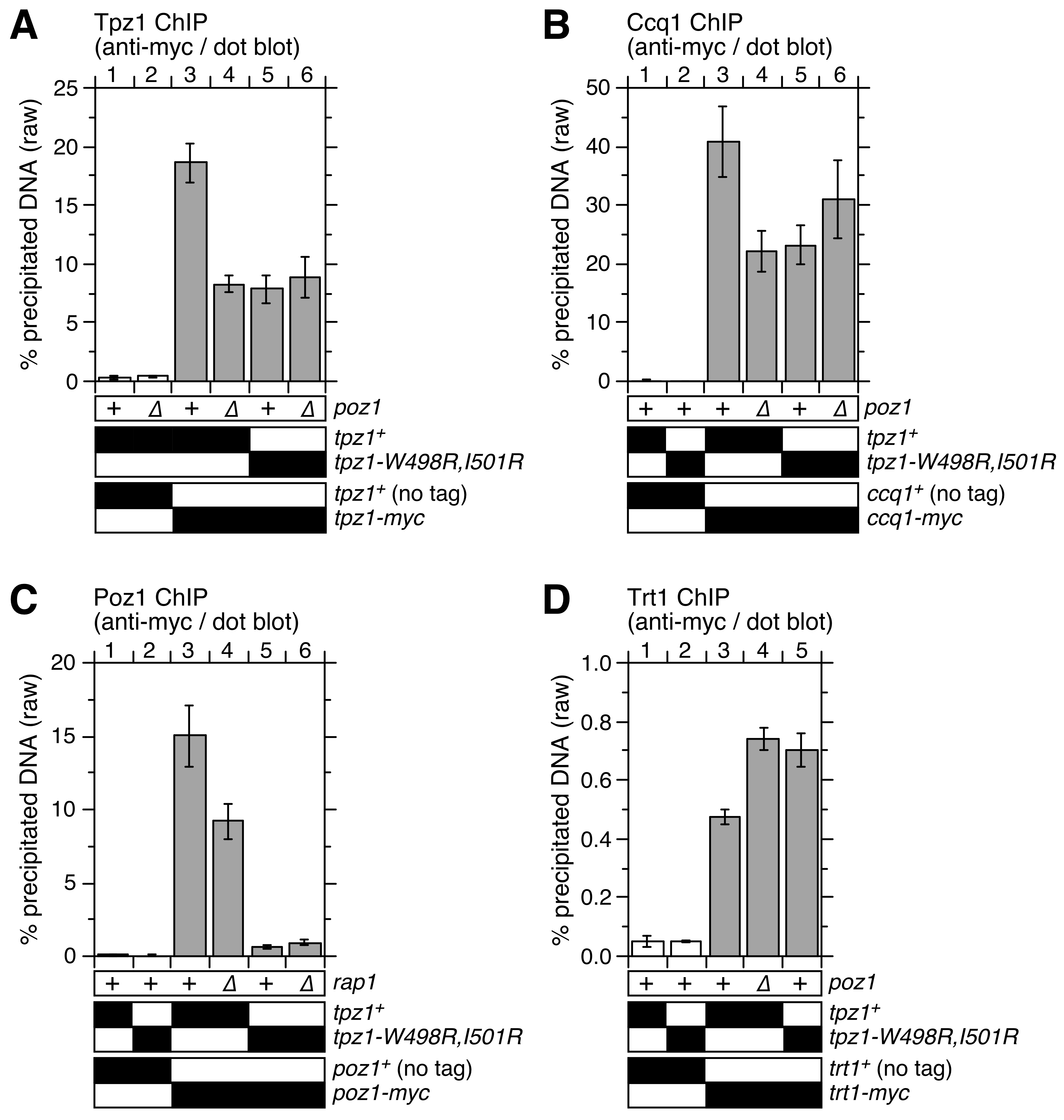

Supplement: Figure S13 — Raw data for Tpz1, Ccq1, Poz1 and Trt1TERT ChIP assays in Tpz1-Poz1 interaction mutant cells. Effects of disrupting Tpz1-Poz1 interaction on telomere association for (A) Tpz1, (B) Ccq1, (C) Poz1 and (D) Trt1TERT were monitored by dot-blot ChIP assays and raw % precipitated DNA values were plotted. These data were then corrected for telomere length [36] to generate plots shown in Figure 7. Error bars represent standard error of the mean from three to eight independent experiments. Statistical analysis of ChIP data by 2-tailed Student's t-test is shown in Table S5. (JPG) [file pgen.1004708.s013.jpg]
