# Supplementary material for: Tpz1-Ccq1 and Tpz1-Poz1 Interactions within Fission Yeast Shelterin Modulate Ccq1 Thr93 Phosphorylation and Telomerase Recruitment
Source: PLoS Genet. 2014 Oct 16;10(10):e1004708. doi: 10.1371/journal.pgen.1004708 (PMC4199508; doi:10.1371/journal.pgen.1004708)
Supplement: Table S1 — Telomere length correction factors (telomere/rDNA). (PDF) [file pgen.1004708.s014.pdf]

Table S1 Telomere length correction factors (telomere/rDNA)

Tagged protein	Genetic background	Correction factor[a]
Tpz1-myc	wt	1.00 ± 0.08
	poz1Δ	4.72 ± 0.29
	tpz1-W498R,I501R	4.71 ± 0.28
	poz1Δ tpz1-W498R,I501R	4.86 ± 0.14
Ccq1-myc	wt	1.00 ± 0.03
	poz1Δ	2.63 ± 0.09
	tpz1-W498R,I501R	2.69 ± 0.08
	poz1Δ tpz1-W498R,I501R	2.63 ± 0.08
Poz1-myc	wt	1.00 ± 0.05
	rap1Δ	3.69 ± 0.14
	tpz1-W498R,I501R	3.78 ± 0.16
	rapΔ tpz1-W498R,I501R	4.01 ± 0.15
Trt1-myc	wt	1.00 ± 0.02
	poz1Δ	3.72 ± 0.14
	tpz1-W498R,I501R	4.90 ± 0.14

[a]Mean ± standard error of the mean. Values are normalized to wild-type cells with indicated tagged proteins.
