# Supplementary material for: Tpz1-Ccq1 and Tpz1-Poz1 Interactions within Fission Yeast Shelterin Modulate Ccq1 Thr93 Phosphorylation and Telomerase Recruitment
Source: PLoS Genet. 2014 Oct 16;10(10):e1004708. doi: 10.1371/journal.pgen.1004708 (PMC4199508; doi:10.1371/journal.pgen.1004708)
Supplement: Table S2 — Fission yeast strains used in this study. (PDF) [file pgen.1004708.s015.pdf]

Table S2 Fission yeast strains used in this study.

Figure		Strain		Full Genotype[a]
3B	1	TN7204	poz1-FLAG	h^{-} his3-D1 poz1^{+}::5FLAG-TEV-Avitag-kanMX6
	2	TN7553	poz1-FLAG tpz1-myc	h^{-} his3-D1 poz1^{+}::5FLAG-TEV-Avitag-kanMX6 tpz1^{+}::13myc-kanMX6
	3	JH11179	poz1-FLAG tpz1-L449R-myc	h^{+} ade6-M216 his3-D1 poz1^{+}::5FLAG-TEV-Avitag-kanMX6 tpz1-L449R::13myc-kanMX6
	4	JH11241	poz1-FLAG tpz1-Y439R,L445R-myc	h^{-} his3-D1 poz1^{+}::5FLAG-TEV-Avitag-kanMX6 tpz1-Y439R,L445R::13myc-kanMX6
	5	JH11271	poz1-FLAG tpz1-Y439R,L445R,L449R-myc	h^{+} his3-D1 poz1^{+}::5FLAG-TEV-Avitag-kanMX6 tpz1-Y439R,L445R,L449R-13myc-kanMX6
3C	1	TN6770	ccq1-FLAG	h^{+} his3-D1 ccq1^{+}::5FLAG-kanMX6
	2	TN7505	ccq1-FLAG tpz1-myc	h^{+} his3-D1 ccq1^{+}::5FLAG-kanMX6 tpz1^{+}::13myc-kanMX6
	3	JH11174	ccq1-FLAG tpz1-L449R-myc	h^{-} ccq1^{+}::5FLAG-kanMX6 tpz1-L449R::13myc-kanMX6
	4	JH11238	ccq1-FLAG tpz1-Y439R,L445R-myc	h^{-} his3-D1 ccq1^{+}::5FLAG-kanMX6 tpz1-Y439R,L445R::13myc-kanMX6
	5	JH11248	ccq1-FLAG tpz1-Y439R,L445R,L449R-myc	h^{+} his3-D1 ccq1^{+}::5FLAG-kanMX6 tpz1-Y439R,L445R,L449R-13myc-kanMX6
3D	1	YTC9716	pot1-FLAG	h^{+} his3-D1 pot1^{+}::3FLAG-kanMX
	2	JH11046	pot1-FLAG tpz1-myc	h^{-} his3-D1 pot1^{+}::3FLAG-kanMX tpz1^{+}::13myc-kanMX6
	3	JH11176	pot1-FLAG tpz1-L449R-myc	h^{+} pot1^{+}::3FLAG-kanMX tpz1-L449R::13myc-kanMX6
	4	JH11244	pot1-FLAG tpz1-Y439R,L445R-myc	h^{+} ade6-M210 his3-D1 pot1^{+}::3FLAG-kanMX tpz1-Y439R,L445R::13myc-kanMX6
	5	JH11273	pot1-FLAG tpz1-Y439R,L445R,L449R-myc	h^{-} pot1^{+}::3FLAG-kanMX tpz1-Y439R,L445R,L449R-13myc-kanMX6
3E	1	TN7553	poz1-FLAG tpz1-myc	h^{-} his3-D1 poz1^{+}::5FLAG-TEV-Avitag-kanMX6 tpz1^{+}::13myc-kanMX6
	2	JH11047	poz1-FLAG tpz1-[1-485]-myc	h^{+} ade6-M210 his3-D1 poz1^{+}::5FLAG-TEV-Avitag-kanMX6 tpz1-[1-485]::13myc-kanMX6
	3	JH11200	poz1-FLAG tpz1-W498R,I501R-myc	h^{+} ade6-M216 his3-D1 poz1^{+}::5FLAG-TEV-Avitag-kanMX6 tpz1-W498R,I501R::13myc-kanMX6
	4	TN7196	tpz1-myc	h^{-} his3-D1 tpz1^{+}::13myc-kanMX6
3F	1	TN6770	ccq1-FLAG	h^{+} his3-D1 ccq1^{+}::5FLAG-kanMX6
	2	TN7505	ccq1-FLAG tpz1-myc	h^{+} his3-D1 ccq1^{+}::5FLAG-kanMX6 tpz1^{+}::13myc-kanMX6
	3	JH10926	ccq1-FLAG tpz1-[1-485]-myc	h^{-} his3-D1 ccq1^{+}::5FLAG-kanMX6 tpz1-[1-485]::13myc-kanMX6
	4	JH11203	ccq1-FLAG tpz1-W498R,I501R-myc	h^{-} his3-D1 ccq1^{+}::5FLAG-kanMX6 tpz1-W498R,I501R::13myc-kanMX6
3G	1	YTC9716	pot1-FLAG	h^{+} his3-D1 pot1^{+}::3FLAG-kanMX
	2	JH11046	pot1-FLAG tpz1-myc	h^{-} his3-D1 pot1^{+}::3FLAG-kanMX tpz1^{+}::13myc-kanMX6
	3	JH10929	pot1-FLAG tpz1-[1-485]-myc	h^{+} his3-D1 pot1^{+}::3FLAG-kanMX tpz1-[1-485]::13myc-kanMX6
	4	JH11196	pot1-FLAG tpz1-W498R,I501R-myc	h^{+} pot1^{+}::3FLAG-kanMX tpz1-W498R,I501R::13myc-kanMX6
4A	1	CF484	tpz1^{+}/tpz1^{+}	h^{+}/h^{-} ade6-M210/ade6-M216 his3-D1/his3-D1
	2,16	TN2411	tpz1^{+}	h^{-} his3-D1
	3	JH11365	tpz1^{+}/tpz1-myc	h^{+}/h^{-} ade6-M210/ade6-M216 his3-D1/his3-D1 tpz1^{+}/tpz1^{+}::13myc-kanMX6
	4,15	TN7196	tpz1-myc	h^{-} his3-D1 tpz1^{+}::13myc-kanMX6
	5	JH11306	tpz1^{+}/tpz1-L449R-myc	h^{+}/h^{-} ade6-M210/ade6-M216 his3^{+}/his3-D1 tpz1^{+}/tpz1-L449R::13myc-kanMX6
	6	JH11835	tpz1-L449R-myc (2x)	h^{+} ade6-M210 his3-D1 tpz1-L449R::13myc-kanMX6 (2x restreaks)
	7	JH12260	tpz1-L449R-myc (5x)	h^{+} ade6-M210 his3-D1 tpz1-L449R::13myc-kanMX6 (5x restreaks)
	8	JH11495	tpz1^{+}/tpz1-Y439R,L445R-myc	h^{+}/h^{-} ade6-M210/ade6-M216 his3^{+}/his3-D1 tpz1^{+}/tpz1-L439R,L445R::13myc-kanMX6
	9	JH11496	tpz1-Y439R,L445R-myc (2x)	h^{-} ade6-M210 his3-D1 tpz1-L439R,L445R::13myc-kanMX6 (2x restreaks)
	10	JH12261	tpz1-Y439R,L445R-myc (5x)	h^{-} ade6-M210 his3-D1 tpz1-L439R,L445R::13myc-kanMX6 (5x restreaks)
	11	JH11500	tpz1^{+}/tpz1-Y439R,L445R,L449R-myc	h^{+}/h^{-} ade6-M210/ade6-M216 his3^{+}/his3-D1 tpz1^{+}/tpz1-L439R,L445R,L449R::13myc-kanMX6
	12	JH11501	tpz1-Y439R,L445R,L449R-myc (2x)	h^{-} ade6-M216 his3-D1 tpz1-L439R,L445R,L449R::13myc-kanMX6 (2x restreaks)
	13	JH12262	tpz1-Y439R,L445R,L449R-myc (5x)	h^{-} ade6-M216 his3-D1 tpz1-L439R,L445R,L449R::13myc-kanMX6 (5x restreaks)
	14	TN9009	tpz1-myc ccq1Δ	h^{+} his3-D1 tpz1-13myc:KanMX6 ccq1Δ::hphMX

Figure		Strain		Full Genotype[a]
4B	1	TN11289	tpz1$^+$	h$^-$ his3-D1 tpz1$^+$::hphMX
	2	CF448	trt1Δ	h$^-$ ade6-M210 his3-D1 trt1Δ::his3$^+$
	3	TN9258	ccq1Δ	h$^-$ his3-D1 ccq1Δ::hphMX (linear)
	4	TN9259	ccq1Δ	h$^-$ his3-D1 ccq1Δ::hphMX (circular)
	5	JH12993	tpz1-L449R	h$^?$ ade6-M210 or M216 his3-D1 tpz1-L449R::hphMX
	6	JH12995	tpz1-L449R	h$^?$ ade6-M210 or M216 his3-D1 tpz1-L449R::hphMX
	7	JH12986	tpz1-Y439R,L445R	h$^?$ ade6-M210 or M216 his3-D1 tpz1-Y439R,L445R::hphMX
	8	JH12988	tpz1-Y439R,L445R	h$^?$ ade6-M210 or M216 his3-D1 tpz1-Y439R,L445R::hphMX
	9	JH12990	tpz1-Y439R,L445R,L449R	h$^?$ ade6-M210 or M216 his3-D1 tpz1-Y439R,L445R,L449R::hphMX
	10	JH12992	tpz1-Y439R,L445R,L449R	h$^?$ ade6-M210 or M216 his3-D1 tpz1-Y439R,L445R,L449R::hphMX
4C	1,7,13	TN2411	tpz1$^+$ ccq1$^+$ poz1$^+$	h$^-$ his3-D1
	2	TN7196	tpz1-myc	h$^-$ his3-D1 tpz1$^+$::13myc-kanMX6
	3	JH12998	tpz1-L449R-myc	h$^-$ ade6-M216 his3-D1 tpz1-L449R::13myc-kanMX6
	4	JH13002	ccq1Δ	h$^-$ his3-D1 ccq1Δ::hphMX
	5	TN9009	ccq1Δ tpz1-myc	h$^+$ his3-D1 ccq1Δ::hphMX tpz1$^+$::13myc-kanMX6
	6	JH11684	ccq1Δ tpz1-L449R-myc	h$^+$ his3-D1 ccq1Δ::hphMX tpz1-L449R-13myc:KanMX6
	8	JH12999	poz1Δ	h$^-$ ade6-M216 his3-D1 poz1Δ::natMX6
	9	TN9254	poz1Δ ccq1Δ	h$^-$ his3-D1 poz1Δ::natMX6 ccq1Δ::hphMX
	10	YTC9366	poz1Δ tpz1-myc	h$^-$ his3-D1 poz1Δ::natMX6 tpz1$^+$::13myc-kanMX6
	11	JH11701	poz1Δ ccq1Δ tpz1-myc	h$^+$ his3-D1 poz1Δ::natMX6 ccq1Δ::hphMX tpz1$^+$::13myc-kanMX6
	12	JH11686	poz1Δ tpz1-L449R-myc	h$^-$ ade6-M216 his3-D1 poz1Δ::natMX6 tpz1-L449R::13myc-kanMX6
4D	1	TN7196	tpz1-myc	h$^-$ his3-D1 tpz1$^+$::13myc-kanMX6
	2	YTC9366	poz1Δ tpz1-myc	h$^-$ his3-D1 poz1Δ::natMX6 tpz1$^+$::13myc-kanMX6
	3	TN9009	ccq1Δ tpz1-myc	h$^+$ his3-D1 ccq1Δ::hphMX tpz1$^+$::13myc-kanMX6
	4	JH11701	poz1Δ ccq1Δ tpz1-myc	h$^+$ his3-D1 poz1Δ::natMX6 ccq1Δ::hphMX tpz1$^+$::13myc-kanMX6
	5	JH11835	tpz1-L449R-myc	h$^+$ ade6-M210 his3-D1 tpz1-L449R::13myc-kanMX6
	6	JH11686	poz1Δ tpz1-L449R-myc	h$^-$ ade6-M216 his3-D1 poz1Δ::natMX6 tpz1-L449R::13myc-kanMX6
	7	JK11683	ccq1Δ tpz1-L449R-myc	h$^+$ his3-D1 ccq1Δ::hphMX tpz1-L449R::13myc-kanMX6
5A	1	TN2411	tpz1$^+$ (no tag)	h$^-$ his3-D1
	2	TN7196	tpz1-myc	h$^-$ his3-D1 tpz1$^+$::13myc-kanMX6
	3	TN9009	tpz1-myc ccq1Δ	h$^+$ his3-D1 tpz1$^+$::13myc-kanMX6 ccq1Δ::hphMX
	4	JH11835	tpz1-L449R-myc	h$^+$ ade6-M210 his3-D1 tpz1-L449R::13myc-kanMX6
	5	JH11496	tpz1-Y439R,L445R-myc	h$^-$ ade6-M210 his3-D1 tpz1-L439R,L445R::13myc-kanMX6
	6	JH11232	tpz1-Y439R,L445R,L449R-myc	h$^+$ ade6-M210 his3-D1 tpz1-Y439R,L445R,L449R::13myc-kanMX6
5B	1	JH11289	ccq1$^+$ (no tag)	h$^-$ his3-D1 tpz1$^+$::hphMX
	2	JH11569	ccq1-myc	h$^+$ his3-D1 ccq1$^+$::13myc-kanMX6 tpz1$^+$::hphMX
	3	JH11622	ccq1-myc tpz1-L449R	h$^+$ his3-D1 ccq1$^+$::13myc-kanMX6 tpz1-L449R::hphMX
	4	JH11452	ccq1-myc tpz1-Y439R,L445R	h$^+$ his3-D1 ccq1$^+$::13myc-kanMX6 tpz1-Y439R,L445R::hphMX
	5	JH11609	ccq1-myc tpz1-Y439R,L445R,L449R	h$^+$ his3-D1 ccq1$^+$::13myc-kanMX6 tpz1-Y439R,L445R,L449R::hphMX
5C	1	JH11289	trt1$^+$ (no tag)	h$^-$ his3-D1 tpz1$^+$::hphMX
	2	JH11630	trt1-myc	h$^-$ ade6-M216 his3-D1 trt1$^+$::G$_8$-13myc-kanMX6 tpz1$^+$::hphMX
	3	JH11626	trt1-myc tpz1-L449R	h$^-$ ade6-M216 his3-D1 trt1$^+$::G$_8$-13myc-kanMX6 tpz1-L449R::hphMX
	4	JH11449	trt1-myc tpz1-Y439R,L445R	h$^+$ ade6-M210 his3-D1 trt1$^+$::G$_8$-13myc-kanMX6 tpz1-Y439R,L445R::hphMX
	5	JH11611	trt1-myc tpz1-Y439R,L445R,L449R	h$^-$ ade6-M210 his3-D1 trt1$^+$::G$_8$-13myc-kanMX6 tpz1-Y439R,L445R,L449R::hphMX
	6	JH11644	trt1-myc ccq1Δ	h$^+$ his3-D1 trt1$^+$::G$_8$-13myc-kanMX6 tpz1$^+$::hphMX ccq1Δ::hphMX
5D	1	TN6770	ccq1-FLAG	h$^+$ his3-D1 ccq1$^+$::5FLAG-kanMX6
	2	TN10988	trt1Δ ccq1-FLAG	h$^-$ ade6-M210 his3-D1 trt1Δ::his3$^+$ ccq1$^+$::5FLAG-kanMX6
	3	TN10991	trt1Δ ccq1-T93A-FLAG	h$^-$ ade6-M210 his3-D1 trt1Δ::his3$^+$ ccq1-T93A::5FLAG-kanMX6
	4	JH11174	ccq1-FLAG tpz1-L449R-myc	h$^-$ ccq1$^+$::5FLAG-kanMX6 tpz1-L449R::13myc-kanMX6
	5	JH11238	ccq1-FLAG tpz1-Y439R,L445R-myc	h$^-$ his3-D1 ccq1$^+$::5FLAG-kanMX6 tpz1-Y439R,L445R::13myc-kanMX6
	6	JH11248	ccq1-FLAG tpz1-Y439R,L445R,L449R-myc	h$^+$ his3-D1 ccq1$^+$::5FLAG-kanMX6 tpz1-Y439R,L445R,L449R-13myc-kanMX6

Figure		Strain		Full Genotype[a]
5E	1	TN6770	ccq1-FLAG	h^+ his3-D1 ccq1$^+$::5FLAG-kanMX6
	2	TN10535	rap1Δ ccq1-FLAG	h^- his3-D1 rap1Δ::ura4$^+$ ccq1$^+$::5FLAG-kanMX6
	3	BAM10829	rap1Δ ccq1-T93A-FLAG	h^- his3-D1 rap1Δ::ura4$^+$ ccq1-T93A::5FLAG-kanMX6
	4	JH11544	rap1Δ tpz1-L449R-myc ccq1-FLAG	h^- his3-D1 rap1Δ::ura4$^+$ tpz1-L449R-13myc-kanMX6 ccq1$^+$::5FLAG-kanMX6
	5	JH11546	rap1Δ tpz1-Y439R,L445R-myc ccq1-FLAG	h^- his3-D1 rap1Δ::ura4$^+$ tpz1-Y439R,L445R-13myc-kanMX6 ccq1$^+$::5FLAG-kanMX6
	6	JH11548	rap1Δ tpz1-Y439R,L445R,L449R-myc ccq1-FLAG	h^+ his3-D1 rap1Δ::ura4$^+$ tpz1-Y439R,L445R,L449R-13myc-kanMX6 ccq1$^+$::5FLAG-kanMX6
6A	1	JH11365	tpz1$^+$/tpz1-myc	h^+/h^- ade6-M210/ade6-M216 his3-D1/his3-D1 tpz1$^+$/tpz1$^+$::13myc-kanMX6
	2	JH11715	tpz1$^+$/tpz1-[1-485]-myc	h^+/h^- ade6-M210/ade6-M216 his3-D1/his3-D1 tpz1$^+$/tpz1-[1-485]::13myc-kanMX6
	3	JH11189	tpz1$^+$/tpz1-W498R,I501R-myc	h^+/h^- ade6-M210/ade6-M216 his3-D1/his3-D1 tpz1$^+$/tpz1-W498R,I501R::13myc-kanMX6
	4	TN2411	tpz1$^+$	h^- his3-D1
	5	TN7196	tpz1-myc	h^- his3-D1 tpz1$^+$::13myc-kanMX6
	6	JH13000	poz1Δ	h^- ade6-M216 his3-D1 poz1Δ::natMX6
	7	YTC9366	tpz1-myc poz1Δ	h^- his3-D1 tpz1$^+$::13myc-kanMX6 poz1Δ::natMX6
	8	JH10923	tpz1-[1-485]-myc	h^- ade6-M210 his3-D1 tpz1-[1-485]::13myc-kanMX6
	9	JH13004	tpz1-W498R,I501R-myc	h^+ ade6-M216 his3-D1 tpz1-W498R,I501R::13myc-kanMX6
	10	JH11693	tpz1-W498R,I501R-myc poz1Δ	h^- ade6-M216 his3-D1 tpz1-W498R,I501R::13myc-kanMX6 poz1Δ::natMX6
	11	JH11689	tpz1-W498R,I501R-myc ccq1Δ	h^- his3-D1 tpz1-W498R,I501R::13myc-kanMX6 ccq1Δ::hphMX
	12	JH11701	tpz1-myc poz1Δ ccq1Δ	h^+ his3-D1 tpz1$^+$::13myc-kanMX6 poz1Δ::natMX6 ccq1Δ::hphMX
6B	1	TN7196	tpz1-myc	h^- his3-D1 tpz1$^+$::13myc-kanMX6
	2	JH11190	tpz1-W498R,I501R-myc	h^+ leu1-32 ura4-D18 ade6-M216 his3-D1 tpz1-W498R,I501R::13myc-kanMX6
	3	JH11692	tpz1-W498R,I501R-myc poz1Δ	h^- leu1-32 ura4-D18 ade6-M216 his3-D1 tpz1-W498R,I501R::13myc-kanMX6 poz1Δ::natMX6
	4	JH11689	tpz1-W498R,I501R-myc ccq1Δ	h^- his3-D1 tpz1-W498R,I501R::13myc-kanMX6 ccq1Δ::hphMX
	5	JH11701	tpz1-myc poz1Δ ccq1Δ	h^+ his3-D1 tpz1$^+$::13myc-kanMX6 poz1Δ::natMX6 ccq1Δ::hphMX
6C	1,8	TN2411	tpz1$^+$	h^- his3-D1
	2	TN7196	tpz1-myc	h^- his3-D1 tpz1$^+$::13myc-kanMX6
	3	JH11835	tpz1-L449R-myc	h^+ ade6-M210 his3-D1 tpz1-L449R::13myc-kanMX6
	4	JH10922	tpz1-[1-485]-myc	h^- ade6-M210 his3-D1 tpz1-[1-485]::13myc-kanMX6
	5	JH11790	tpz1-[1-485]-L449R-myc	h^- ade6-M216 his3-D1 tpz1-[1-485]-L449R::13myc-kanMX6
	6	JH11190	tpz1-W498R,I501R-myc	h^+ ade6-M216 his3-D1 tpz1-W498R,I501R::13myc-kanMX6
	7	JH11781	tpz1-L449R,W498R,I501R-myc	h^+ ade6-M216 his3-D1 tpz1-L449R,W498R,I501R::13myc-kanMX6
6D	1	JH11835	tpz1-L449R-myc	h^+ ade6-M210 his3-D1 tpz1-L449R::13myc-kanMX6
	2	JH10922	tpz1-[1-485]-myc	h^- ade6-M210 his3-D1 tpz1-[1-485]::13myc-kanMX6
	3	JH11790	tpz1-[1-485]-L449R-myc	h^- ade6-M216 his3-D1 tpz1-[1-485]-L449R::13myc-kanMX6
	4	JH11190	tpz1-W498R,I501R-myc	h^+ ade6-M216 his3-D1 tpz1-W498R,I501R::13myc-kanMX6
	5	JH11781	tpz1-L449R,W498R,I501R-myc	h^+ ade6-M216 his3-D1 tpz1-L449R,W498R,I501R::13myc-kanMX6
7A	1	TN7196	tpz1-myc	h^- his3-D1 tpz1$^+$::13myc-kanMX6
	2	YTC9366	tpz1-myc poz1Δ	h^- his3-D1 tpz1$^+$::13myc-kanMX6 poz1Δ::natMX6
	3	JH11191	tpz1-W498R,I501R-myc	h^+ ade6-M216 his3-D1 tpz1-W498R,501R::13myc-kanMX6
	4	JH11692	tpz1-W498R,I501R-myc poz1Δ	h^- ade6-M216 his3-D1 tpz1-W498R,I501R::13myc-kanMX6 poz1Δ::natMX6
7B	1	TN7217	ccq1-myc	h^- his3-D1 ccq1$^+$::13myc-kanMX6
	2	YTC8712	ccq1-myc poz1Δ	h^+ his3-D1 ccq1$^+$::13myc-kanMX6 poz1Δ::natMX6
	3	JH12895	ccq1-myc tpz1-W498R,I501R	h^+ his3-D1 ccq1$^+$::13myc-kanMX6 tpz1-W498R,I501R::hphMX
	4	JH12897	ccq1-myc tpz1-W498R,I501R poz1Δ	h^- his3-D1 ccq1$^+$::13myc-kanMX6 tpz1-W498R,I501R::hphMX poz1Δ::natMX6

Figure		Strain		Full Genotype[a]
7C	1	JH12265	poz1-myc	h^{+} his3-D1 poz1^{+}::13myc-kanMX6 tpz1^{+}::hphMX
	2	JH12267	poz1-myc rap1Δ	h^{+} his3-D1 poz1^{+}::13myc-kanMX6 tpz1^{+}::hphMX rap1Δ::ura4^{+}
	3	JH12659	poz1-myc tpz1-W498R,I501R	h^{+} ade6-M210 his3-D1 poz1^{+}::13myc-kanMX6 tpz1-W498R,I501R::hphMX
	4	JH12056	poz1-myc tpz1-W498R,I501R rap1Δ	h^{+} ade6-M210 his3-D1 poz1^{+}::13myc-kanMX6 tpz1-W498R,I501R::hphMX rap1Δ::ura4^{+}
7D	1	JH11630	trt1-myc	h^{-} ade6-M216 his3-D1 trt1^{+}::G_8-13myc-kanMX6 tpz1^{+}::hphMX
	2	JH12770	trt1-myc poz1Δ	h^{-} ade6-M216 his3-D1 trt1^{+}::G_8-13myc-kanMX6 tpz1^{+}::hphMX poz1Δ::natMX6
	3	JH12766	trt1-myc tpz1-W498R,I501R	h^{-} his3-D1 trt1^{+}::G_8-13myc-kanMX6 tpz1-W498R,I501R::hphMX
7E	1	TN6770	ccq1-FLAG	h^{+} his3-D1 ccq1^{+}::5FLAG-kanMX6
	2	TN10531	ccq1-FLAG poz1Δ	h^{-} his3-D1 ccq1^{+}::5FLAG-kanMX6 poz1Δ::natMX6
	3	TN12036	ccq1-T93A-FLAG poz1Δ	h^{-} his3-D1 ccq1-T93A::5FLAG-kanMX6 poz1Δ::natMX6
	4	JH10926	ccq1-FLAG tpz1-[1-485]-myc	h^{-} his3-D1 ccq1^{+}::5FLAG-kanMX6 tpz1-[1-485]::13myc-kanMX6
	5	JH11203	ccq1-FLAG tpz1-W498R,I501R-myc	h^{-} his3-D1 ccq1^{+}::5FLAG-kanMX6 tpz1-W498R,I501R::13myc-kanMX6
	6	JH11772	ccq1-FLAG tpz1-W498R,I501R-myc poz1Δ	h^{+} ade6-M216 his3-D1 ccq1^{+}::5FLAG-kanMX6 tpz1-W498R,I501R::13myc-kanMX6 poz1Δ::natMX6
S3A	1,5	TN6770	ccq1-FLAG	h^{+} his3-D1 ccq1^{+}::5FLAG-kanMX6
	2,6	TN7505	ccq1-FLAG tpz1-myc	h^{+} his3-D1 ccq1^{+}::5FLAG-kanMX6 tpz1^{+}::13myc-kanMX6
	3,7	JH11174	ccq1-FLAG tpz1-L449R-myc	h^{-} ccq1^{+}::5FLAG-kanMX6 tpz1-L449R::13myc-kanMX6
	4,8	JH12780	ccq1-FLAG tpz1-L449A-myc	h^{-} his3-D1 ccq1^{+}::5FLAG-kanMX6 tpz1-L449A::13myc-kanMX6
S3B	1	TN14536	tpz1^{+}/tpz1-L449A	h^{+}/h^{-} ade6-M210/ade6-M216 his3-D1/his3-D1 tpz1^{+}/tpz1-L449A::hphMX
	2	TN14539	tpz1-L449A (2x)	h^{-} ade6-M216 his3-D1 tpz1-L449A::hphMX (2x restreaks)
	3	TN14577	tpz1-L449A (5x)	h^{-} ade6-M216 his3-D1 tpz1-L449A::hphMX (5x restreaks)
	4	TN14540	tpz1-L449A (2x)	h^{-} ade6-M216 his3-D1 tpz1-L449A::hphMX (2x restreaks)
	5	TN14579	tpz1-L449A (5x)	h^{-} ade6-M216 his3-D1 tpz1-L449A::hphMX (5x restreaks)
	6	TN14541	tpz1-L449A (2x)	h^{-} ade6-M216 his3-D1 tpz1-L449A::hphMX (2x restreaks)
	7	TN14581	tpz1-L449A (5x)	h^{-} ade6-M216 his3-D1 tpz1-L449A::hphMX (5x restreaks)
	8	TN14542	tpz1-L449A (2x)	h^{-} ade6-M216 his3-D1 tpz1-L449A::hphMX (2x restreaks)
	9	TN14583	tpz1-L449A (5x)	h^{-} ade6-M216 his3-D1 tpz1-L449A::hphMX (5x restreaks)
	10	TN11289	tpz1^{+}	h^{-} his3-D1 tpz1^{+}::hphMX
S3C	1	TN11289	tpz1^{+}	h^{-} his3-D1 tpz1^{+}::hphMX
	2	TN14578	tpz1-L449A	h^{-} ade6-M216 his3-D1 tpz1-L449A::hphMX
	3	TN14580	tpz1-L449A	h^{-} ade6-M216 his3-D1 tpz1-L449A::hphMX
	4	TN14582	tpz1-L449A	h^{-} ade6-M216 his3-D1 tpz1-L449A::hphMX
	5	TN14584	tpz1-L449A	h^{-} ade6-M216 his3-D1 tpz1-L449A::hphMX
S3D	1,12	TN11289	tpz1^{+}	h^{-} his3-D1 tpz1^{+}::hphMX
	2	JH12999	poz1Δ	h^{-} ade6-M216 his3-D1 poz1Δ::natMX6
	3	TN14539	tpz1-L449A	h^{-} ade6-M216 his3-D1 tpz1-L449A::hphMX
	4	TN14540	tpz1-L449A	h^{-} ade6-M216 his3-D1 tpz1-L449A::hphMX
	5	TN14574	tpz1-L449A poz1Δ	h^{-} ade6-M216 his3-D1 tpz1-L449A::hphMX poz1Δ::natMX6
	6	TN14575	tpz1-L449A poz1Δ	h^{+} his3-D1 tpz1-L449A::hphMX poz1Δ::natMX6
	7	TN14576	tpz1-L449A poz1Δ	h^{-} ade6-M216 his3-D1 tpz1-L449A::hphMX poz1Δ::natMX6
	8	JH11577	tpz1-L449R	h^{+} ade6-M216 his3-D1 tpz1-L449R::hphMX
	9	TN14444	tpz1-L449R poz1Δ	h^{-} his3-D1 tpz1-L449R::hphMX poz1Δ::natMX6
	10	TN14445	tpz1-L449R poz1Δ	h^{+} ade6-M216 his3-D1 tpz1-L449R::hphMX poz1Δ::natMX6
	11	TN14446	tpz1-L449R poz1Δ	h^{+} ade6-M216 his3-D1 tpz1-L449R::hphMX poz1Δ::natMX6
S3E	1	TN11289	tpz1^{+}	h^{-} his3-D1 tpz1^{+}::hphMX
	2	TN14539	tpz1-L449A	h^{-} ade6-M216 his3-D1 tpz1-L449A::hphMX
	3	TN14540	tpz1-L449A	h^{-} ade6-M216 his3-D1 tpz1-L449A::hphMX
	4	TN14574	tpz1-L449A poz1Δ	h^{-} ade6-M216 his3-D1 tpz1-L449A::hphMX poz1Δ::natMX6
	5	TN14575	tpz1-L449A poz1Δ	h^{+} his3-D1 tpz1-L449A::hphMX poz1Δ::natMX6
	6	TN14576	tpz1-L449A poz1Δ	h^{-} ade6-M216 his3-D1 tpz1-L449A::hphMX poz1Δ::natMX6
	7	JH11577	tpz1-L449R	h^{+} ade6-M216 his3-D1 tpz1-L449R::hphMX
	8	TN14444	tpz1-L449R poz1Δ	h^{-} his3-D1 tpz1-L449R::hphMX poz1Δ::natMX6
	9	TN14445	tpz1-L449R poz1Δ	h^{+} ade6-M216 his3-D1 tpz1-L449R::hphMX poz1Δ::natMX6
	10	TN14446	tpz1-L449R poz1Δ	h^{+} ade6-M216 his3-D1 tpz1-L449R::hphMX poz1Δ::natMX6

Figure		Strain		Full Genotype^{a}
S4	1	TN2411	tpz1$^+$	h^- his3-D1
	2,14,15	TN7196	tpz1-myc	h^- his3-D1 tpz1$^+$::13myc-kanMX6
	3-6	JH11181	tpz1-L449R-myc	h^- ade6-M216 tpz1-L449R::13myc-kanMX6 (multiple 11x restreak single colonies derived from JH11181)
	7,16,23	LK8667	ccq1Δ	h^- his3-D1 ccq1Δ::hphMX
	8-13	JH11218	tpz1-Y439R,L445R-myc	h^+ ade6-M210 his3-D1 tpz1-Y439R,L445R::13myc-kanMX6 (multiple 11x restreak single colonies derived from JH11218)
	17-22	JH11232	tpz1-Y439R,L445R,L449R-myc	h^+ ade6-M210 his3-D1 tpz1-Y439R,L445R,L449R::13myc-kanMX6 (multiple 11x restreak single colonies derived from JH11232)
S5		CF248	trt1$^+$/trt1Δ	h^+/h^- ade6-M210/ade6-M216 his3-D1/his3-D1 trt1$^+$/trt1Δ::his3$^+$
		JH12706	trt1$^+$/trt1Δ ccq1$^+$/ccq1Δ	h^+/h^- ade6-M210/ade6-M216 his3-D1/his3-D1 trt1$^+$/trt1Δ::his3$^+$ ccq1$^+$/ccq1Δ::hphMX
		JH12263	trt1$^+$/trt1Δ tpz1$^+$/tpz1-L449R	h^+/h^- ade6-M210/ade6-M216 his3-D1/his3-D1 trt1$^+$/trt1Δ::his3$^+$ tpz1$^+$/tpz1-L449R::hphMX
S6A		CF199	wt	h^- ade6-M210 his3-D1
		TN6584	ccq1Δ	h^- ade6-M210 his3-D1 ccq1Δ::hphMX
		JH11578	tpz1-L449R	h^- ade6-M216 his3-D1 tpz1-L449R::hphMX
		JH11423	tpz1-Y439R,L445R	h^- ade6-M210 his3-D1 tpz1-Y439R,L445R::hphMX
		JH11574	tpz1-Y439R,L445R,L449R	h^- ade6-M210 his3-D1 tpz1-Y439R,L445R,L449R::hphMX
S6B	1,2	JH11722	chk1-myc	h^- ade6-M216 his3-D1 chk1$^+$::9myc-2HA-6his-ura4$^+$ tpz1$^+$::hphMX
	3	JH11724	chk1-myc ccq1Δ	h^+ his3-D1 chk1$^+$::9myc-2HA-6his-ura4$^+$ tpz1$^+$::hphMX ccq1Δ::hphMX
	4	JH11636	chk1-myc tpz1-L449R	h^- ade6-M216 his3-D1 chk1$^+$::9myc-2HA-6his-ura4$^+$ tpz1-L449R::hphMX
	5	JH11638	chk1-myc tpz1-Y439R,L445R	h^- ade6-M216 his3-D1 chk1$^+$::9myc-2HA-6his-ura4$^+$ tpz1-Y439R,L445R::hphMX
	6	JH11640	chk1-myc tpz1-Y439R,L445R,L449R	h^+ ade6-M216 his3-D1 chk1$^+$::9myc-2HA-6his-ura4$^+$ tpz1-Y439R,L445R,L449R::hphMX
S7A	1	TN2411	his$^-$	h^- his3-D1
	2	TN3784	his$^+$	h^-
	3	CF52	telomere (1L)::his3$^+$	h^{90} ura4-DS/E ade6-M210 his3-D1 his3$^+$::tel(1L)
	4	TN9133	telomere (1L)::his3$^+$ ccq1Δ	h^- his3-D1 ccq1Δ::hphMX his3$^+$::tel(1L)
	5	TN9630	telomere (1L)::his3$^+$ tpz1-L449R	h^+ ade6-M216 his3-D1 tpz1-L449R::13myc-kanMX6 his3$^+$::tel(1L)
	6	TN9623	telomere (1L)::his3$^+$ tpz1-Y439R,L445R	h^- ade6-M210 his3-D1 tpz1-Y439R,L445R::13myc-kanMX6 his3$^+$::tel(1L)
S7B	1	TN2411	his$^-$	h^- his3-D1
	2	TN3784	his$^+$	h^-
	3	CF52	telomere (1L)::his3$^+$	h^{90} ura4-DS/E ade6-M210 his3-D1 his3$^+$::tel(1L)
	4	TN9607	telomere (1L)::his3$^+$ poz1Δ	h^+ ade6-M210 his3-D1 poz1Δ::natMX6 his3$^+$::tel(1L)
	5	TN9614	telomere (1L)::his3$^+$ tpz1-W498R,I501R	h^+ ade6-M210 his3-D1 tpz1-W498R,I501R::13myc-kanMX6 his3$^+$::tel(1L)
	6	TN9618	telomere (1L)::his3$^+$ tpz1-[1-485]	h^+ ade6-M210 his3-D1 tpz1-[1-485]::13myc-kanMX6 his3$^+$::tel(1L)
S8A	1	TN6770	ccq1-FLAG	h^+ his3-D1 ccq1$^+$::5FLAG-kanMX6
	2	TN10988	trt1Δ ccq1-FLAG	h^- ade6-M210 his3-D1 trt1Δ::his3$^+$ ccq1$^+$::5FLAG-kanMX6
	3	TN10991	trt1Δ ccq1-T93A-FLAG	h^- ade6-M210 his3-D1 trt1Δ::his3$^+$ ccq1-T93A::5FLAG-kanMX6
	4	JH11174	ccq1-FLAG tpz1-L449R-myc	h^- ccq1$^+$::5FLAG-kanMX6 tpz1-L449R::13myc-kanMX6
	5	JH11238	ccq1-FLAG tpz1-Y439R,L445R-myc	h^- his3-D1 ccq1$^+$::5FLAG-kanMX6 tpz1-Y439R,L445R::13myc-kanMX6
	6	JH11248	ccq1-FLAG tpz1-Y439R,L445R,L449R-myc	h^+ his3-D1 ccq1$^+$::5FLAG-kanMX6 tpz1-Y439R,L445R,L449R-13myc-kanMX6
S8B	1	JH11289	ccq1$^+$ (no tag)	h^- his3-D1 tpz1$^+$::hphMX
	2	JH11569	ccq1-myc	h^+ his3-D1 ccq1$^+$::13myc-kanMX6 tpz1$^+$::hphMX
	3	JH11622	ccq1-myc tpz1-L449R	h^+ his3-D1 ccq1$^+$::13myc-kanMX6 tpz1-L449R::hphMX
	4	JH11452	ccq1-myc tpz1-Y439R,L445R	h^+ his3-D1 ccq1$^+$::13myc-kanMX6 tpz1-Y439R,L445R::hphMX
	5	JH11609	ccq1-myc tpz1-Y439R,L445R,L449R	h^+ his3-D1 ccq1$^+$::13myc-kanMX6 tpz1-Y439R,L445R,L449R::hphMX

Figure		Strain		Full Genotype[a]
S8C	1	JH11289	$trt1^+$ (no tag)	h^- his3-D1 $tpz1^+$::hphMX
	2	JH11630	trt1-myc	h^- ade6-M216 his3-D1 $trt1^+$::G_8-13myc-kanMX6 $tpz1^+$::hphMX
	3	JH11626	trt1-myc tpz1-L449R	h^- ade6-M216 his3-D1 $trt1^+$::G_8-13myc-kanMX6 tpz1-L449R::hphMX
	4	JH11449	trt1-myc tpz1-Y439R,L445R	h^+ ade6-M210 his3-D1 $trt1^+$::G_8-13myc-kanMX6 tpz1-Y439R,L445R::hphMX
	5	JH11611	trt1-myc tpz1-Y439R,L445R,L449R	h^- ade6-M210 his3-D1 $trt1^+$::G_8-13myc-kanMX6 tpz1-Y439R,L445R,L449R::hphMX
	6	JH11644	trt1-myc ccq1Δ	h^+ his3-D1 $trt1^+$::G_8-13myc-kanMX6 $tpz1^+$::hphMX ccq1Δ::hphMX
S8D	1	TN2411	$tpz1^+$	h^- his3-D1
	2	TN7196	tpz1-myc	h^- his3-D1 $tpz1^+$::13myc-kanMX6
	3	TN9009	tpz1-myc ccq1Δ	h^+ his3-D1 tpz1-13myc:KanMX6 ccq1Δ::hphMX
	4	JH11181	tpz1-L449R-myc	h^- ade6-M216 tpz1-L449R::13myc-kanMX6
	5	JH11218	tpz1-Y439R,L445R-myc	h^+ ade6-M210 his3-D1 tpz1-Y439R,L445R::13myc-kanMX6
	6	JH11232	tpz1-Y439R,L445R,L449R-myc	h^+ ade6-M210 his3-D1 tpz1-Y439R,L445R,L449R::13myc-kanMX6
S9A	1	JH11289	$ccq1^+$ (no tag)	h^- his3-D1 $tpz1^+$::hphMX
	2	JH11569	ccq1-myc	h^+ his3-D1 $ccq1^+$::13myc-kanMX6 $tpz1^+$::hphMX
	3	TN14558	ccq1-myc tpz1-L449A	h^- his3-D1 $ccq1^+$::13myc-kanMX6 tpz1-L449A::hphMX
	4	JH11622	ccq1-myc tpz1-L449R	h^+ his3-D1 $ccq1^+$::13myc-kanMX6 tpz1-L449R::hphMX
S9B	1	JH11289	$trt1^+$ (no tag)	h^- his3-D1 $tpz1^+$::hphMX
	2	JH11630	trt1-myc	h^- ade6-M216 his3-D1 $trt1^+$::G_8-13myc-kanMX6 $tpz1^+$::hphMX
	3	JH11644	trt1-myc ccq1Δ	h^+ his3-D1 $trt1^+$::G_8-13myc-kanMX6 $tpz1^+$::hphMX ccq1Δ::hphMX
	4	TN14545	trt1-myc tpz1-L449A	h^- his3-D1 $trt1^+$::G_8-13myc-kanMX6 tpz1-L449A::hphMX
	5	JH11626	trt1-myc tpz1-L449R	h^- ade6-M216 his3-D1 $trt1^+$::G_8-13myc-kanMX6 tpz1-L449R::hphMX
	6	YTC12756	trt1-D743A-myc	h^- ade6-M210 his3-D1 trt1-D743A::G_8-13myc-kanMX6
S10B	1	TN2411	$tpz1^+$ (no tag)	h^- his3-D1
	2	TN7196	tpz1-myc	h^- his3-D1 $tpz1^+$::13myc-kanMX6
	3	JH10915	tpz1-[1-379]-myc	h^- ade6-M216 tpz1-[1-379]::13myc-kanMX6
	4	JH10919	tpz1-[1-420]-myc	h^- ade6-M216 his3-D1 tpz1-[1-420]::13myc-kanMX6
	5	JH11215	tpz1-[Δ421-485]-myc	h^+ ade6-M216 his3-D1 tpz1-[Δ421-485]::13myc-kanMX6
	6	JH10922	tpz1-[1-485]-myc	h^- ade6-M210 his3-D1 tpz1-[1-485]::13myc-kanMX6
S10C	1,7	TN2411	$tpz1^+$ (no tag)	h^- his3-D1
	2	TN7196	tpz1-myc	h^- his3-D1 $tpz1^+$::13myc-kanMX6
	3	JH10915	tpz1-[1-379]-myc	h^- ade6-M216 tpz1-[1-379]::13myc-kanMX6
	4	JH10919	tpz1-[1-420]-myc	h^- ade6-M216 his3-D1 tpz1-[1-420]::13myc-kanMX6
	5	JH11215	tpz1-[Δ421-485]-myc	h^+ ade6-M216 his3-D1 tpz1-[Δ421-485]::13myc-kanMX6
	6	JH10922	tpz1-[1-485]-myc	h^- ade6-M210 his3-D1 tpz1-[1-485]::13myc-kanMX6
S10D	1	TN7196	tpz1-myc	h^- his3-D1 $tpz1^+$::13myc-kanMX6
	2	JH10915	tpz1-[1-379]-myc	h^- ade6-M216 tpz1-[1-379]::13myc-kanMX6
	3	JH10919	tpz1-[1-420]-myc	h^- ade6-M216 his3-D1 tpz1-[1-420]::13myc-kanMX6
	4	JH11215	tpz1-[Δ421-485]-myc	h^+ ade6-M216 his3-D1 tpz1-[Δ421-485]::13myc-kanMX6
	5	JH10922	tpz1-[1-485]-myc	h^- ade6-M210 his3-D1 tpz1-[1-485]::13myc-kanMX6
S11B		TN7196	tpz1-myc	h^- his3-D1 $tpz1^+$::13myc-kanMX6
		JH12981	tpz1-myc pot1Δ	h^- ade6-M216 his3-D1 $tpz1^+$::13myc-kanMX6 pot1Δ::natMX
S11C	1	TN7196	tpz1-myc	h^- his3-D1 $tpz1^+$::13myc-kanMX6
	2	YTC9366	tpz1-myc poz1Δ	h^- his3-D1 $tpz1^+$::13myc-kanMX6 poz1Δ::natMX6
	3	TN9009	tpz1-myc ccq1Δ	h^+ his3-D1 tpz1-13myc:KanMX6 ccq1Δ::hphMX
	4	JH11701	tpz1-myc poz1Δ ccq1Δ	h^+ his3-D1 $tpz1^+$::13myc-kanMX6 poz1Δ::natMX6 ccq1Δ::hphMX
	5	JH11835	tpz1-L449R-myc	h^+ ade6-M210 his3-D1 tpz1-L449R::13myc-kanMX6
	6	JH11190	tpz1-W498R,I501R-myc	h^+ leu1-32 ura4-D18 ade6-M216 his3-D1 tpz1-W498R,I501R::13myc-kanMX6
	7	JH11781	tpz1-L449R,W498R,I501R-myc	h^+ ade6-M216 his3-D1 tpz1-L449R,W498R,I501R::13myc-kanMX6
	8	JH10922	tpz1-[1-485]-myc	h^- ade6-M210 his3-D1 tpz1-[1-485]::13myc-kanMX6
	9	JH11790	tpz1-[1-485]-L449R-myc	h^- ade6-M216 his3-D1 tpz1-[1-485]-L449R::13myc-kanMX6

Figure		Strain		Full Genotype[a]
S11D	1	YTC9716	pot1-FLAG	h^+ his3-D1 pot1$^+$::3FLAG-kanMX
	2	JH11046	pot1-FLAG tpz1-myc	h^- his3-D1 pot1$^+$::3FLAG-kanMX tpz1$^+$::13myc-kanMX6
	3	JH12971	pot1-FLAG tpz1-myc ccq1Δ	h^- his3-D1 pot1$^+$::3FLAG-kanMX tpz1$^+$::13myc-kanMX6 ccq1Δ::hphMX
	4	JH12975	pot1-FLAG tpz1-myc poz1Δ	h^+ his3-D1 pot1$^+$::3FLAG-kanMX tpz1$^+$::13myc-kanMX6 poz1Δ::natMX6
	5	JH12977	pot1-FLAG tpz1-myc poz1Δ ccq1Δ	h^+ his3-D1 pot1$^+$::3FLAG-kanMX tpz1$^+$::13myc-kanMX6 poz1Δ::natMX6 ccq1Δ::hphMX
S12A	1	TN2411	tpz1$^+$	h^- his3-D1
	2	YTC8354	poz1Δ	h^- ade6-M216 his3-D1 poz1Δ::natMX6
	3	TN5345	rap1Δ	h^+ his3-D1 rap1Δ::ura4$^+$
	4	TN7196	tpz1-myc	h^- his3-D1 tpz1$^+$::13myc-kanMX6
	5	YTC9366	tpz1-myc poz1Δ	h^- his3-D1 tpz1$^+$::13myc-kanMX6 poz1Δ::natMX6
	6	YTC9310	tpz1-myc rap1Δ	h^- his3-D1 tpz1$^+$::13myc-kanMX6 rap1Δ::ura4$^+$
	7	JH12951	tpz1-myc poz1Δ rap1Δ	h^- his3-D1 tpz1$^+$::13myc-kanMX6 poz1Δ::natMX6 rap1Δ::ura4$^+$
	8	JH11191	tpz1-W498R,I501R-myc	h^+ ade6-M216 his3-D1 tpz1-W498R,I501R::13myc-kanMX6
	9	JH11692	tpz1-W498R,I501R-myc poz1Δ	h^- ade6-M216 his3-D1 tpz1-W498R,I501R::13myc-kanMX6 poz1Δ::natMX6
	10	JH12052	tpz1-W498R,I501R-myc rap1Δ	h^+ ade6-M216 his3-D1 tpz1-W498R,I501R::13myc-kanMX6 rap1Δ::ura4$^+$
	11	JH12955	tpz1-W498R,I501R-myc poz1Δ rap1Δ	h^- his3-D1 tpz1-W498R,I501R::13myc-kanMX6 poz1Δ::natMX6 rap1Δ::ura4$^+$
S12B	1	TN2411	tpz1$^+$	h^- his3-D1
	2	JH12908	tpz1-W498R,I501R	h^- ade6-M210 his3-D1 tpz1-W498R,I501R::hphMX
	3	TN7217	ccq1-myc	h^- his3-D1 ccq1$^+$::13myc-kanMX6
	4	YTC8712	ccq1-myc poz1Δ	h^+ his3-D1 ccq1$^+$::13myc-kanMX6 poz1Δ::natMX6
	5	JH12894	ccq1-myc tpz1-W498R,I501R	h^- his3-D1 ccq1$^+$::13myc-kanMX6 tpz1-W498R,I501R::hphMX
	6	JH12897	ccq1-myc tpz1-W498R,I501R poz1Δ	h^- his3-D1 ccq1$^+$::13myc-kanMX6 tpz1-W498R,I501R::hphMX poz1Δ::natMX6
S12C	1	TN11289	tpz1$^+$	h^- his3-D1 tpz1$^+$::hphMX
	2	JH12908	tpz1-W498R,I501R	h^- ade6-M210 his3-D1 tpz1-W498R,I501R::hphMX
	3	JH12265	poz1-myc	h^+ his3-D1 poz1$^+$::13myc-kanMX6 tpz1$^+$::hphMX
	4	JH12267	poz1-myc rap1Δ	h^+ his3-D1 poz1$^+$::13myc-kanMX6 tpz1$^+$::hphMX rap1Δ::ura4$^+$
	5	JH12659	poz1-myc tpz1-W498R,I501R	h^+ ade6-M210 his3-D1 poz1$^+$::13myc-kanMX6 tpz1-W498R,I501R::hphMX
	6	JH12056	poz1-myc tpz1-W498R,I501R rap1Δ	h^+ ade6-M210 his3-D1 poz1$^+$::13myc-kanMX6 tpz1-W498R,I501R::hphMX rap1Δ::ura4$^+$
S12D	1	TN11289	tpz1$^+$	h^- his3-D1 tpz1$^+$::hphMX
	2	JH12908	tpz1-W498R,I501R	h^- ade6-M210 his3-D1 tpz1-W498R,I501R::hphMX
	3	JH11630	trt1-myc	h^- ade6-M216 his3-D1 trt1$^+$::G_8-13myc-kanMX6 tpz1$^+$::hphMX
	4	JH12770	trt1-myc poz1Δ	h^- ade6-M216 his3-D1 trt1$^+$::G_8-13myc-kanMX6 tpz1$^+$::hphMX poz1Δ::natMX6
	5	JH12766	trt1-myc tpz1-W498R,I501R	h^- his3-D1 trt1$^+$::G_8-13myc-kanMX6 tpz1-W498R,I501R::hphMX
S13A	1	TN2411	tpz1$^+$	h^- his3-D1
	2	YTC8354	poz1Δ	h^- ade6-M216 his3-D1 poz1Δ::natMX6
	3	TN7196	tpz1-myc	h^- his3-D1 tpz1$^+$::13myc-kanMX6
	4	YTC9366	tpz1-myc poz1Δ	h^- his3-D1 tpz1$^+$::13myc-kanMX6 poz1Δ::natMX6
	5	JH11191	tpz1-W498R,I501R-myc	h^+ ade6-M216 his3-D1 tpz1-W498R,501R::13myc-kanMX6
	6	JH11692	tpz1-W498R,I501R-myc poz1Δ	h^- ade6-M216 his3-D1 tpz1-W498R,I501R::13myc-kanMX6 poz1Δ::natMX6
S13B	1	TN2411	tpz1$^+$	h^- his3-D1
	2	JH12908	tpz1-W498R,I501R	h^- ade6-M210 his3-D1 tpz1-W498R,I501R::hphMX
	3	TN7217	ccq1-myc	h^- his3-D1 ccq1$^+$::13myc-kanMX6
	4	YTC8712	ccq1-myc poz1Δ	h^+ his3-D1 ccq1$^+$::13myc-kanMX6 poz1Δ::natMX6
	5	JH12895	ccq1-myc tpz1-W498R,I501R	h^+ his3-D1 ccq1$^+$::13myc-kanMX6 tpz1-W498R,I501R::hphMX
	6	JH12897	ccq1-myc tpz1-W498R,I501R poz1Δ	h^- his3-D1 ccq1$^+$::13myc-kanMX6 tpz1-W498R,I501R::hphMX poz1Δ::natMX6

Figure		Strain		Full Genotype[a]
S13C	1	TN11289	*tpz1*$^{+}$	*h*$^{-}$ *his3-D1 tpz1*$^{+}$*::hphMX*
	2	JH12908	*tpz1-W498R,I501R*	*h*$^{-}$ *ade6-M210 his3-D1 tpz1-W498R,I501R::hphMX*
	3	JH12265	*poz1-myc*	*h*$^{+}$ *his3-D1 poz1*$^{+}$*::13myc-kanMX6 tpz1*$^{+}$*::hphMX*
	4	JH12267	*poz1-myc rap1Δ*	*h*$^{+}$ *his3-D1 poz1*$^{+}$*::13myc-kanMX6 tpz1*$^{+}$*::hphMX rap1Δ::ura4*$^{+}$
	5	JH12659	*poz1-myc tpz1-W498R,I501R*	*h*$^{+}$ *ade6-M210 his3-D1 poz1*$^{+}$*::13myc-kanMX6 tpz1-W498R,I501R::hphMX*
	6	JH12056	*poz1-myc tpz1-W498R,I501R rap1Δ*	*h*$^{+}$ *ade6-M210 his3-D1 poz1*$^{+}$*::13myc-kanMX6 tpz1-W498R,I501R::hphMX rap1Δ::ura4*$^{+}$
S13D	1	TN11289	*tpz1*$^{+}$	*h*$^{-}$ *his3-D1 tpz1*$^{+}$*::hphMX*
	2	JH12908	*tpz1-W498R,I501R*	*h*$^{-}$ *ade6-M210 his3-D1 tpz1-W498R,I501R::hphMX*
	3	JH11630	*trt1-myc*	*h*$^{-}$ *ade6-M216 his3-D1 trt1*$^{+}$*::*G_8*-13myc-kanMX6 tpz1*$^{+}$*::hphMX*
	4	JH12770	*trt1-myc poz1Δ*	*h*$^{-}$ *ade6-M216 his3-D1 trt1*$^{+}$*::*G_8*-13myc-kanMX6 tpz1*$^{+}$*::hphMX poz1Δ::natMX6*
	5	JH12766	*trt1-myc tpz1-W498R,I501R*	*h*$^{-}$ *his3-D1 trt1*$^{+}$*::*G_8*-13myc-kanMX6 tpz1-W498R,I501R::hphMX*

[1]All strains are *leu1-32 ura4-D18* (haploid) or *leu1-32/leu1-32 ura4-D18/ura4-D18* (diploid)
