# Supplementary material for: Tpz1-Ccq1 and Tpz1-Poz1 Interactions within Fission Yeast Shelterin Modulate Ccq1 Thr93 Phosphorylation and Telomerase Recruitment
Source: PLoS Genet. 2014 Oct 16;10(10):e1004708. doi: 10.1371/journal.pgen.1004708 (PMC4199508; doi:10.1371/journal.pgen.1004708)
Supplement: Table S3 — Plasmids used to integrate tpz1 mutant alleles into fission yeast. (PDF) [file pgen.1004708.s016.pdf]

Table S3 Plasmids used to integrate *tpz1* mutant alleles into fission yeast.

Plasmid (Lab stock #)	Mutant alleles
pBS-tpz1-13myc-kanMX (607)	*tpz1-myc*
pBS-tpz1-L449R-13myc-kanMX (697)	*tpz1-L449R-myc*
pBS-tpz1-L449A-13myc-kanMX (836)	*tpz1-L449A-myc*
pBS-tpz1-Y439R,L445R-13myc-kanMX (716)	*tpz1-Y439R,L445R-myc*
pBS-tpz1-Y439R,L445R,L449R-13myc-kanMX (718)	*tpz1-Y439R,L445R,L449R-myc*
pBS-tpz1(1-485)-13myc-kanMX (654)	*tpz1-[1-485]-myc*
pBS-tpz1-W498R,I501R-13myc-kanMX (707)	*tpz1-W498R,I501R-myc*
pBS-tpz1(1-485)-L449R-13myc-kanMX (773)	*tpz1-[1-485]-L449R-myc*
pBS-tpz1-L449R,W498R,I501R-13myc-kanMX (772)	*tpz1-L449R,W498R,I501R-myc*
pBS-tpz1(1-379)-13myc-kanMX (652)	*tpz1-[1-379]-myc*
pBS-tpz1(1-420)-13myc-kanMX (653)	*tpz1-[1-420]-myc*
pBS-tpz1(Δ421-485)-13myc-kanMX (670)	*tpz1-[Δ421-485]-myc*
