# Supplementary material for: Tpz1-Ccq1 and Tpz1-Poz1 Interactions within Fission Yeast Shelterin Modulate Ccq1 Thr93 Phosphorylation and Telomerase Recruitment
Source: PLoS Genet. 2014 Oct 16;10(10):e1004708. doi: 10.1371/journal.pgen.1004708 (PMC4199508; doi:10.1371/journal.pgen.1004708)
Supplement: Table S4 — Plasmids used in yeast 2-hybrid assays. (PDF) [file pgen.1004708.s017.pdf]

Table S4 Plasmids used in yeast 2-hybrid assays.

Plasmid (Lab stock #)	Description in Figures
pGADT7 (352)	vector
pGADT7-Poz1 (452)	Poz1
pGAD-GH-Ccq1 (549)	Ccq1
pTM623 (pMP623; Pot1 with GAD) (464)[1]	Pot1
pGBKT7 (356)	vector
pGBKT7-Tpz1 (495)	Tpz1-FL (full length)
pGBKT7-Tpz1-[379-508] (614)	Tpz1-[379-508]
pGBKT7-Tpz1-[421-508] (616)	Tpz1-[421-508]
pGBKT7-Tpz1-[421-485] (651)	Tpz1-[421-485]
pGBKT7-Tpz1-[486-508] (650)	Tpz1-[486-508]
pGBKT7-Tpz1-[1-485] (708)	Tpz1-[1-485]
pGBKT7-Tpz1-[Δ421-485] (689)	Tpz1-[Δ421-485]
pGBKT7-Tpz1-Y439R (682)	Y439R
pGBKT7-Tpz1-L445R (683)	L445R
pGBKT7-Tpz1-E446R (684)	E446R
pGBKT7-Tpz1-Y439R,L445R,E446R (666)	Y439R,L445R,E446R
pGBKT7-Tpz1-Y439R,L445R (700)	Y439R,L445R
pGBKT7-Tpz1-L449R,Y453R (667)	L449R,Y453R
pGBKT7-Tpz1-L449R (685)	L449R
pGBKT7-Tpz1-Y439R,L445R,L449R (701)	Y439R,L445R,L449R
pGBKT7-Tpz1-Y453R (686)	Y453R
pGBKT7-Tpz1-[421-485]-L449R (763)	Tpz1-[421-485] L449R
pGBKT7-Tpz1-[421-485]-Y439R,L445R (762)	Tpz1-[421-485] Y439R,L445R
pGBKT7-Tpz1-[421-485]-Y439R,L445R,L449R (764)	Tpz1-[421-485] Y439R,L445R,L449R
pGBKT7-Tpz1-W498R,I501R (669)	W498R,I501R
pGBKT7-Tpz1-[486-508]-W498R,I501R (673)	Tpz1-[486-508] W498R,I501R
pGBKT7-Tpz1-K386A (594)	K386A
pGBKT7-Tpz1-R433A,K434A (597)	R433A,K434A
pGBKT7-Tpz1-E455A,K459A,K460A (598)	E455A,K459A,K460A
pGBKT7-Tpz1-D497A,K500A,R505A (600)	D497A,K500A,R505A
pGBKT7-Tpz1-R433A,K434A,E455A,K459A,K460A (617)	R433A,K434A,E455A,K459A,K460A
pGBKT7-Tpz1-R433A,K434A,D497A,K500A,R505A (619)	R433A,K434A,D497A,K500A,R505A
pGBKT7-Tpz1-E455A,K459A,K460A,D497A,K500A,R505A (620)	E455A,K459A,K460A,D497A,K500A,R505A
pGBKT7-Tpz1-R433E,K434E (643)	R433E,K434E
pGBKT7-Tpz1-K459E,K460E (644)	K459E,K460E
pGBKT7-Tpz1-K500E,R505E (645)	K500E,R505E
pGBKT7-Tpz1-W498A,I501A (794)	W498A,I501A
pGBKT7-Tpz1-L449A (795)	L449A
pGBKT7-Tpz1-Y439A,L445A (796)	Y439A,L445A

[1]From Ishikawa lab.
