# Supplementary material for: Tpz1-Ccq1 and Tpz1-Poz1 Interactions within Fission Yeast Shelterin Modulate Ccq1 Thr93 Phosphorylation and Telomerase Recruitment
Source: PLoS Genet. 2014 Oct 16;10(10):e1004708. doi: 10.1371/journal.pgen.1004708 (PMC4199508; doi:10.1371/journal.pgen.1004708)
Supplement: Table S5 — Statistical analysis of ChIP and TER1 co-IP data by 2-tailed Student's t-test. (PDF) [file pgen.1004708.s018.pdf]

Table S5 Statistical analysis of ChIP and *TER1* co-IP data by 2-tailed Student's t-test.

Figure	Genotypes and p-values[1]					
5A		no tag	*tpz1-myc*	*tpz1-myc ccq1Δ*	*tpz1-L449R -myc*	*tpz1-Y439R, L445R-myc*
	tpz1-myc	2.5x10^{-4}				
	tpz1-myc ccq1Δ	4.9x10^{-5}	0.024			
	tpz1-L449R-myc	1.9x10^{-4}	9.3x10^{-3}	0.22		
	tpz1-Y439R,L445R-myc	8.9x10^{-3}	7.6x10^{-3}	0.11	0.41	
	tpz1-Y439R,L445R,L449R-myc	0.018	0.029	0.46	0.98	0.61
5B		no tag	*ccq1-myc*	*ccq1-myc tpz1-L449R*	*ccq1-myc tpz1-Y439R, L445R*	
	ccq1-myc	4.6x10^{-7}				
	ccq1-myc tpz1-L449R	5.6x10^{-6}	1.5x10^{-4}			
	ccq1-myc tpz1-Y439R,L445R	2.3x10^{-5}	7.3x10^{-5}	0.037		
	ccq1-myc tpz1-Y439R,L445R,L449R	2.1x10^{-5}	2.0x10^{-4}	0.29	0.21	
5C		no tag	*trt1-myc*	*trt1-myc ccq1Δ*	*trt1-myc tpz1-L449R*	*trt1-myc tpz1-Y439R, L445R*
	trt1-myc	4.9x10^{-6}				
	trt1-myc ccq1Δ	8.2x10^{-3}	4.1x10^{-3}			
	trt1-myc tpz1-L449R	1.7x10^{-5}	3.2x10^{-4}	0.47		
	trt1-myc tpz1-Y439R,L445R	2.7x10^{-3}	5.1x10^{-3}	0.82	0.67	
	trt1-myc tpz1-Y439R,L445R,L449R	8.3x10^{-3}	4.4x10^{-3}	0.96	0.53	0.87
7A		*tpz1-myc*	*tpz1-myc poz1Δ*	*tpz1-W498R, I501R-myc*		
	tpz1-myc poz1Δ	2.2x10^{-3}				
	tpz1-W498R,I501R-myc	1.1x10^{-3}	0.65			
	tpz1-W498R,I501R-myc poz1Δ	7.6x10^{-3}	0.55	0.41		
7B		*ccq1-myc*	*ccq1-myc poz1Δ*	*ccq1-myc tpz1-498R, I501R*		
	ccq1-myc poz1Δ	0.39				
	ccq1-myc tpz1-W498R,I501R	0.10	0.77			
	ccq1-myc tpz1-W498R,I501R poz1Δ	0.07	0.28	0.35		
7C		*poz1-myc*	*poz1-myc rap1Δ*	*poz1-myc tpz1-498R, I501R*		
	poz1-myc rap1Δ	7.7x10^{-3}				
	poz1-myc tpz1-W498R,I501R	3.9x10^{-3}	1.6x10^{-3}			
	poz1-myc tpz1-W498R,I501R rap1Δ	1.8x10^{-3}	4.3x10^{-4}	0.21		

7D		*trt1-myc*	*trt1-myc poz1Δ*			
	trt1-myc poz1Δ	$5.3x10^{-6}$				
	trt1-myc tpz1-W498R,I501R	$6.0x10^{-5}$	0.09			

S8D		no tag	*tpz1-myc*	*tpz1-myc ccq1Δ*	*tpz1-L449R -myc*	*tpz1-Y439R, L445R-myc*
	tpz1-myc	$1.1x10^{-3}$				
	tpz1-myc ccq1Δ	0.46	$2.0x10^{-4}$			
	tpz1-L449R-myc	0.80	$1.3x10^{-4}$	0.19		
	tpz1-Y439R,L445R-myc	0.79	$4.8x10^{-4}$	0.50	0.52	
	tpz1-Y439R,L445R,L449R-myc	0.54	$2.0x10^{-4}$	0.22	0.24	0.64

S9A		no tag	*ccq1-myc*	*ccq1-myc tpz1-L449A*		
	ccq1-myc	$2.7x10^{-3}$				
	ccq1-myc tpz1-L449A	$8.2x10^{-3}$	0.056			
	ccq1-myc tpz1-L449R	$6.4x10^{-6}$	0.035	0.66		

S9B		no tag	*trt1-myc*	*trt1-myc ccq1Δ*	*trt1-myc tpz1-L449A*	*rt1-myc tpz1-L449R*
	trt1-myc	$4.9x10^{-6}$				
	trt1-myc ccq1Δ	$8.2x10^{-3}$	$4.1x10^{-3}$			
	trg1-myc tpz1-L449A	$1.1x10^{-5}$	$1.2x10^{-2}$	0.15		
	trt1-myc tpz1-L449R	$1.7x10^{-5}$	$3.2x10^{-4}$	0.47	0.20	
	trt1-D743A-myc	$8.1x10^{-9}$	$6.9x10^{-3}$	$7.3x10^{-5}$	$4.8x10^{-5}$	$1.1x10^{-7}$

S13A		no tag (*tpz1*$^{+}$)	*poz1Δ* (no tag)	*tpz1-myc*	*tpz1-myc poz1Δ*	*tpz1-W498R, I501R-myc*
	poz1Δ (no tag)	0.54				
	tpz1-myc	$1.8x10^{-9}$	$2.8x10^{-5}$			
	tpz1-myc poz1Δ	$5.2x10^{-9}$	$3.1x10^{-5}$	$1.0x10^{-3}$		
	tpz1-W498R,I501R-myc	$7.9x10^{-10}$	$5.1x10^{-6}$	$2.5x10^{-3}$	0.67	
	tpz1-W498R,I501R-myc poz1Δ	$1.9x10^{-7}$	$3.2x10^{-4}$	$2.6x10^{-3}$	0.66	0.50

S13B		no tag (*tpz1*$^{+}$)	*tpz1-W498R, I501R* (no tag)	*ccq1-myc*	*ccq1-myc poz1Δ*	*ccq1-myc tpz1-498R, I501R*
	tpz1-W498R,I501R (no tag)	$7.2x10^{-2}$				
	ccq1-myc	$1.1x10^{-4}$	$1.1x10^{-4}$			
	ccq1-myc poz1Δ	$2.1x10^{-4}$	$2.1x10^{-4}$	0.037		
	ccq1-myc tpz1-W498R,I501R	$1.0x10^{-4}$	$1.0x10^{-4}$	0.041	0.85	
	ccq1-myc tpz1-W498R,I501R poz1Δ	$1.0x10^{-3}$	$1.0x10^{-3}$	0.31	0.28	0.32

S13C		no tag (*tpz1*$^{+}$)	*tpz1-W498R, I501R* (no tag)	*poz1-myc*	*poz1-myc rap1Δ*	*poz1-myc tpz1-498R, I501R*
	tpz1-W498R,I501R (no tag)	0.44				
	poz1-myc	1.7×10^{-3}	7.6×10^{-5}			
	poz1-myc rap1Δ	1.2×10^{-3}	4.3×10^{-5}	0.048		
	poz1-myc tpz1-W498R,I501R	0.010	6.2×10^{-4}	2.1×10^{-3}	1.6×10^{-3}	
	poz1-myc tpz1-W498R,I501R rap1Δ	4.5×10^{-3}	2.6×10^{-4}	5.0×10^{-4}	4.1×10^{-4}	0.27

S13D		no tag (*tpz1*$^{+}$)	*tpz1-W498R, I501R* (no tag)	*trt1-myc*	*trt1-myc poz1Δ*	
	tpz1-W498R,I501R (no tag)	0.97				
	trt1-myc	9.8×10^{-6}	3.6×10^{-6}			
	trt1-myc poz1Δ	2.9×10^{-6}	1.7×10^{-6}	1.1×10^{-3}		
	trt1-myc tpz1-W498R,I501R	3.3×10^{-5}	2.6×10^{-5}	0.011	0.61	

[1]Statistically significant differences ($p \leq 0.05$) are highlighted with red letters.
