# Supplementary material for: Tpz1-Ccq1 and Tpz1-Poz1 Interactions within Fission Yeast Shelterin Modulate Ccq1 Thr93 Phosphorylation and Telomerase Recruitment
Source: PLoS Genet. 2014 Oct 16;10(10):e1004708. doi: 10.1371/journal.pgen.1004708 (PMC4199508; doi:10.1371/journal.pgen.1004708)
Supplement: Supporting Information S1 — A single PDF file containing all Supporting Information (Figures S1, S2, S3, S4, S5, S6, S7, S8, S9, S10, S11, S12, S13 and Tables S1, S2, S3, S4, S5). (PDF) [file pgen.1004708.s019.pdf]

Supporting Information S1

Tpz1-Ccq1 and Tpz1-Poz1 interactions within fission yeast shelterin modulate Ccq1 Thr93 phosphorylation and telomerase recruitment

Jennifer L. Harland[1,*], Ya-Ting Chang[1,*], Bettina A. Moser[1] and Toru M. Nakamura[1,‡]

[1]Department of Biochemistry and Molecular Genetics, College of Medicine, University of Illinois at Chicago, Chicago, Illinois 60607

[*]These authors contributed equally to this work.
[‡]Corresponding author: nakamut@uic.edu

Figures S1-S13

Tables S1-S5

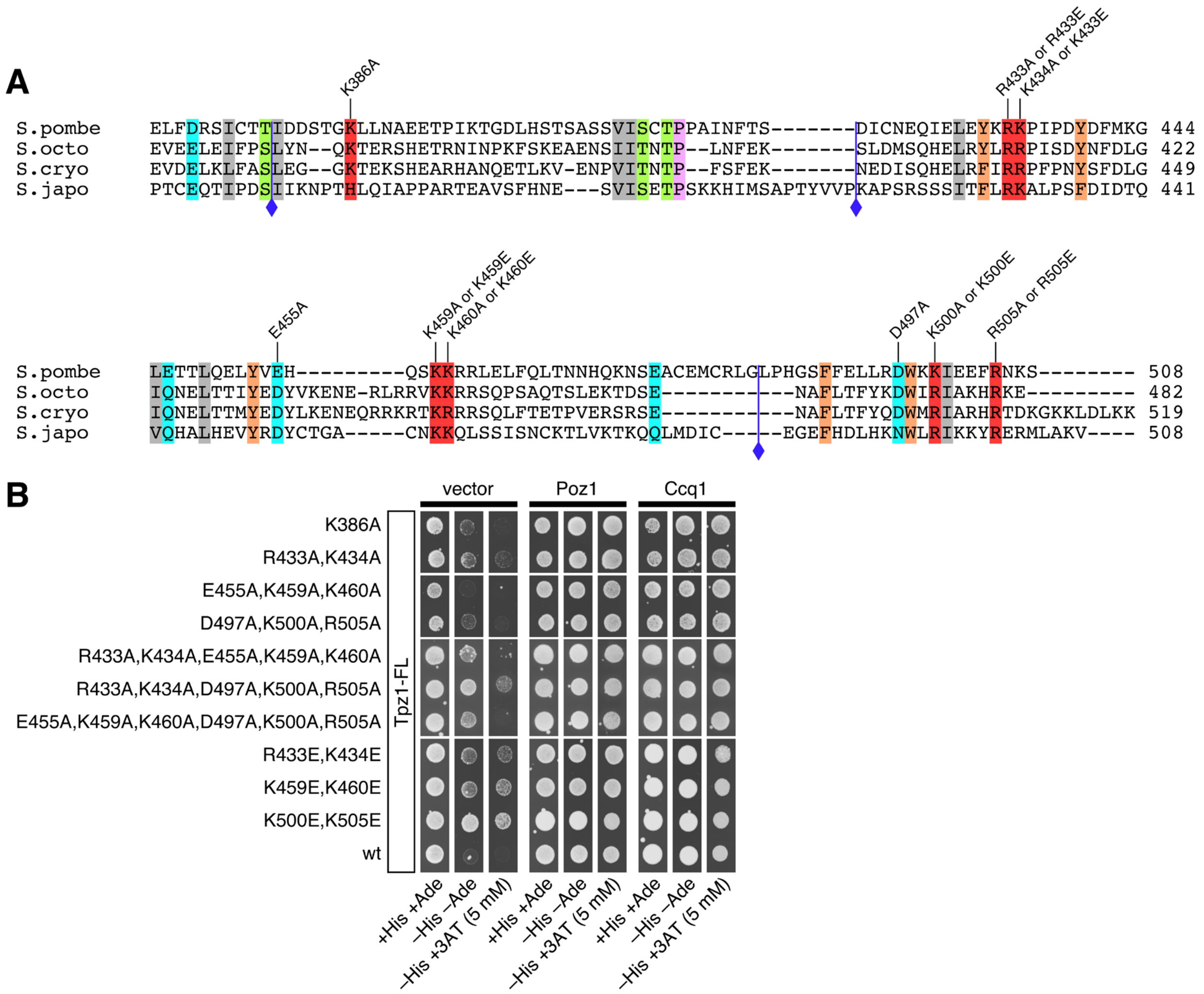

Figure S1. Y2H mutational analysis of charged residues within Tpz1-Ccq1 and Tpz1-Poz1 interaction domains.

(**A**) Sequence alignment of Tpz1 regions responsible for Tpz1-Ccq1 and Tpz1-Poz1 interaction in *S. pombe* and corresponding regions from three additional *Schizosaccharomyces* species (*S. octosporus*, *S. cryophilus* and *S. japonicus*). Conserved charged amino acids mutated for Y2H analysis are indicated. Blue diamonds indicate various truncation mutants of Tpz1. (See Figure 1C.) (**B**) Y2H assay for mutations that did not affect Tpz1-Ccq1 or Tpz1-Poz1 interaction. Indicated mutations were tested in full length Tpz1 (Tpz1-FL). For charged residues (Lys, Arg, Asp and Glu), either a charge swap (Lys/Arg to Glu) or an Alanine mutation (Lys/Arg/Asp/Glu to Ala) was introduced. Positive interactions were identified by growth on –His –Ade and –His +5mM 3AT plates.

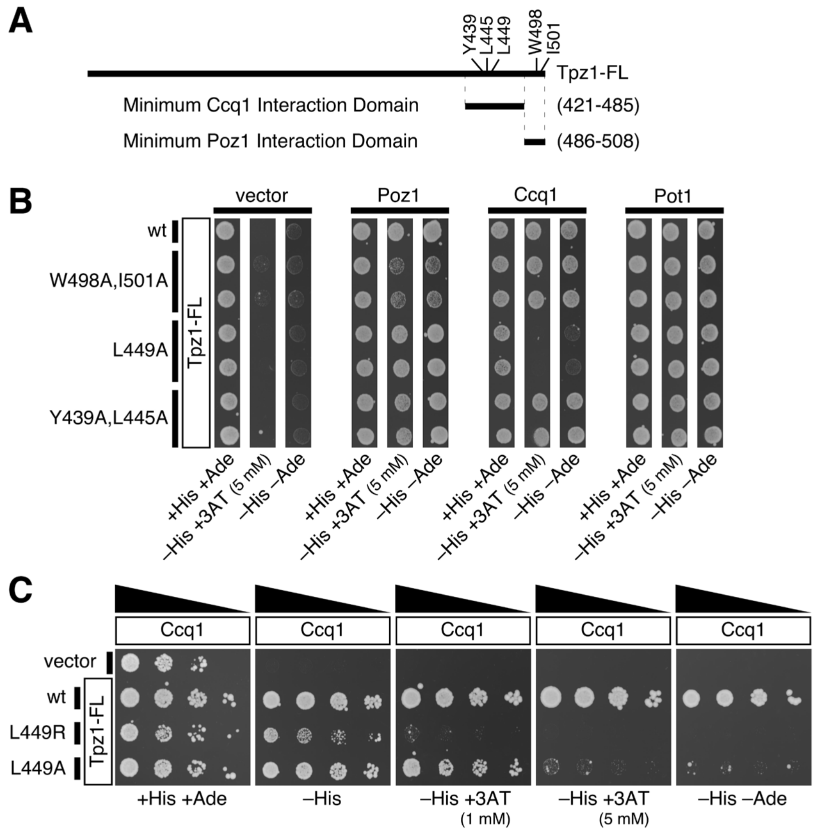

Figure S2. Y2H analysis of hydrophobic to alanine mutations within Tpz1-Ccq1 and Tpz1-Poz1 interaction domains.

(**A**) A schematic representation of Tpz1, marked with Ccq1 and Poz1 interaction domains. (**B**) Y2H assay for alanine mutations of conserved hydrophobic residues within Tpz1-Ccq1 or Tpz1-Poz1 interaction domains. Indicated mutations were tested in full length Tpz1 (Tpz1-FL). Positive interactions were identified by growth on –His –Ade and –His +5mM 3AT plates. (**C**) Y2H assay comparison of Ccq1-Tpz1 interaction for wild-type (wt) Tpz1, Tpz1-L449R and Tpz1-L449A. 5-fold serial dilution series were spotted on indicated plates. For all selection plates tested, L449A showed a milder defect on Tpz1-Ccq1 Y2H interaction than L449R.

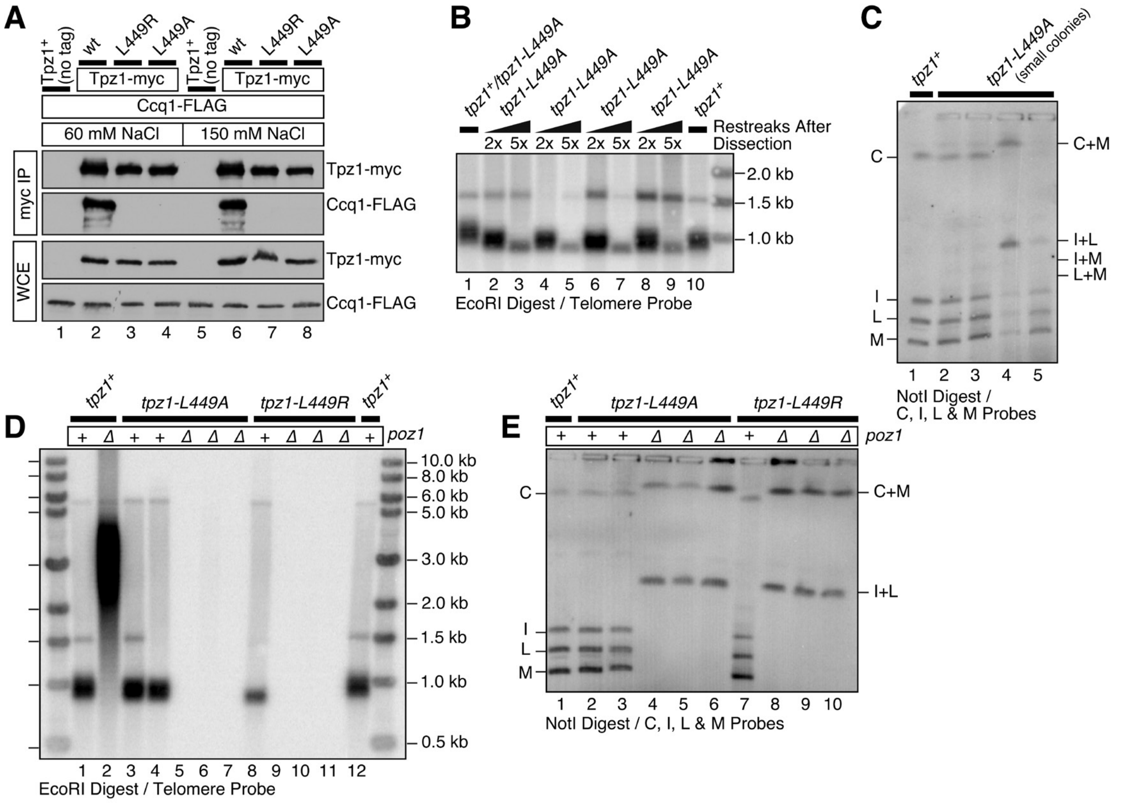

Figure S3. Comparison of *tpz1-L449R* and *tpz1-L449A* mutant alleles.

(**A**) Examination of Tpz1-Ccq1 interaction by co-IP in less stringent (60 mM NaCl) or our standard (150 mM NaCl) lysis/wash conditions. We found that L449R and L449A are equally effective in disrupting Tpz1-Ccq1 interaction detected in co-IP experiments. (**B**) Southern blot analysis of telomere length for *tpz1-L449A* cells. Haploid cells were generated by dissection of spores derived from heterozygous *tpz1*$^{+}$*/*mutated *tpz1* diploid cells, and restreaked twice or 5 times on plates (estimated to be 40-50 or 100-125 cell divisions, respectively) prior to preparation of genomic DNA. For each round of restreak, several faster growing colonies were combined and streaked for single colonies on YES plates. (**C**) Pulsed-field gel analysis of telomere fusions for early generation small colonies of *tpz1-L449A* cells, which showed I+L fusion band as well as much fainter bands for I+M, L+M, I, L and M bands for two out of four clones tested. (See Figure 4B for a NotI-restriction site map of fission yeast chromosomes.) Compared to the *tpz1-L449R* mutant shown in Figure 4B, tpz1-*L449A* showed less prominent early telomere fusions. (**D-E**) Epistasis analysis for telomere loss by Southern blot (D) or telomere fusion by pulsed-field gel (E) indicated that both *L449A* and *L449R* alleles show synergistic telomere loss and fusion phenotypes in *poz1Δ* cells. Samples were prepared from early generation cells after strains were generated by genetic cross of parental haploid strains and dissection of resulting double mutant spores.

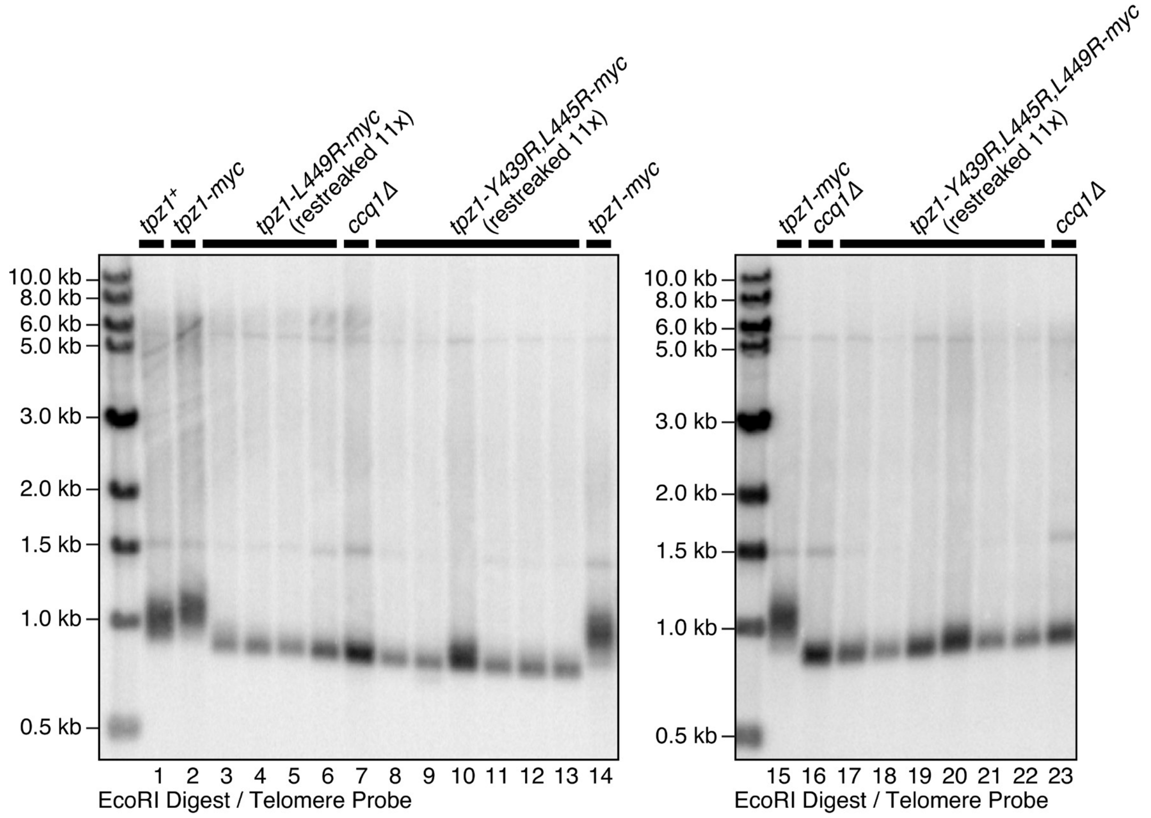

Figure S4. Effects of Tpz1-Ccq1 interaction disruption mutations on telomere maintenance.

Southern blot analysis of telomere length for indicated Tpz1-Ccq1 interaction disruption mutants. Multiple independent haploid cells were generated by dissection of spores derived from heterozygous *tpz1*$^+$/mutated *tpz1* diploid cells, and restreaked 11 times (estimated to be 220-275 cell divisions) on plates prior to preparation of genomic DNA. For each round of restreak, several faster growing colonies were combined and streaked for single colonies on YES plates.

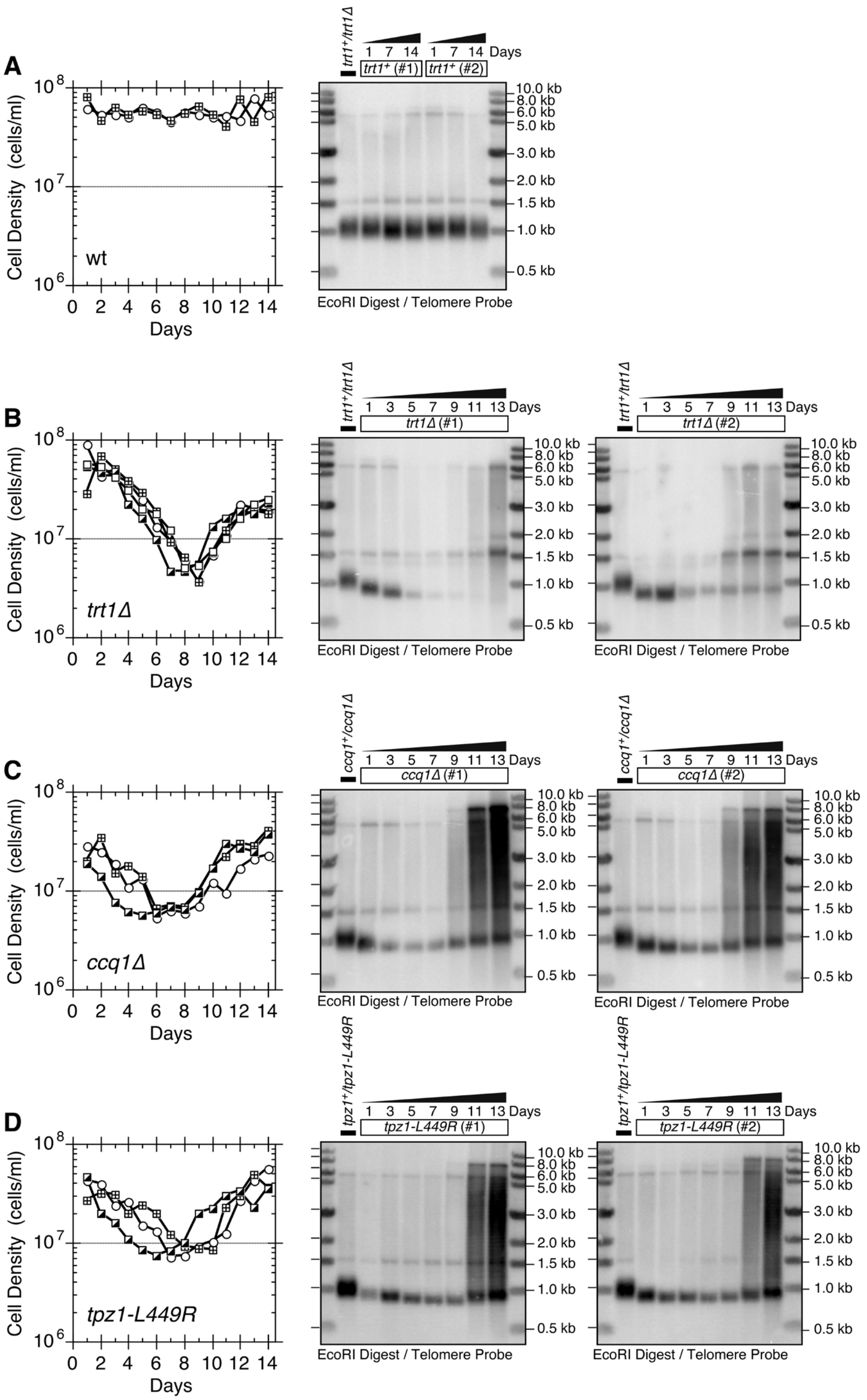

Figure S5. Liquid culture growth assay and Southern blot telomere analysis of Tpz1-Ccq1 interaction disruption mutant L449R.

Wild-type (wt) (**A**), *trt1*Δ (**B**), *ccq1*Δ (**C**) and *tpz1-L449R* (**D**) haploid colonies (freshly derived by dissection of spores derived from heterozygous diploid cells) were inoculated and subsequently serially diluted to $4x10^4$ cells/ml every 24 hours in fresh YES. Cell densities were counted after 24 hours and plotted (left panels). For Southern blot analysis (right panels), cells from two cultures for each genotype were harvested and genomic DNA was prepared for days indicated.

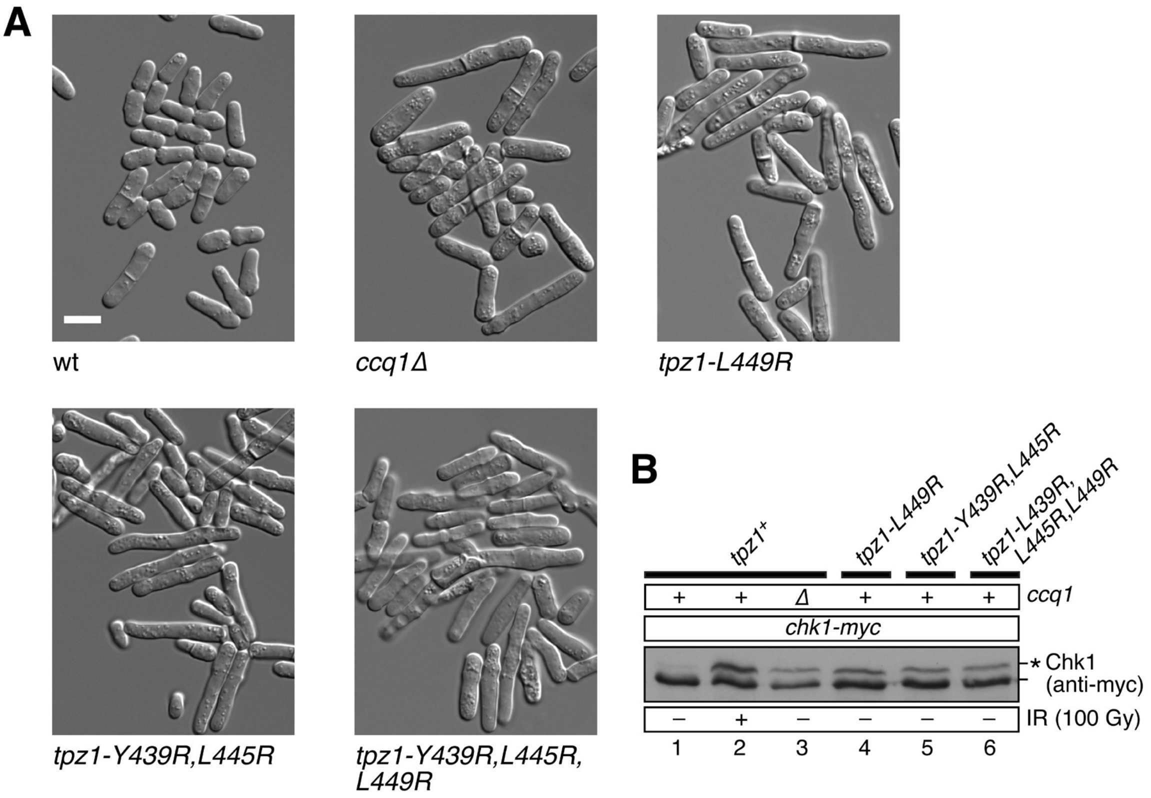

Figure S6. Tpz1-Ccq1 interaction is necessary to prevent checkpoint activation.

(**A**) Microscopic analysis of early generation (20-40 cell divisions) wild-type (wt), *ccq1*Δ and *tpz1*-mutant cells grown in liquid YES culture. White scale bar indicates 10 μm. (**B**) Western blot analysis of whole cell extracts from wild-type, *ccq1*Δ and *tpz1*-mutant cells expressing Chk1-myc. Wild-type cells were also exposed to 100 Gy of gamma-irradiation to induce DNA damage and Chk1 phosphorylation (*).

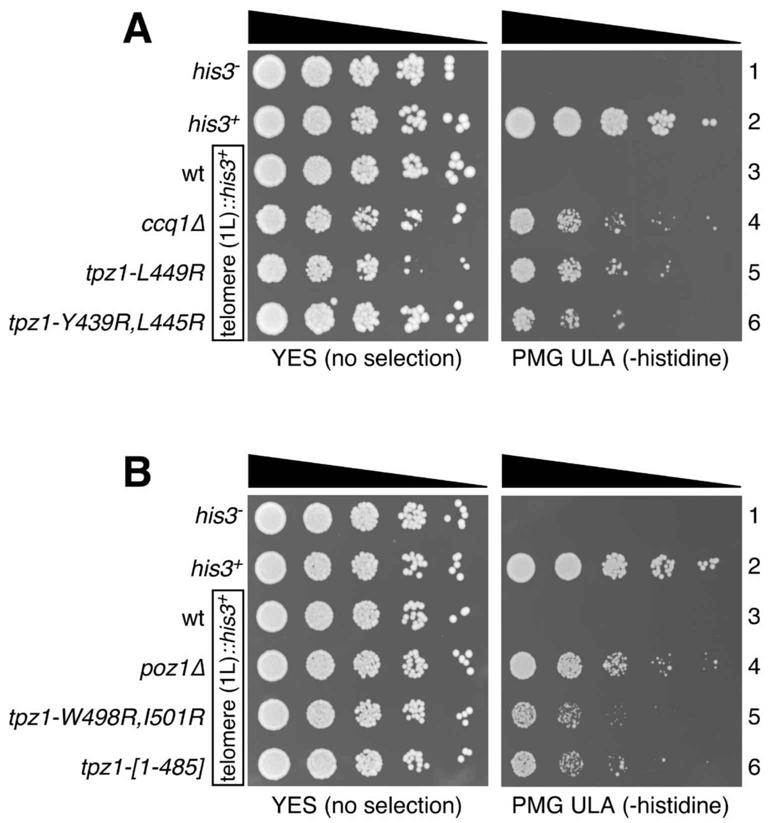

Figure S7. Heterochromatin formation at telomeres is affected in Tpz1-Ccq1 and Tpz1-Poz1 interaction mutants.

Wild-type, *ccq1*Δ, *poz1*Δ and *tpz1*-mutant cells carrying the *his3*$^+$ marker gene directly adjacent to the chromosome 1L telomere repeats were serially diluted and spotted on YES (no selection) or PMG ULA (–histidine) plates. (**A**) Similar to *ccq1*Δ, all Tpz1-Ccq1 interaction disruption mutations caused loss of transcriptional silencing at telomeres. (**B**) Similar to *poz1*Δ, all Tpz1-Poz1 interaction disruption mutations caused loss of transcriptional silencing at telomeres.

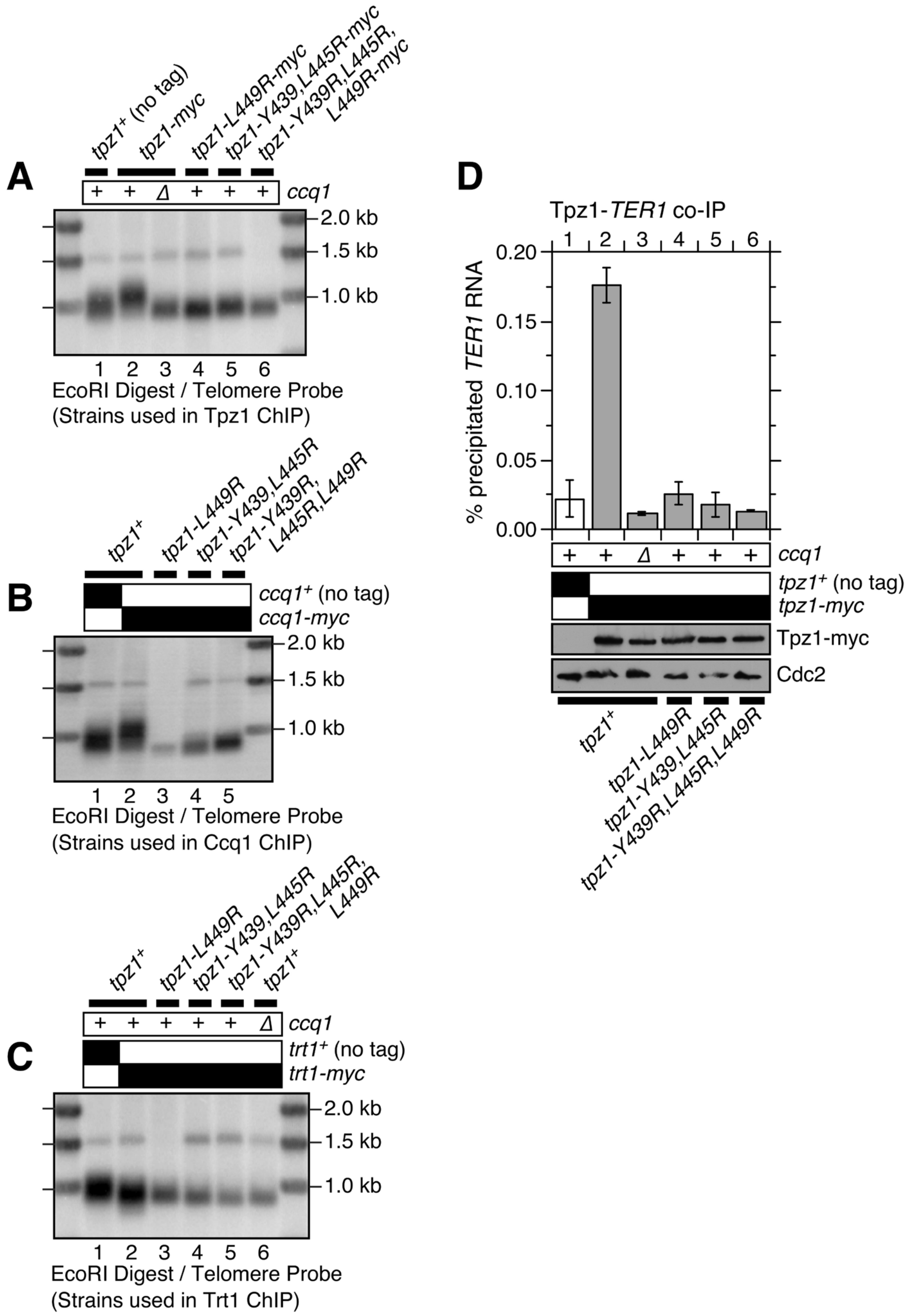

Figure S8. Characterization of Tpz1-Ccq1 interaction disruption mutant cells.

(**A**-**C**) Southern blot analysis of early generation mutant strains (20-40 cell divisions) utilized in ChIP analysis (Figure 5). (**D**) Binding of Tpz1 to telomerase RNA (*TER1*) was lost in Tpz1-Ccq1 interaction disruption mutants. Error bars indicate standard error of the mean from three to four independent experiments. Statistical analysis of *TER1* co-IP data by 2-tailed Student's t-test is shown in Table S5. Expression of Tpz1 was examined by anti-myc western blot, and Cdc2 served as loading control.

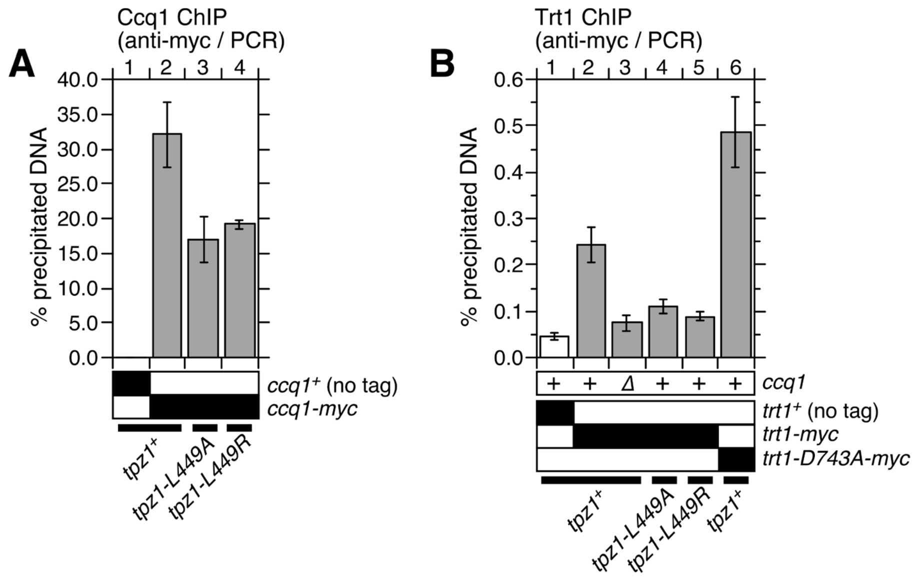

Figure S9. Comparison of *tpz1-L449A* and *tpz1-L449R* alleles for their effects on Ccq1 and Trt1^{TERT} localization to telomeres.

(**A**) Based on ChIP assay, Ccq1 showed comparable reduction in telomere association for both *L449A* and *L449R* alleles. (**B**) Comparable loss of Trt1 binding to telomeres was observed by ChIP assay for *ccq1Δ*, *tpz1-L449A* and *tpz1-L449R* cells. In contrast to these mutant cells that show telomere shortening due to loss of telomerase recruitment, catalytically dead Trt1 (*trt1-D743A*) showed increased binding at shorter telomeres [36]. Error bars represent standard error of the mean from three to eight independent experiments. Statistical analysis of ChIP data by 2-tailed Student's t-test is shown in Table S5.

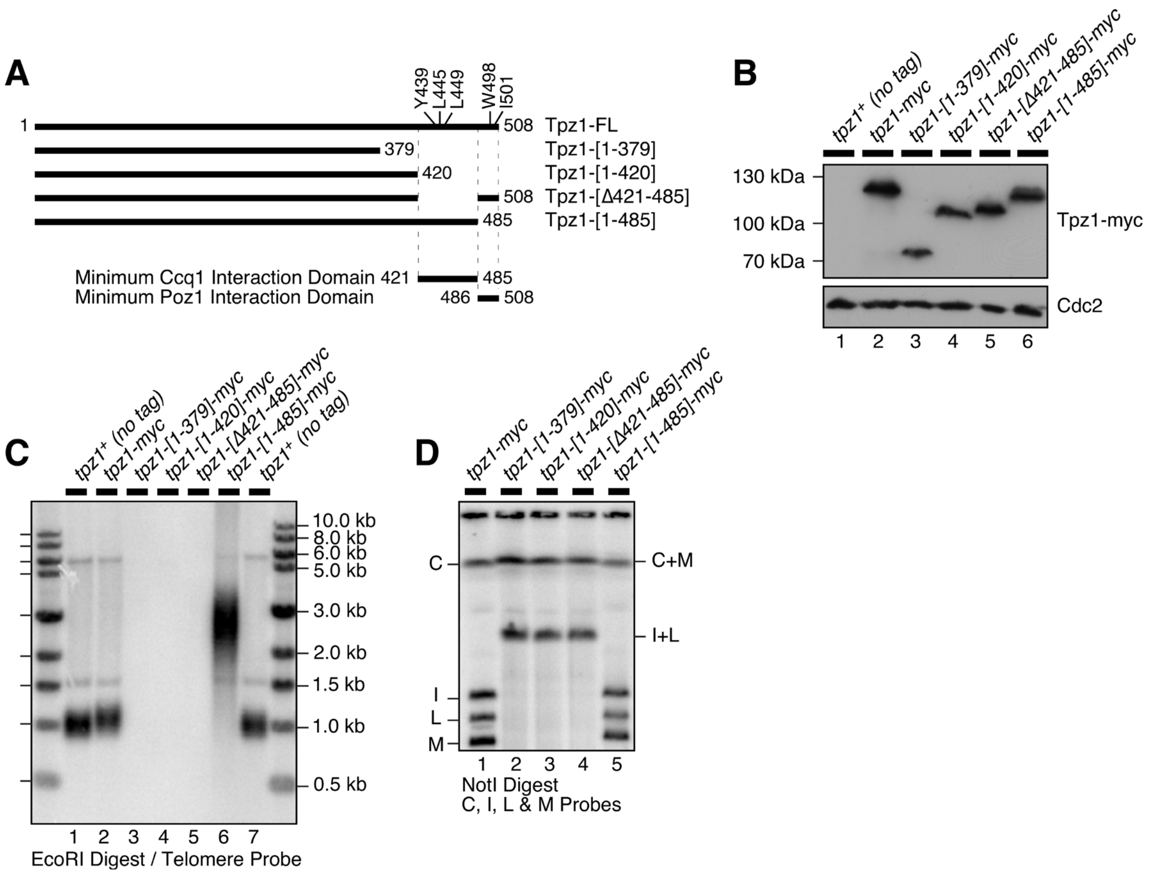

Figure S10. Effects of Tpz1 truncations on telomere maintenance.

(**A**) A schematic overview of Tpz1 truncation mutants. Minimum Ccq1 and Poz1 interaction domains are indicated. (**B**) Anti-myc western blot analysis to detect expression levels of myc-tagged wild-type and *tpz1*-truncation mutants in whole cell extract. Cdc2 served as loading control. (**C**) Southern blot analysis of wild-type and indicated truncation mutants hybridized to a telomeric repeat probe. For tpz1 truncation mutants, haploid cells derived from heterozygous diploid cells were restreaked 5 times on YES plates (estimated to be 100-125 cell divisions) prior to preparation of genomic DNA. (**D**) Pulsed-field gel analysis of wild-type and indicated *tpz1*-truncation mutants. Chromosomal DNA was prepared from cells after restreaked twice on YES plates (estimated to be 40-50 cell divisions), and telomeric NotI fragments were visualized by hybridization to C, I, L and M-specific probes. (See Figure 4B for a NotI-restriction site map of fission yeast chromosomes.)

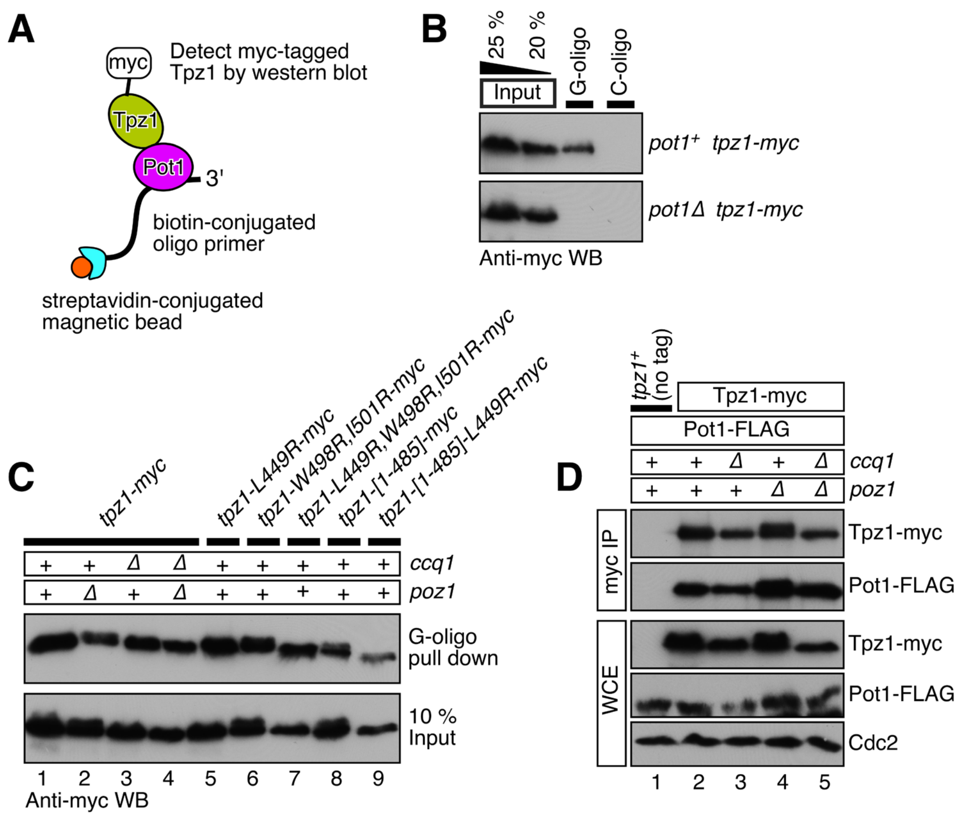

Figure S11. Binding of the Tpz1-Pot1 complex to telomere oligo primer does not depend on Tpz1-Ccq1 or Tpz1-Poz1 interaction.

(**A**) A schematic overview for the telomere oligonucleotide primer pull-down assay. Biotin-conjugated primers were bound to streptavidin-conjugated magnetic beads, and incubated with whole cell extracts from cells to monitor Pot1-dependent binding to Tpz1. (**B**) Tpz1 specifically associated with the telomeric G-oligo but not the complementary telomeric C-oligo. The interaction of Tpz1 with G-oligo was lost in *pot1Δ* cells. (See Materials and Methods.) (**C**) Tpz1-Pot1 interaction was not affected by Tpz1-Ccq1 or Tpz1-Poz1 interaction disruption mutants. (**D**) Tpz1-Pot1 interaction remained intact even in *ccq1Δ poz1Δ* cells. Interaction between Tpz1-myc and Pot1-FLAG was monitored by co-IP of Pot1-FLAG after anti-myc pull down of Tpz1-myc. For whole cell extract (WCE) western blot, Cdc2 served as a loading control.

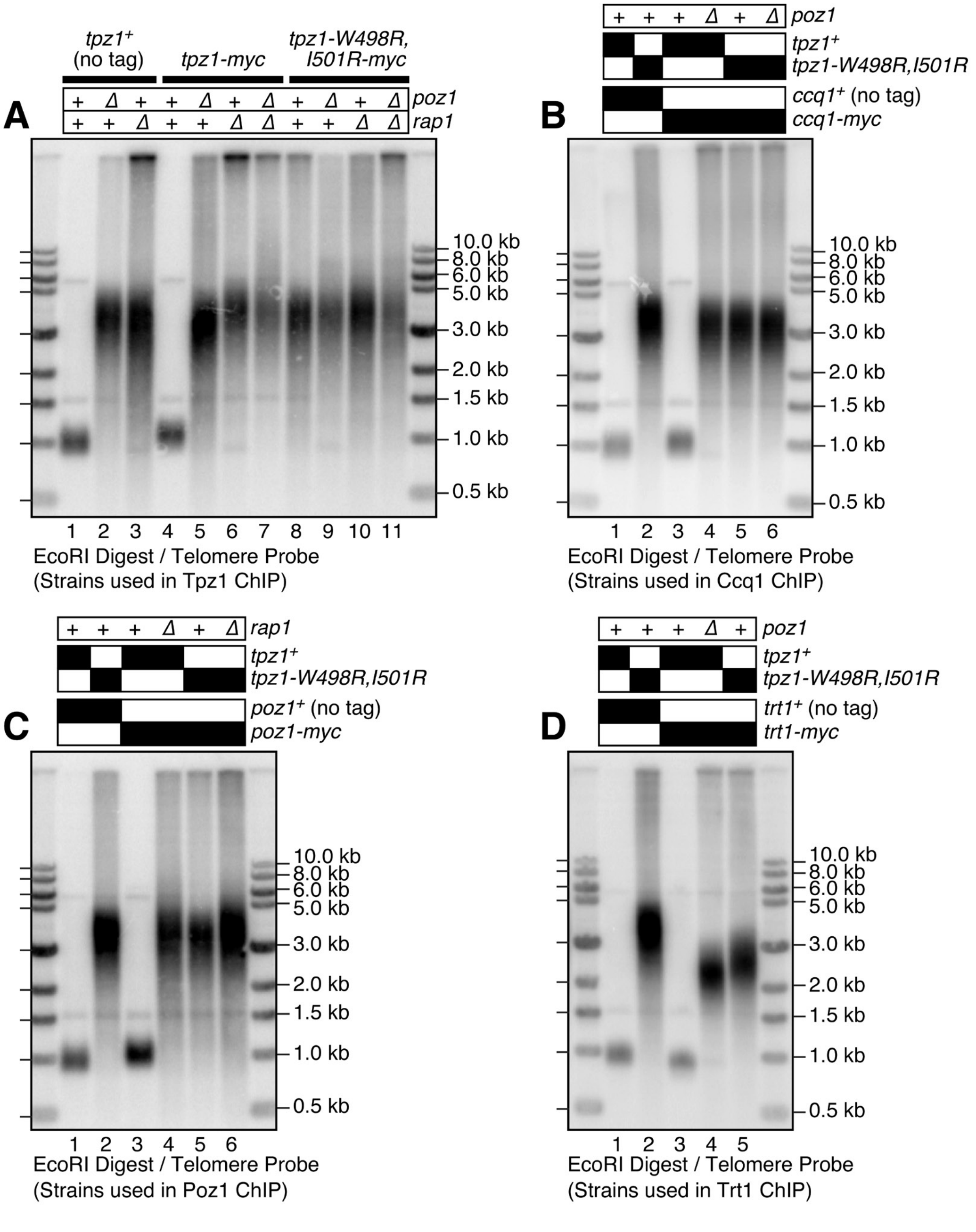

Figure S12. Characterization of Tpz1-Poz1 interaction disruption mutant cells.

Telomere length analysis by Southern blot was performed for strains used in (**A**) Tpz1, (**B**) Ccq1, (**C**) Poz1, and (**D**) Trt1^{TERT} ChIP assays (Figures 7A-D and S13).

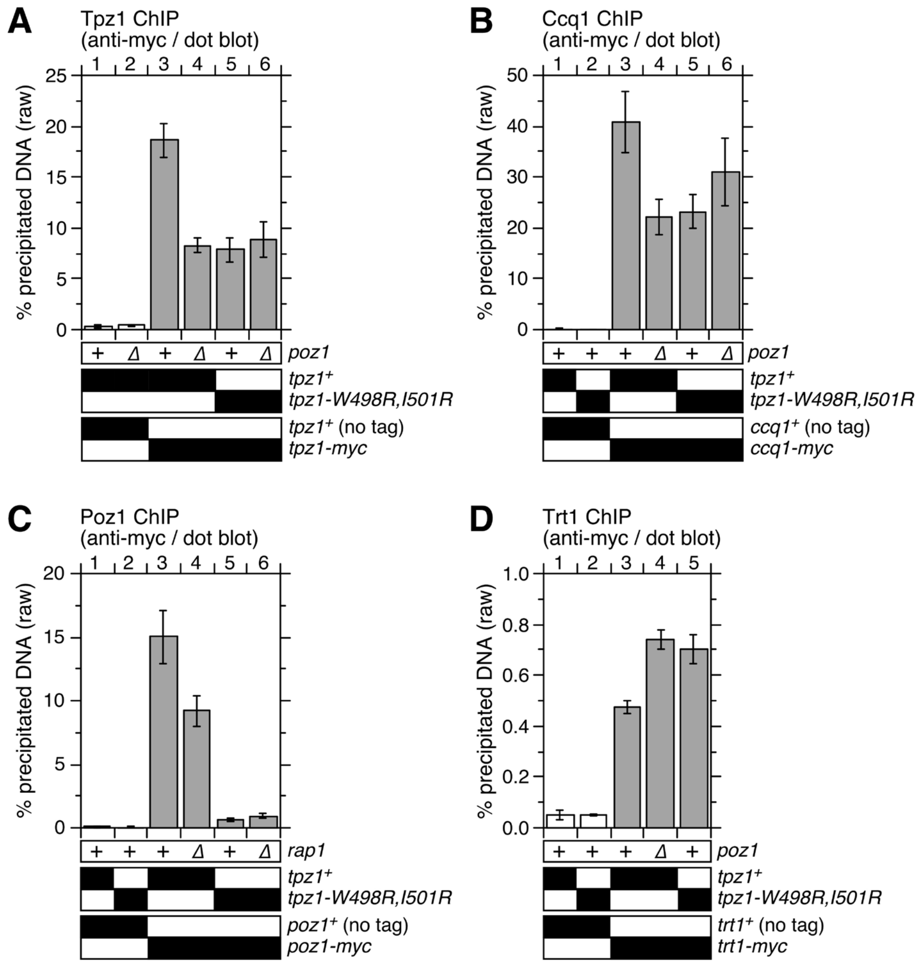

Figure S13. Raw data for Tpz1, Ccq1, Poz1 and Trt1^{TERT} ChIP assays in Tpz1-Poz1 interaction mutant cells.

Effects of disrupting Tpz1-Poz1 interaction on telomere association for (**A**) Tpz1, (**B**) Ccq1, (**C**) Poz1 and (**D**) Trt1^{TERT} were monitored by dot-blot ChIP assays and raw % precipitated DNA values were plotted. These data were then corrected for telomere length [36] to generate plots shown in Figure 7. Error bars represent standard error of the mean from three to eight independent experiments. Statistical analysis of ChIP data by 2-tailed Student's t-test is shown in Table S5.

Table S1 Telomere length correction factors (telomere/rDNA)

Tagged protein	Genetic background	Correction factor[a]
Tpz1-myc	wt	1.00 ± 0.08
	poz1Δ	4.72 ± 0.29
	tpz1-W498R,I501R	4.71 ± 0.28
	poz1Δ tpz1-W498R,I501R	4.86 ± 0.14
Ccq1-myc	wt	1.00 ± 0.03
	poz1Δ	2.63 ± 0.09
	tpz1-W498R,I501R	2.69 ± 0.08
	poz1Δ tpz1-W498R,I501R	2.63 ± 0.08
Poz1-myc	wt	1.00 ± 0.05
	rap1Δ	3.69 ± 0.14
	tpz1-W498R,I501R	3.78 ± 0.16
	rapΔ tpz1-W498R,I501R	4.01 ± 0.15
Trt1-myc	wt	1.00 ± 0.02
	poz1Δ	3.72 ± 0.14
	tpz1-W498R,I501R	4.90 ± 0.14

[a]Mean ± standard error of the mean. Values are normalized to wild-type cells with indicated tagged proteins.

Table S2 Fission yeast strains used in this study.

Figure		Strain		Full Genotype[a]
3B	1	TN7204	poz1-FLAG	h^{-} his3-D1 poz1^{+}::5FLAG-TEV-Avitag-kanMX6
	2	TN7553	poz1-FLAG tpz1-myc	h^{-} his3-D1 poz1^{+}::5FLAG-TEV-Avitag-kanMX6 tpz1^{+}::13myc-kanMX6
	3	JH11179	poz1-FLAG tpz1-L449R-myc	h^{+} ade6-M216 his3-D1 poz1^{+}::5FLAG-TEV-Avitag-kanMX6 tpz1-L449R::13myc-kanMX6
	4	JH11241	poz1-FLAG tpz1-Y439R,L445R-myc	h^{-} his3-D1 poz1^{+}::5FLAG-TEV-Avitag-kanMX6 tpz1-Y439R,L445R::13myc-kanMX6
	5	JH11271	poz1-FLAG tpz1-Y439R,L445R,L449R-myc	h^{+} his3-D1 poz1^{+}::5FLAG-TEV-Avitag-kanMX6 tpz1-Y439R,L445R,L449R-13myc-kanMX6
3C	1	TN6770	ccq1-FLAG	h^{+} his3-D1 ccq1^{+}::5FLAG-kanMX6
	2	TN7505	ccq1-FLAG tpz1-myc	h^{+} his3-D1 ccq1^{+}::5FLAG-kanMX6 tpz1^{+}::13myc-kanMX6
	3	JH11174	ccq1-FLAG tpz1-L449R-myc	h^{-} ccq1^{+}::5FLAG-kanMX6 tpz1-L449R::13myc-kanMX6
	4	JH11238	ccq1-FLAG tpz1-Y439R,L445R-myc	h^{-} his3-D1 ccq1^{+}::5FLAG-kanMX6 tpz1-Y439R,L445R::13myc-kanMX6
	5	JH11248	ccq1-FLAG tpz1-Y439R,L445R,L449R-myc	h^{+} his3-D1 ccq1^{+}::5FLAG-kanMX6 tpz1-Y439R,L445R,L449R-13myc-kanMX6
3D	1	YTC9716	pot1-FLAG	h^{+} his3-D1 pot1^{+}::3FLAG-kanMX
	2	JH11046	pot1-FLAG tpz1-myc	h^{-} his3-D1 pot1^{+}::3FLAG-kanMX tpz1^{+}::13myc-kanMX6
	3	JH11176	pot1-FLAG tpz1-L449R-myc	h^{+} pot1^{+}::3FLAG-kanMX tpz1-L449R::13myc-kanMX6
	4	JH11244	pot1-FLAG tpz1-Y439R,L445R-myc	h^{+} ade6-M210 his3-D1 pot1^{+}::3FLAG-kanMX tpz1-Y439R,L445R::13myc-kanMX6
	5	JH11273	pot1-FLAG tpz1-Y439R,L445R,L449R-myc	h^{-} pot1^{+}::3FLAG-kanMX tpz1-Y439R,L445R,L449R-13myc-kanMX6
3E	1	TN7553	poz1-FLAG tpz1-myc	h^{-} his3-D1 poz1^{+}::5FLAG-TEV-Avitag-kanMX6 tpz1^{+}::13myc-kanMX6
	2	JH11047	poz1-FLAG tpz1-[1-485]-myc	h^{+} ade6-M210 his3-D1 poz1^{+}::5FLAG-TEV-Avitag-kanMX6 tpz1-[1-485]::13myc-kanMX6
	3	JH11200	poz1-FLAG tpz1-W498R,I501R-myc	h^{+} ade6-M216 his3-D1 poz1^{+}::5FLAG-TEV-Avitag-kanMX6 tpz1-W498R,I501R::13myc-kanMX6
	4	TN7196	tpz1-myc	h^{-} his3-D1 tpz1^{+}::13myc-kanMX6
3F	1	TN6770	ccq1-FLAG	h^{+} his3-D1 ccq1^{+}::5FLAG-kanMX6
	2	TN7505	ccq1-FLAG tpz1-myc	h^{+} his3-D1 ccq1^{+}::5FLAG-kanMX6 tpz1^{+}::13myc-kanMX6
	3	JH10926	ccq1-FLAG tpz1-[1-485]-myc	h^{-} his3-D1 ccq1^{+}::5FLAG-kanMX6 tpz1-[1-485]::13myc-kanMX6
	4	JH11203	ccq1-FLAG tpz1-W498R,I501R-myc	h^{-} his3-D1 ccq1^{+}::5FLAG-kanMX6 tpz1-W498R,I501R::13myc-kanMX6
3G	1	YTC9716	pot1-FLAG	h^{+} his3-D1 pot1^{+}::3FLAG-kanMX
	2	JH11046	pot1-FLAG tpz1-myc	h^{-} his3-D1 pot1^{+}::3FLAG-kanMX tpz1^{+}::13myc-kanMX6
	3	JH10929	pot1-FLAG tpz1-[1-485]-myc	h^{+} his3-D1 pot1^{+}::3FLAG-kanMX tpz1-[1-485]::13myc-kanMX6
	4	JH11196	pot1-FLAG tpz1-W498R,I501R-myc	h^{+} pot1^{+}::3FLAG-kanMX tpz1-W498R,I501R::13myc-kanMX6
4A	1	CF484	tpz1^{+}/tpz1^{+}	h^{+}/h^{-} ade6-M210/ade6-M216 his3-D1/his3-D1
	2,16	TN2411	tpz1^{+}	h^{-} his3-D1
	3	JH11365	tpz1^{+}/tpz1-myc	h^{+}/h^{-} ade6-M210/ade6-M216 his3-D1/his3-D1 tpz1^{+}/tpz1^{+}::13myc-kanMX6
	4,15	TN7196	tpz1-myc	h^{-} his3-D1 tpz1^{+}::13myc-kanMX6
	5	JH11306	tpz1^{+}/tpz1-L449R-myc	h^{+}/h^{-} ade6-M210/ade6-M216 his3^{+}/his3-D1 tpz1^{+}/tpz1-L449R::13myc-kanMX6
	6	JH11835	tpz1-L449R-myc (2x)	h^{+} ade6-M210 his3-D1 tpz1-L449R::13myc-kanMX6 (2x restreaks)
	7	JH12260	tpz1-L449R-myc (5x)	h^{+} ade6-M210 his3-D1 tpz1-L449R::13myc-kanMX6 (5x restreaks)
	8	JH11495	tpz1^{+}/tpz1-Y439R,L445R-myc	h^{+}/h^{-} ade6-M210/ade6-M216 his3^{+}/his3-D1 tpz1^{+}/tpz1-L439R,L445R::13myc-kanMX6
	9	JH11496	tpz1-Y439R,L445R-myc (2x)	h^{-} ade6-M210 his3-D1 tpz1-L439R,L445R::13myc-kanMX6 (2x restreaks)
	10	JH12261	tpz1-Y439R,L445R-myc (5x)	h^{-} ade6-M210 his3-D1 tpz1-L439R,L445R::13myc-kanMX6 (5x restreaks)
	11	JH11500	tpz1^{+}/tpz1-Y439R,L445R,L449R-myc	h^{+}/h^{-} ade6-M210/ade6-M216 his3^{+}/his3-D1 tpz1^{+}/tpz1-L439R,L445R,L449R::13myc-kanMX6
	12	JH11501	tpz1-Y439R,L445R,L449R-myc (2x)	h^{-} ade6-M216 his3-D1 tpz1-L439R,L445R,L449R::13myc-kanMX6 (2x restreaks)
	13	JH12262	tpz1-Y439R,L445R,L449R-myc (5x)	h^{-} ade6-M216 his3-D1 tpz1-L439R,L445R,L449R::13myc-kanMX6 (5x restreaks)
	14	TN9009	tpz1-myc ccq1Δ	h^{+} his3-D1 tpz1-13myc:KanMX6 ccq1Δ::hphMX

Figure		Strain		Full Genotype[a]
4B	1	TN11289	tpz1$^+$	h$^-$ his3-D1 tpz1$^+$::hphMX
	2	CF448	trt1Δ	h$^-$ ade6-M210 his3-D1 trt1Δ::his3$^+$
	3	TN9258	ccq1Δ	h$^-$ his3-D1 ccq1Δ::hphMX (linear)
	4	TN9259	ccq1Δ	h$^-$ his3-D1 ccq1Δ::hphMX (circular)
	5	JH12993	tpz1-L449R	h$^?$ ade6-M210 or M216 his3-D1 tpz1-L449R::hphMX
	6	JH12995	tpz1-L449R	h$^?$ ade6-M210 or M216 his3-D1 tpz1-L449R::hphMX
	7	JH12986	tpz1-Y439R,L445R	h$^?$ ade6-M210 or M216 his3-D1 tpz1-Y439R,L445R::hphMX
	8	JH12988	tpz1-Y439R,L445R	h$^?$ ade6-M210 or M216 his3-D1 tpz1-Y439R,L445R::hphMX
	9	JH12990	tpz1-Y439R,L445R,L449R	h$^?$ ade6-M210 or M216 his3-D1 tpz1-Y439R,L445R,L449R::hphMX
	10	JH12992	tpz1-Y439R,L445R,L449R	h$^?$ ade6-M210 or M216 his3-D1 tpz1-Y439R,L445R,L449R::hphMX
4C	1,7,13	TN2411	tpz1$^+$ ccq1$^+$ poz1$^+$	h$^-$ his3-D1
	2	TN7196	tpz1-myc	h$^-$ his3-D1 tpz1$^+$::13myc-kanMX6
	3	JH12998	tpz1-L449R-myc	h$^-$ ade6-M216 his3-D1 tpz1-L449R::13myc-kanMX6
	4	JH13002	ccq1Δ	h$^-$ his3-D1 ccq1Δ::hphMX
	5	TN9009	ccq1Δ tpz1-myc	h$^+$ his3-D1 ccq1Δ::hphMX tpz1$^+$::13myc-kanMX6
	6	JH11684	ccq1Δ tpz1-L449R-myc	h$^+$ his3-D1 ccq1Δ::hphMX tpz1-L449R-13myc:KanMX6
	8	JH12999	poz1Δ	h$^-$ ade6-M216 his3-D1 poz1Δ::natMX6
	9	TN9254	poz1Δ ccq1Δ	h$^-$ his3-D1 poz1Δ::natMX6 ccq1Δ::hphMX
	10	YTC9366	poz1Δ tpz1-myc	h$^-$ his3-D1 poz1Δ::natMX6 tpz1$^+$::13myc-kanMX6
	11	JH11701	poz1Δ ccq1Δ tpz1-myc	h$^+$ his3-D1 poz1Δ::natMX6 ccq1Δ::hphMX tpz1$^+$::13myc-kanMX6
	12	JH11686	poz1Δ tpz1-L449R-myc	h$^-$ ade6-M216 his3-D1 poz1Δ::natMX6 tpz1-L449R::13myc-kanMX6
4D	1	TN7196	tpz1-myc	h$^-$ his3-D1 tpz1$^+$::13myc-kanMX6
	2	YTC9366	poz1Δ tpz1-myc	h$^-$ his3-D1 poz1Δ::natMX6 tpz1$^+$::13myc-kanMX6
	3	TN9009	ccq1Δ tpz1-myc	h$^+$ his3-D1 ccq1Δ::hphMX tpz1$^+$::13myc-kanMX6
	4	JH11701	poz1Δ ccq1Δ tpz1-myc	h$^+$ his3-D1 poz1Δ::natMX6 ccq1Δ::hphMX tpz1$^+$::13myc-kanMX6
	5	JH11835	tpz1-L449R-myc	h$^+$ ade6-M210 his3-D1 tpz1-L449R::13myc-kanMX6
	6	JH11686	poz1Δ tpz1-L449R-myc	h$^-$ ade6-M216 his3-D1 poz1Δ::natMX6 tpz1-L449R::13myc-kanMX6
	7	JK11683	ccq1Δ tpz1-L449R-myc	h$^+$ his3-D1 ccq1Δ::hphMX tpz1-L449R::13myc-kanMX6
5A	1	TN2411	tpz1$^+$ (no tag)	h$^-$ his3-D1
	2	TN7196	tpz1-myc	h$^-$ his3-D1 tpz1$^+$::13myc-kanMX6
	3	TN9009	tpz1-myc ccq1Δ	h$^+$ his3-D1 tpz1$^+$::13myc-kanMX6 ccq1Δ::hphMX
	4	JH11835	tpz1-L449R-myc	h$^+$ ade6-M210 his3-D1 tpz1-L449R::13myc-kanMX6
	5	JH11496	tpz1-Y439R,L445R-myc	h$^-$ ade6-M210 his3-D1 tpz1-L439R,L445R::13myc-kanMX6
	6	JH11232	tpz1-Y439R,L445R,L449R-myc	h$^+$ ade6-M210 his3-D1 tpz1-Y439R,L445R,L449R::13myc-kanMX6
5B	1	JH11289	ccq1$^+$ (no tag)	h$^-$ his3-D1 tpz1$^+$::hphMX
	2	JH11569	ccq1-myc	h$^+$ his3-D1 ccq1$^+$::13myc-kanMX6 tpz1$^+$::hphMX
	3	JH11622	ccq1-myc tpz1-L449R	h$^+$ his3-D1 ccq1$^+$::13myc-kanMX6 tpz1-L449R::hphMX
	4	JH11452	ccq1-myc tpz1-Y439R,L445R	h$^+$ his3-D1 ccq1$^+$::13myc-kanMX6 tpz1-Y439R,L445R::hphMX
	5	JH11609	ccq1-myc tpz1-Y439R,L445R,L449R	h$^+$ his3-D1 ccq1$^+$::13myc-kanMX6 tpz1-Y439R,L445R,L449R::hphMX
5C	1	JH11289	trt1$^+$ (no tag)	h$^-$ his3-D1 tpz1$^+$::hphMX
	2	JH11630	trt1-myc	h$^-$ ade6-M216 his3-D1 trt1$^+$::G$_8$-13myc-kanMX6 tpz1$^+$::hphMX
	3	JH11626	trt1-myc tpz1-L449R	h$^-$ ade6-M216 his3-D1 trt1$^+$::G$_8$-13myc-kanMX6 tpz1-L449R::hphMX
	4	JH11449	trt1-myc tpz1-Y439R,L445R	h$^+$ ade6-M210 his3-D1 trt1$^+$::G$_8$-13myc-kanMX6 tpz1-Y439R,L445R::hphMX
	5	JH11611	trt1-myc tpz1-Y439R,L445R,L449R	h$^-$ ade6-M210 his3-D1 trt1$^+$::G$_8$-13myc-kanMX6 tpz1-Y439R,L445R,L449R::hphMX
	6	JH11644	trt1-myc ccq1Δ	h$^+$ his3-D1 trt1$^+$::G$_8$-13myc-kanMX6 tpz1$^+$::hphMX ccq1Δ::hphMX
5D	1	TN6770	ccq1-FLAG	h$^+$ his3-D1 ccq1$^+$::5FLAG-kanMX6
	2	TN10988	trt1Δ ccq1-FLAG	h$^-$ ade6-M210 his3-D1 trt1Δ::his3$^+$ ccq1$^+$::5FLAG-kanMX6
	3	TN10991	trt1Δ ccq1-T93A-FLAG	h$^-$ ade6-M210 his3-D1 trt1Δ::his3$^+$ ccq1-T93A::5FLAG-kanMX6
	4	JH11174	ccq1-FLAG tpz1-L449R-myc	h$^-$ ccq1$^+$::5FLAG-kanMX6 tpz1-L449R::13myc-kanMX6
	5	JH11238	ccq1-FLAG tpz1-Y439R,L445R-myc	h$^-$ his3-D1 ccq1$^+$::5FLAG-kanMX6 tpz1-Y439R,L445R::13myc-kanMX6
	6	JH11248	ccq1-FLAG tpz1-Y439R,L445R,L449R-myc	h$^+$ his3-D1 ccq1$^+$::5FLAG-kanMX6 tpz1-Y439R,L445R,L449R-13myc-kanMX6

Figure		Strain		Full Genotype[a]
5E	1	TN6770	ccq1-FLAG	h^+ his3-D1 ccq1$^+$::5FLAG-kanMX6
	2	TN10535	rap1Δ ccq1-FLAG	h^- his3-D1 rap1Δ::ura4$^+$ ccq1$^+$::5FLAG-kanMX6
	3	BAM10829	rap1Δ ccq1-T93A-FLAG	h^- his3-D1 rap1Δ::ura4$^+$ ccq1-T93A::5FLAG-kanMX6
	4	JH11544	rap1Δ tpz1-L449R-myc ccq1-FLAG	h^- his3-D1 rap1Δ::ura4$^+$ tpz1-L449R-13myc-kanMX6 ccq1$^+$::5FLAG-kanMX6
	5	JH11546	rap1Δ tpz1-Y439R,L445R-myc ccq1-FLAG	h^- his3-D1 rap1Δ::ura4$^+$ tpz1-Y439R,L445R-13myc-kanMX6 ccq1$^+$::5FLAG-kanMX6
	6	JH11548	rap1Δ tpz1-Y439R,L445R,L449R-myc ccq1-FLAG	h^+ his3-D1 rap1Δ::ura4$^+$ tpz1-Y439R,L445R,L449R-13myc-kanMX6 ccq1$^+$::5FLAG-kanMX6
6A	1	JH11365	tpz1$^+$/tpz1-myc	h^+/h^- ade6-M210/ade6-M216 his3-D1/his3-D1 tpz1$^+$/tpz1$^+$::13myc-kanMX6
	2	JH11715	tpz1$^+$/tpz1-[1-485]-myc	h^+/h^- ade6-M210/ade6-M216 his3-D1/his3-D1 tpz1$^+$/tpz1-[1-485]::13myc-kanMX6
	3	JH11189	tpz1$^+$/tpz1-W498R,I501R-myc	h^+/h^- ade6-M210/ade6-M216 his3-D1/his3-D1 tpz1$^+$/tpz1-W498R,I501R::13myc-kanMX6
	4	TN2411	tpz1$^+$	h^- his3-D1
	5	TN7196	tpz1-myc	h^- his3-D1 tpz1$^+$::13myc-kanMX6
	6	JH13000	poz1Δ	h^- ade6-M216 his3-D1 poz1Δ::natMX6
	7	YTC9366	tpz1-myc poz1Δ	h^- his3-D1 tpz1$^+$::13myc-kanMX6 poz1Δ::natMX6
	8	JH10923	tpz1-[1-485]-myc	h^- ade6-M210 his3-D1 tpz1-[1-485]::13myc-kanMX6
	9	JH13004	tpz1-W498R,I501R-myc	h^+ ade6-M216 his3-D1 tpz1-W498R,I501R::13myc-kanMX6
	10	JH11693	tpz1-W498R,I501R-myc poz1Δ	h^- ade6-M216 his3-D1 tpz1-W498R,I501R::13myc-kanMX6 poz1Δ::natMX6
	11	JH11689	tpz1-W498R,I501R-myc ccq1Δ	h^- his3-D1 tpz1-W498R,I501R::13myc-kanMX6 ccq1Δ::hphMX
	12	JH11701	tpz1-myc poz1Δ ccq1Δ	h^+ his3-D1 tpz1$^+$::13myc-kanMX6 poz1Δ::natMX6 ccq1Δ::hphMX
6B	1	TN7196	tpz1-myc	h^- his3-D1 tpz1$^+$::13myc-kanMX6
	2	JH11190	tpz1-W498R,I501R-myc	h^+ leu1-32 ura4-D18 ade6-M216 his3-D1 tpz1-W498R,I501R::13myc-kanMX6
	3	JH11692	tpz1-W498R,I501R-myc poz1Δ	h^- leu1-32 ura4-D18 ade6-M216 his3-D1 tpz1-W498R,I501R::13myc-kanMX6 poz1Δ::natMX6
	4	JH11689	tpz1-W498R,I501R-myc ccq1Δ	h^- his3-D1 tpz1-W498R,I501R::13myc-kanMX6 ccq1Δ::hphMX
	5	JH11701	tpz1-myc poz1Δ ccq1Δ	h^+ his3-D1 tpz1$^+$::13myc-kanMX6 poz1Δ::natMX6 ccq1Δ::hphMX
6C	1,8	TN2411	tpz1$^+$	h^- his3-D1
	2	TN7196	tpz1-myc	h^- his3-D1 tpz1$^+$::13myc-kanMX6
	3	JH11835	tpz1-L449R-myc	h^+ ade6-M210 his3-D1 tpz1-L449R::13myc-kanMX6
	4	JH10922	tpz1-[1-485]-myc	h^- ade6-M210 his3-D1 tpz1-[1-485]::13myc-kanMX6
	5	JH11790	tpz1-[1-485]-L449R-myc	h^- ade6-M216 his3-D1 tpz1-[1-485]-L449R::13myc-kanMX6
	6	JH11190	tpz1-W498R,I501R-myc	h^+ ade6-M216 his3-D1 tpz1-W498R,I501R::13myc-kanMX6
	7	JH11781	tpz1-L449R,W498R,I501R-myc	h^+ ade6-M216 his3-D1 tpz1-L449R,W498R,I501R::13myc-kanMX6
6D	1	JH11835	tpz1-L449R-myc	h^+ ade6-M210 his3-D1 tpz1-L449R::13myc-kanMX6
	2	JH10922	tpz1-[1-485]-myc	h^- ade6-M210 his3-D1 tpz1-[1-485]::13myc-kanMX6
	3	JH11790	tpz1-[1-485]-L449R-myc	h^- ade6-M216 his3-D1 tpz1-[1-485]-L449R::13myc-kanMX6
	4	JH11190	tpz1-W498R,I501R-myc	h^+ ade6-M216 his3-D1 tpz1-W498R,I501R::13myc-kanMX6
	5	JH11781	tpz1-L449R,W498R,I501R-myc	h^+ ade6-M216 his3-D1 tpz1-L449R,W498R,I501R::13myc-kanMX6
7A	1	TN7196	tpz1-myc	h^- his3-D1 tpz1$^+$::13myc-kanMX6
	2	YTC9366	tpz1-myc poz1Δ	h^- his3-D1 tpz1$^+$::13myc-kanMX6 poz1Δ::natMX6
	3	JH11191	tpz1-W498R,I501R-myc	h^+ ade6-M216 his3-D1 tpz1-W498R,501R::13myc-kanMX6
	4	JH11692	tpz1-W498R,I501R-myc poz1Δ	h^- ade6-M216 his3-D1 tpz1-W498R,I501R::13myc-kanMX6 poz1Δ::natMX6
7B	1	TN7217	ccq1-myc	h^- his3-D1 ccq1$^+$::13myc-kanMX6
	2	YTC8712	ccq1-myc poz1Δ	h^+ his3-D1 ccq1$^+$::13myc-kanMX6 poz1Δ::natMX6
	3	JH12895	ccq1-myc tpz1-W498R,I501R	h^+ his3-D1 ccq1$^+$::13myc-kanMX6 tpz1-W498R,I501R::hphMX
	4	JH12897	ccq1-myc tpz1-W498R,I501R poz1Δ	h^- his3-D1 ccq1$^+$::13myc-kanMX6 tpz1-W498R,I501R::hphMX poz1Δ::natMX6

Figure		Strain		Full Genotype[a]
7C	1	JH12265	*poz1-myc*	*h$^+$ his3-D1 poz1$^+$::13myc-kanMX6 tpz1$^+$::hphMX*
	2	JH12267	*poz1-myc rap1Δ*	*h$^+$ his3-D1 poz1$^+$::13myc-kanMX6 tpz1$^+$::hphMX rap1Δ::ura4$^+$*
	3	JH12659	*poz1-myc tpz1-W498R,I501R*	*h$^+$ ade6-M210 his3-D1 poz1$^+$::13myc-kanMX6 tpz1-W498R,I501R::hphMX*
	4	JH12056	*poz1-myc tpz1-W498R,I501R rap1Δ*	*h$^+$ ade6-M210 his3-D1 poz1$^+$::13myc-kanMX6 tpz1-W498R,I501R::hphMX rap1Δ::ura4$^+$*
7D	1	JH11630	*trt1-myc*	*h$^-$ ade6-M216 his3-D1 trt1$^+$::G$_8$-13myc-kanMX6 tpz1$^+$::hphMX*
	2	JH12770	*trt1-myc poz1Δ*	*h$^-$ ade6-M216 his3-D1 trt1$^+$::G$_8$-13myc-kanMX6 tpz1$^+$::hphMX poz1Δ::natMX6*
	3	JH12766	*trt1-myc tpz1-W498R,I501R*	*h$^-$ his3-D1 trt1$^+$::G$_8$-13myc-kanMX6 tpz1-W498R,I501R::hphMX*
7E	1	TN6770	*ccq1-FLAG*	*h$^+$ his3-D1 ccq1$^+$::5FLAG-kanMX6*
	2	TN10531	*ccq1-FLAG poz1Δ*	*h$^-$ his3-D1 ccq1$^+$::5FLAG-kanMX6 poz1Δ::natMX6*
	3	TN12036	*ccq1-T93A-FLAG poz1Δ*	*h$^-$ his3-D1 ccq1-T93A::5FLAG-kanMX6 poz1Δ::natMX6*
	4	JH10926	*ccq1-FLAG tpz1-[1-485]-myc*	*h$^-$ his3-D1 ccq1$^+$::5FLAG-kanMX6 tpz1-[1-485]::13myc-kanMX6*
	5	JH11203	*ccq1-FLAG tpz1-W498R,I501R-myc*	*h$^-$ his3-D1 ccq1$^+$::5FLAG-kanMX6 tpz1-W498R,I501R::13myc-kanMX6*
	6	JH11772	*ccq1-FLAG tpz1-W498R,I501R-myc poz1Δ*	*h$^+$ ade6-M216 his3-D1 ccq1$^+$::5FLAG-kanMX6 tpz1-W498R,I501R::13myc-kanMX6 poz1Δ::natMX6*
S3A	1,5	TN6770	*ccq1-FLAG*	*h$^+$ his3-D1 ccq1$^+$::5FLAG-kanMX6*
	2,6	TN7505	*ccq1-FLAG tpz1-myc*	*h$^+$ his3-D1 ccq1$^+$::5FLAG-kanMX6 tpz1$^+$::13myc-kanMX6*
	3,7	JH11174	*ccq1-FLAG tpz1-L449R-myc*	*h$^-$ ccq1$^+$::5FLAG-kanMX6 tpz1-L449R::13myc-kanMX6*
	4,8	JH12780	*ccq1-FLAG tpz1-L449A-myc*	*h$^-$ his3-D1 ccq1$^+$::5FLAG-kanMX6 tpz1-L449A::13myc-kanMX6*
S3B	1	TN14536	*tpz1$^+$/tpz1-L449A*	*h$^+$/h$^-$ ade6-M210/ade6-M216 his3-D1/his3-D1 tpz1$^+$/tpz1-L449A::hphMX*
	2	TN14539	*tpz1-L449A* (2x)	*h$^-$ ade6-M216 his3-D1 tpz1-L449A::hphMX* (2x restreaks)
	3	TN14577	*tpz1-L449A* (5x)	*h$^-$ ade6-M216 his3-D1 tpz1-L449A::hphMX* (5x restreaks)
	4	TN14540	*tpz1-L449A* (2x)	*h$^-$ ade6-M216 his3-D1 tpz1-L449A::hphMX* (2x restreaks)
	5	TN14579	*tpz1-L449A* (5x)	*h$^-$ ade6-M216 his3-D1 tpz1-L449A::hphMX* (5x restreaks)
	6	TN14541	*tpz1-L449A* (2x)	*h$^-$ ade6-M216 his3-D1 tpz1-L449A::hphMX* (2x restreaks)
	7	TN14581	*tpz1-L449A* (5x)	*h$^-$ ade6-M216 his3-D1 tpz1-L449A::hphMX* (5x restreaks)
	8	TN14542	*tpz1-L449A* (2x)	*h$^-$ ade6-M216 his3-D1 tpz1-L449A::hphMX* (2x restreaks)
	9	TN14583	*tpz1-L449A* (5x)	*h$^-$ ade6-M216 his3-D1 tpz1-L449A::hphMX* (5x restreaks)
	10	TN11289	*tpz1$^+$*	*h$^-$ his3-D1 tpz1$^+$::hphMX*
S3C	1	TN11289	*tpz1$^+$*	*h$^-$ his3-D1 tpz1$^+$::hphMX*
	2	TN14578	*tpz1-L449A*	*h$^-$ ade6-M216 his3-D1 tpz1-L449A::hphMX*
	3	TN14580	*tpz1-L449A*	*h$^-$ ade6-M216 his3-D1 tpz1-L449A::hphMX*
	4	TN14582	*tpz1-L449A*	*h$^-$ ade6-M216 his3-D1 tpz1-L449A::hphMX*
	5	TN14584	*tpz1-L449A*	*h$^-$ ade6-M216 his3-D1 tpz1-L449A::hphMX*
S3D	1,12	TN11289	*tpz1$^+$*	*h$^-$ his3-D1 tpz1$^+$::hphMX*
	2	JH12999	*poz1Δ*	*h$^-$ ade6-M216 his3-D1 poz1Δ::natMX6*
	3	TN14539	*tpz1-L449A*	*h$^-$ ade6-M216 his3-D1 tpz1-L449A::hphMX*
	4	TN14540	*tpz1-L449A*	*h$^-$ ade6-M216 his3-D1 tpz1-L449A::hphMX*
	5	TN14574	*tpz1-L449A poz1Δ*	*h$^-$ ade6-M216 his3-D1 tpz1-L449A::hphMX poz1Δ::natMX6*
	6	TN14575	*tpz1-L449A poz1Δ*	*h$^+$ his3-D1 tpz1-L449A::hphMX poz1Δ::natMX6*
	7	TN14576	*tpz1-L449A poz1Δ*	*h$^-$ ade6-M216 his3-D1 tpz1-L449A::hphMX poz1Δ::natMX6*
	8	JH11577	*tpz1-L449R*	*h$^+$ ade6-M216 his3-D1 tpz1-L449R::hphMX*
	9	TN14444	*tpz1-L449R poz1Δ*	*h$^-$ his3-D1 tpz1-L449R::hphMX poz1Δ::natMX6*
	10	TN14445	*tpz1-L449R poz1Δ*	*h$^+$ ade6-M216 his3-D1 tpz1-L449R::hphMX poz1Δ::natMX6*
	11	TN14446	*tpz1-L449R poz1Δ*	*h$^+$ ade6-M216 his3-D1 tpz1-L449R::hphMX poz1Δ::natMX6*
S3E	1	TN11289	*tpz1$^+$*	*h$^-$ his3-D1 tpz1$^+$::hphMX*
	2	TN14539	*tpz1-L449A*	*h$^-$ ade6-M216 his3-D1 tpz1-L449A::hphMX*
	3	TN14540	*tpz1-L449A*	*h$^-$ ade6-M216 his3-D1 tpz1-L449A::hphMX*
	4	TN14574	*tpz1-L449A poz1Δ*	*h$^-$ ade6-M216 his3-D1 tpz1-L449A::hphMX poz1Δ::natMX6*
	5	TN14575	*tpz1-L449A poz1Δ*	*h$^+$ his3-D1 tpz1-L449A::hphMX poz1Δ::natMX6*
	6	TN14576	*tpz1-L449A poz1Δ*	*h$^-$ ade6-M216 his3-D1 tpz1-L449A::hphMX poz1Δ::natMX6*
	7	JH11577	*tpz1-L449R*	*h$^+$ ade6-M216 his3-D1 tpz1-L449R::hphMX*
	8	TN14444	*tpz1-L449R poz1Δ*	*h$^-$ his3-D1 tpz1-L449R::hphMX poz1Δ::natMX6*
	9	TN14445	*tpz1-L449R poz1Δ*	*h$^+$ ade6-M216 his3-D1 tpz1-L449R::hphMX poz1Δ::natMX6*
	10	TN14446	*tpz1-L449R poz1Δ*	*h$^+$ ade6-M216 his3-D1 tpz1-L449R::hphMX poz1Δ::natMX6*

Figure		Strain		Full Genotype[a]
S4	1	TN2411	*$tpz1^+$*	*h^- his3-D1*
	2,14,15	TN7196	*tpz1-myc*	*h^- his3-D1 $tpz1^+$::13myc-kanMX6*
	3-6	JH11181	*tpz1-L449R-myc*	*h^- ade6-M216 tpz1-L449R::13myc-kanMX6* (multiple 11x restreak single colonies derived from JH11181)
	7,16,23	LK8667	*ccq1Δ*	*h^- his3-D1 ccq1Δ::hphMX*
	8-13	JH11218	*tpz1-Y439R,L445R-myc*	*h^+ ade6-M210 his3-D1 tpz1-Y439R,L445R::13myc-kanMX6* (multiple 11x restreak single colonies derived from JH11218)
	17-22	JH11232	*tpz1-Y439R,L445R,L449R-myc*	*h^+ ade6-M210 his3-D1 tpz1-Y439R,L445R,L449R::13myc-kanMX6* (multiple 11x restreak single colonies derived from JH11232)
S5		CF248	*$trt1^+$/trt1Δ*	*h^+/h^- ade6-M210/ade6-M216 his3-D1/his3-D1 $trt1^+$/trt1Δ::$his3^+$*
		JH12706	*$trt1^+$/trt1Δ $ccq1^+$/ccq1Δ*	*h^+/h^- ade6-M210/ade6-M216 his3-D1/his3-D1 $trt1^+$/trt1Δ::$his3^+$ $ccq1^+$/ccq1Δ::hphMX*
		JH12263	*$trt1^+$/trt1Δ $tpz1^+$/tpz1-L449R*	*h^+/h^- ade6-M210/ade6-M216 his3-D1/his3-D1 $trt1^+$/trt1Δ::$his3^+$ $tpz1^+$/tpz1-L449R::hphMX*
S6A		CF199	*wt*	*h^- ade6-M210 his3-D1*
		TN6584	*ccq1Δ*	*h^- ade6-M210 his3-D1 ccq1Δ::hphMX*
		JH11578	*tpz1-L449R*	*h^- ade6-M216 his3-D1 tpz1-L449R::hphMX*
		JH11423	*tpz1-Y439R,L445R*	*h^- ade6-M210 his3-D1 tpz1-Y439R,L445R::hphMX*
		JH11574	*tpz1-Y439R,L445R,L449R*	*h^- ade6-M210 his3-D1 tpz1-Y439R,L445R,L449R::hphMX*
S6B	1,2	JH11722	*chk1-myc*	*h^- ade6-M216 his3-D1 $chk1^+$::9myc-2HA-6his-$ura4^+$ $tpz1^+$::hphMX*
	3	JH11724	*chk1-myc ccq1Δ*	*h^+ his3-D1 $chk1^+$::9myc-2HA-6his-$ura4^+$ $tpz1^+$::hphMX ccq1Δ::hphMX*
	4	JH11636	*chk1-myc tpz1-L449R*	*h^- ade6-M216 his3-D1 $chk1^+$::9myc-2HA-6his-$ura4^+$ tpz1-L449R::hphMX*
	5	JH11638	*chk1-myc tpz1-Y439R,L445R*	*h^- ade6-M216 his3-D1 $chk1^+$::9myc-2HA-6his-$ura4^+$ tpz1-Y439R,L445R::hphMX*
	6	JH11640	*chk1-myc tpz1-Y439R,L445R,L449R*	*h^+ ade6-M216 his3-D1 $chk1^+$::9myc-2HA-6his-$ura4^+$ tpz1-Y439R,L445R,L449R::hphMX*
S7A	1	TN2411	*his^-*	*h^- his3-D1*
	2	TN3784	*his^+*	*h^-*
	3	CF52	*telomere (1L)::$his3^+$*	*h^{90} ura4-DS/E ade6-M210 his3-D1 $his3^+$::tel(1L)*
	4	TN9133	*telomere (1L)::$his3^+$ ccq1Δ*	*h^- his3-D1 ccq1Δ::hphMX $his3^+$::tel(1L)*
	5	TN9630	*telomere (1L)::$his3^+$ tpz1-L449R*	*h^+ ade6-M216 his3-D1 tpz1-L449R::13myc-kanMX6 $his3^+$::tel(1L)*
	6	TN9623	*telomere (1L)::$his3^+$ tpz1-Y439R,L445R*	*h^- ade6-M210 his3-D1 tpz1-Y439R,L445R::13myc-kanMX6 $his3^+$::tel(1L)*
S7B	1	TN2411	*his^-*	*h^- his3-D1*
	2	TN3784	*his^+*	*h^-*
	3	CF52	*telomere (1L)::$his3^+$*	*h^{90} ura4-DS/E ade6-M210 his3-D1 $his3^+$::tel(1L)*
	4	TN9607	*telomere (1L)::$his3^+$ poz1Δ*	*h^+ ade6-M210 his3-D1 poz1Δ::natMX6 $his3^+$::tel(1L)*
	5	TN9614	*telomere (1L)::$his3^+$ tpz1-W498R,I501R*	*h^+ ade6-M210 his3-D1 tpz1-W498R,I501R::13myc-kanMX6 $his3^+$::tel(1L)*
	6	TN9618	*telomere (1L)::$his3^+$ tpz1-[1-485]*	*h^+ ade6-M210 his3-D1 tpz1-[1-485]::13myc-kanMX6 $his3^+$::tel(1L)*
S8A	1	TN6770	*ccq1-FLAG*	*h^+ his3-D1 $ccq1^+$::5FLAG-kanMX6*
	2	TN10988	*trt1Δ ccq1-FLAG*	*h^- ade6-M210 his3-D1 trt1Δ::$his3^+$ $ccq1^+$::5FLAG-kanMX6*
	3	TN10991	*trt1Δ ccq1-T93A-FLAG*	*h^- ade6-M210 his3-D1 trt1Δ::$his3^+$ ccq1-T93A::5FLAG-kanMX6*
	4	JH11174	*ccq1-FLAG tpz1-L449R-myc*	*h^- $ccq1^+$::5FLAG-kanMX6 tpz1-L449R::13myc-kanMX6*
	5	JH11238	*ccq1-FLAG tpz1-Y439R,L445R-myc*	*h^- his3-D1 $ccq1^+$::5FLAG-kanMX6 tpz1-Y439R,L445R::13myc-kanMX6*
	6	JH11248	*ccq1-FLAG tpz1-Y439R,L445R,L449R-myc*	*h^+ his3-D1 $ccq1^+$::5FLAG-kanMX6 tpz1-Y439R,L445R,L449R-13myc-kanMX6*
S8B	1	JH11289	*$ccq1^+$* (no tag)	*h^- his3-D1 $tpz1^+$::hphMX*
	2	JH11569	*ccq1-myc*	*h^+ his3-D1 $ccq1^+$::13myc-kanMX6 $tpz1^+$::hphMX*
	3	JH11622	*ccq1-myc tpz1-L449R*	*h^+ his3-D1 $ccq1^+$::13myc-kanMX6 tpz1-L449R::hphMX*
	4	JH11452	*ccq1-myc tpz1-Y439R,L445R*	*h^+ his3-D1 $ccq1^+$::13myc-kanMX6 tpz1-Y439R,L445R::hphMX*
	5	JH11609	*ccq1-myc tpz1-Y439R,L445R,L449R*	*h^+ his3-D1 $ccq1^+$::13myc-kanMX6 tpz1-Y439R,L445R,L449R::hphMX*

Figure		Strain		Full Genotype[a]
S8C	1	JH11289	$trt1^+$ (no tag)	h^- his3-D1 $tpz1^+$::hphMX
	2	JH11630	trt1-myc	h^- ade6-M216 his3-D1 $trt1^+$::G_8-13myc-kanMX6 $tpz1^+$::hphMX
	3	JH11626	trt1-myc tpz1-L449R	h^- ade6-M216 his3-D1 $trt1^+$::G_8-13myc-kanMX6 tpz1-L449R::hphMX
	4	JH11449	trt1-myc tpz1-Y439R,L445R	h^+ ade6-M210 his3-D1 $trt1^+$::G_8-13myc-kanMX6 tpz1-Y439R,L445R::hphMX
	5	JH11611	trt1-myc tpz1-Y439R,L445R,L449R	h^- ade6-M210 his3-D1 $trt1^+$::G_8-13myc-kanMX6 tpz1-Y439R,L445R,L449R::hphMX
	6	JH11644	trt1-myc ccq1Δ	h^+ his3-D1 $trt1^+$::G_8-13myc-kanMX6 $tpz1^+$::hphMX ccq1Δ::hphMX
S8D	1	TN2411	$tpz1^+$	h^- his3-D1
	2	TN7196	tpz1-myc	h^- his3-D1 $tpz1^+$::13myc-kanMX6
	3	TN9009	tpz1-myc ccq1Δ	h^+ his3-D1 tpz1-13myc:KanMX6 ccq1Δ::hphMX
	4	JH11181	tpz1-L449R-myc	h^- ade6-M216 tpz1-L449R::13myc-kanMX6
	5	JH11218	tpz1-Y439R,L445R-myc	h^+ ade6-M210 his3-D1 tpz1-Y439R,L445R::13myc-kanMX6
	6	JH11232	tpz1-Y439R,L445R,L449R-myc	h^+ ade6-M210 his3-D1 tpz1-Y439R,L445R,L449R::13myc-kanMX6
S9A	1	JH11289	$ccq1^+$ (no tag)	h^- his3-D1 $tpz1^+$::hphMX
	2	JH11569	ccq1-myc	h^+ his3-D1 $ccq1^+$::13myc-kanMX6 $tpz1^+$::hphMX
	3	TN14558	ccq1-myc tpz1-L449A	h^- his3-D1 $ccq1^+$::13myc-kanMX6 tpz1-L449A::hphMX
	4	JH11622	ccq1-myc tpz1-L449R	h^+ his3-D1 $ccq1^+$::13myc-kanMX6 tpz1-L449R::hphMX
S9B	1	JH11289	$trt1^+$ (no tag)	h^- his3-D1 $tpz1^+$::hphMX
	2	JH11630	trt1-myc	h^- ade6-M216 his3-D1 $trt1^+$::G_8-13myc-kanMX6 $tpz1^+$::hphMX
	3	JH11644	trt1-myc ccq1Δ	h^+ his3-D1 $trt1^+$::G_8-13myc-kanMX6 $tpz1^+$::hphMX ccq1Δ::hphMX
	4	TN14545	trt1-myc tpz1-L449A	h^- his3-D1 $trt1^+$::G_8-13myc-kanMX6 tpz1-L449A::hphMX
	5	JH11626	trt1-myc tpz1-L449R	h^- ade6-M216 his3-D1 $trt1^+$::G_8-13myc-kanMX6 tpz1-L449R::hphMX
	6	YTC12756	trt1-D743A-myc	h^- ade6-M210 his3-D1 trt1-D743A::G_8-13myc-kanMX6
S10B	1	TN2411	$tpz1^+$ (no tag)	h^- his3-D1
	2	TN7196	tpz1-myc	h^- his3-D1 $tpz1^+$::13myc-kanMX6
	3	JH10915	tpz1-[1-379]-myc	h^- ade6-M216 tpz1-[1-379]::13myc-kanMX6
	4	JH10919	tpz1-[1-420]-myc	h^- ade6-M216 his3-D1 tpz1-[1-420]::13myc-kanMX6
	5	JH11215	tpz1-[Δ421-485]-myc	h^+ ade6-M216 his3-D1 tpz1-[Δ421-485]::13myc-kanMX6
	6	JH10922	tpz1-[1-485]-myc	h^- ade6-M210 his3-D1 tpz1-[1-485]::13myc-kanMX6
S10C	1,7	TN2411	$tpz1^+$ (no tag)	h^- his3-D1
	2	TN7196	tpz1-myc	h^- his3-D1 $tpz1^+$::13myc-kanMX6
	3	JH10915	tpz1-[1-379]-myc	h^- ade6-M216 tpz1-[1-379]::13myc-kanMX6
	4	JH10919	tpz1-[1-420]-myc	h^- ade6-M216 his3-D1 tpz1-[1-420]::13myc-kanMX6
	5	JH11215	tpz1-[Δ421-485]-myc	h^+ ade6-M216 his3-D1 tpz1-[Δ421-485]::13myc-kanMX6
	6	JH10922	tpz1-[1-485]-myc	h^- ade6-M210 his3-D1 tpz1-[1-485]::13myc-kanMX6
S10D	1	TN7196	tpz1-myc	h^- his3-D1 $tpz1^+$::13myc-kanMX6
	2	JH10915	tpz1-[1-379]-myc	h^- ade6-M216 tpz1-[1-379]::13myc-kanMX6
	3	JH10919	tpz1-[1-420]-myc	h^- ade6-M216 his3-D1 tpz1-[1-420]::13myc-kanMX6
	4	JH11215	tpz1-[Δ421-485]-myc	h^+ ade6-M216 his3-D1 tpz1-[Δ421-485]::13myc-kanMX6
	5	JH10922	tpz1-[1-485]-myc	h^- ade6-M210 his3-D1 tpz1-[1-485]::13myc-kanMX6
S11B		TN7196	tpz1-myc	h^- his3-D1 $tpz1^+$::13myc-kanMX6
		JH12981	tpz1-myc pot1Δ	h^- ade6-M216 his3-D1 $tpz1^+$::13myc-kanMX6 pot1Δ::natMX
S11C	1	TN7196	tpz1-myc	h^- his3-D1 $tpz1^+$::13myc-kanMX6
	2	YTC9366	tpz1-myc poz1Δ	h^- his3-D1 $tpz1^+$::13myc-kanMX6 poz1Δ::natMX6
	3	TN9009	tpz1-myc ccq1Δ	h^+ his3-D1 tpz1-13myc:KanMX6 ccq1Δ::hphMX
	4	JH11701	tpz1-myc poz1Δ ccq1Δ	h^+ his3-D1 $tpz1^+$::13myc-kanMX6 poz1Δ::natMX6 ccq1Δ::hphMX
	5	JH11835	tpz1-L449R-myc	h^+ ade6-M210 his3-D1 tpz1-L449R::13myc-kanMX6
	6	JH11190	tpz1-W498R,I501R-myc	h^+ leu1-32 ura4-D18 ade6-M216 his3-D1 tpz1-W498R,I501R::13myc-kanMX6
	7	JH11781	tpz1-L449R,W498R,I501R-myc	h^+ ade6-M216 his3-D1 tpz1-L449R,W498R,I501R::13myc-kanMX6
	8	JH10922	tpz1-[1-485]-myc	h^- ade6-M210 his3-D1 tpz1-[1-485]::13myc-kanMX6
	9	JH11790	tpz1-[1-485]-L449R-myc	h^- ade6-M216 his3-D1 tpz1-[1-485]-L449R::13myc-kanMX6

Figure		Strain		Full Genotype[a]
S11D	1	YTC9716	pot1-FLAG	h^+ his3-D1 $pot1^+$::3FLAG-kanMX
	2	JH11046	pot1-FLAG tpz1-myc	h^- his3-D1 $pot1^+$::3FLAG-kanMX $tpz1^+$::13myc-kanMX6
	3	JH12971	pot1-FLAG tpz1-myc ccq1Δ	h^- his3-D1 $pot1^+$::3FLAG-kanMX $tpz1^+$::13myc-kanMX6 ccq1Δ::hphMX
	4	JH12975	pot1-FLAG tpz1-myc poz1Δ	h^+ his3-D1 $pot1^+$::3FLAG-kanMX $tpz1^+$::13myc-kanMX6 poz1Δ::natMX6
	5	JH12977	pot1-FLAG tpz1-myc poz1Δ ccq1Δ	h^+ his3-D1 $pot1^+$::3FLAG-kanMX $tpz1^+$::13myc-kanMX6 poz1Δ::natMX6 ccq1Δ::hphMX
S12A	1	TN2411	$tpz1^+$	h^- his3-D1
	2	YTC8354	poz1Δ	h^- ade6-M216 his3-D1 poz1Δ::natMX6
	3	TN5345	rap1Δ	h^+ his3-D1 rap1Δ::$ura4^+$
	4	TN7196	tpz1-myc	h^- his3-D1 $tpz1^+$::13myc-kanMX6
	5	YTC9366	tpz1-myc poz1Δ	h^- his3-D1 $tpz1^+$::13myc-kanMX6 poz1Δ::natMX6
	6	YTC9310	tpz1-myc rap1Δ	h^- his3-D1 $tpz1^+$::13myc-kanMX6 rap1Δ::$ura4^+$
	7	JH12951	tpz1-myc poz1Δ rap1Δ	h^- his3-D1 $tpz1^+$::13myc-kanMX6 poz1Δ::natMX6 rap1Δ::$ura4^+$
	8	JH11191	tpz1-W498R,I501R-myc	h^+ ade6-M216 his3-D1 tpz1-W498R,I501R::13myc-kanMX6
	9	JH11692	tpz1-W498R,I501R-myc poz1Δ	h^- ade6-M216 his3-D1 tpz1-W498R,I501R::13myc-kanMX6 poz1Δ::natMX6
	10	JH12052	tpz1-W498R,I501R-myc rap1Δ	h^+ ade6-M216 his3-D1 tpz1-W498R,I501R::13myc-kanMX6 rap1Δ::$ura4^+$
	11	JH12955	tpz1-W498R,I501R-myc poz1Δ rap1Δ	h^- his3-D1 tpz1-W498R,I501R::13myc-kanMX6 poz1Δ::natMX6 rap1Δ::$ura4^+$
S12B	1	TN2411	$tpz1^+$	h^- his3-D1
	2	JH12908	tpz1-W498R,I501R	h^- ade6-M210 his3-D1 tpz1-W498R,I501R::hphMX
	3	TN7217	ccq1-myc	h^- his3-D1 $ccq1^+$::13myc-kanMX6
	4	YTC8712	ccq1-myc poz1Δ	h^+ his3-D1 $ccq1^+$::13myc-kanMX6 poz1Δ::natMX6
	5	JH12894	ccq1-myc tpz1-W498R,I501R	h^- his3-D1 $ccq1^+$::13myc-kanMX6 tpz1-W498R,I501R::hphMX
	6	JH12897	ccq1-myc tpz1-W498R,I501R poz1Δ	h^- his3-D1 $ccq1^+$::13myc-kanMX6 tpz1-W498R,I501R::hphMX poz1Δ::natMX6
S12C	1	TN11289	$tpz1^+$	h^- his3-D1 $tpz1^+$::hphMX
	2	JH12908	tpz1-W498R,I501R	h^- ade6-M210 his3-D1 tpz1-W498R,I501R::hphMX
	3	JH12265	poz1-myc	h^+ his3-D1 $poz1^+$::13myc-kanMX6 $tpz1^+$::hphMX
	4	JH12267	poz1-myc rap1Δ	h^+ his3-D1 $poz1^+$::13myc-kanMX6 $tpz1^+$::hphMX rap1Δ::$ura4^+$
	5	JH12659	poz1-myc tpz1-W498R,I501R	h^+ ade6-M210 his3-D1 $poz1^+$::13myc-kanMX6 tpz1-W498R,I501R::hphMX
	6	JH12056	poz1-myc tpz1-W498R,I501R rap1Δ	h^+ ade6-M210 his3-D1 $poz1^+$::13myc-kanMX6 tpz1-W498R,I501R::hphMX rap1Δ::$ura4^+$
S12D	1	TN11289	$tpz1^+$	h^- his3-D1 $tpz1^+$::hphMX
	2	JH12908	tpz1-W498R,I501R	h^- ade6-M210 his3-D1 tpz1-W498R,I501R::hphMX
	3	JH11630	trt1-myc	h^- ade6-M216 his3-D1 $trt1^+$::G_8-13myc-kanMX6 $tpz1^+$::hphMX
	4	JH12770	trt1-myc poz1Δ	h^- ade6-M216 his3-D1 $trt1^+$::G_8-13myc-kanMX6 $tpz1^+$::hphMX poz1Δ::natMX6
	5	JH12766	trt1-myc tpz1-W498R,I501R	h^- his3-D1 $trt1^+$::G_8-13myc-kanMX6 tpz1-W498R,I501R::hphMX
S13A	1	TN2411	$tpz1^+$	h^- his3-D1
	2	YTC8354	poz1Δ	h^- ade6-M216 his3-D1 poz1Δ::natMX6
	3	TN7196	tpz1-myc	h^- his3-D1 $tpz1^+$::13myc-kanMX6
	4	YTC9366	tpz1-myc poz1Δ	h^- his3-D1 $tpz1^+$::13myc-kanMX6 poz1Δ::natMX6
	5	JH11191	tpz1-W498R,I501R-myc	h^+ ade6-M216 his3-D1 tpz1-W498R,501R::13myc-kanMX6
	6	JH11692	tpz1-W498R,I501R-myc poz1Δ	h^- ade6-M216 his3-D1 tpz1-W498R,I501R::13myc-kanMX6 poz1Δ::natMX6
S13B	1	TN2411	$tpz1^+$	h^- his3-D1
	2	JH12908	tpz1-W498R,I501R	h^- ade6-M210 his3-D1 tpz1-W498R,I501R::hphMX
	3	TN7217	ccq1-myc	h^- his3-D1 $ccq1^+$::13myc-kanMX6
	4	YTC8712	ccq1-myc poz1Δ	h^+ his3-D1 $ccq1^+$::13myc-kanMX6 poz1Δ::natMX6
	5	JH12895	ccq1-myc tpz1-W498R,I501R	h^+ his3-D1 $ccq1^+$::13myc-kanMX6 tpz1-W498R,I501R::hphMX
	6	JH12897	ccq1-myc tpz1-W498R,I501R poz1Δ	h^- his3-D1 $ccq1^+$::13myc-kanMX6 tpz1-W498R,I501R::hphMX poz1Δ::natMX6

Figure		Strain		Full Genotype[a]
S13C	1	TN11289	$tpz1^+$	h^- his3-D1 $tpz1^+$::hphMX
	2	JH12908	tpz1-W498R,I501R	h^- ade6-M210 his3-D1 tpz1-W498R,I501R::hphMX
	3	JH12265	poz1-myc	h^+ his3-D1 $poz1^+$::13myc-kanMX6 $tpz1^+$::hphMX
	4	JH12267	poz1-myc rap1Δ	h^+ his3-D1 $poz1^+$::13myc-kanMX6 $tpz1^+$::hphMX rap1Δ::$ura4^+$
	5	JH12659	poz1-myc tpz1-W498R,I501R	h^+ ade6-M210 his3-D1 $poz1^+$::13myc-kanMX6 tpz1-W498R,I501R::hphMX
	6	JH12056	poz1-myc tpz1-W498R,I501R rap1Δ	h^+ ade6-M210 his3-D1 $poz1^+$::13myc-kanMX6 tpz1-W498R,I501R::hphMX rap1Δ::$ura4^+$
S13D	1	TN11289	$tpz1^+$	h^- his3-D1 $tpz1^+$::hphMX
	2	JH12908	tpz1-W498R,I501R	h^- ade6-M210 his3-D1 tpz1-W498R,I501R::hphMX
	3	JH11630	trt1-myc	h^- ade6-M216 his3-D1 $trt1^+$::G_8-13myc-kanMX6 $tpz1^+$::hphMX
	4	JH12770	trt1-myc poz1Δ	h^- ade6-M216 his3-D1 $trt1^+$::G_8-13myc-kanMX6 $tpz1^+$::hphMX poz1Δ::natMX6
	5	JH12766	trt1-myc tpz1-W498R,I501R	h^- his3-D1 $trt1^+$::G_8-13myc-kanMX6 tpz1-W498R,I501R::hphMX

[1]All strains are *leu1-32 ura4-D18* (haploid) or *leu1-32/leu1-32 ura4-D18/ura4-D18* (diploid)

Table S3 Plasmids used to integrate *tpz1* mutant alleles into fission yeast.

Plasmid (Lab stock #)	Mutant alleles
pBS-tpz1-13myc-kanMX (607)	*tpz1-myc*
pBS-tpz1-L449R-13myc-kanMX (697)	*tpz1-L449R-myc*
pBS-tpz1-L449A-13myc-kanMX (836)	*tpz1-L449A-myc*
pBS-tpz1-Y439R,L445R-13myc-kanMX (716)	*tpz1-Y439R,L445R-myc*
pBS-tpz1-Y439R,L445R,L449R-13myc-kanMX (718)	*tpz1-Y439R,L445R,L449R-myc*
pBS-tpz1(1-485)-13myc-kanMX (654)	*tpz1-[1-485]-myc*
pBS-tpz1-W498R,I501R-13myc-kanMX (707)	*tpz1-W498R,I501R-myc*
pBS-tpz1(1-485)-L449R-13myc-kanMX (773)	*tpz1-[1-485]-L449R-myc*
pBS-tpz1-L449R,W498R,I501R-13myc-kanMX (772)	*tpz1-L449R,W498R,I501R-myc*
pBS-tpz1(1-379)-13myc-kanMX (652)	*tpz1-[1-379]-myc*
pBS-tpz1(1-420)-13myc-kanMX (653)	*tpz1-[1-420]-myc*
pBS-tpz1(Δ421-485)-13myc-kanMX (670)	*tpz1-[Δ421-485]-myc*

Table S4 Plasmids used in yeast 2-hybrid assays.

Plasmid (Lab stock #)	Description in Figures
pGADT7 (352)	vector
pGADT7-Poz1 (452)	Poz1
pGAD-GH-Ccq1 (549)	Ccq1
pTM623 (pMP623; Pot1 with GAD) (464)[1]	Pot1
pGBKT7 (356)	vector
pGBKT7-Tpz1 (495)	Tpz1-FL (full length)
pGBKT7-Tpz1-[379-508] (614)	Tpz1-[379-508]
pGBKT7-Tpz1-[421-508] (616)	Tpz1-[421-508]
pGBKT7-Tpz1-[421-485] (651)	Tpz1-[421-485]
pGBKT7-Tpz1-[486-508] (650)	Tpz1-[486-508]
pGBKT7-Tpz1-[1-485] (708)	Tpz1-[1-485]
pGBKT7-Tpz1-[Δ421-485] (689)	Tpz1-[Δ421-485]
pGBKT7-Tpz1-Y439R (682)	Y439R
pGBKT7-Tpz1-L445R (683)	L445R
pGBKT7-Tpz1-E446R (684)	E446R
pGBKT7-Tpz1-Y439R,L445R,E446R (666)	Y439R,L445R,E446R
pGBKT7-Tpz1-Y439R,L445R (700)	Y439R,L445R
pGBKT7-Tpz1-L449R,Y453R (667)	L449R,Y453R
pGBKT7-Tpz1-L449R (685)	L449R
pGBKT7-Tpz1-Y439R,L445R,L449R (701)	Y439R,L445R,L449R
pGBKT7-Tpz1-Y453R (686)	Y453R
pGBKT7-Tpz1-[421-485]-L449R (763)	Tpz1-[421-485] L449R
pGBKT7-Tpz1-[421-485]-Y439R,L445R (762)	Tpz1-[421-485] Y439R,L445R
pGBKT7-Tpz1-[421-485]-Y439R,L445R,L449R (764)	Tpz1-[421-485] Y439R,L445R,L449R
pGBKT7-Tpz1-W498R,I501R (669)	W498R,I501R
pGBKT7-Tpz1-[486-508]-W498R,I501R (673)	Tpz1-[486-508] W498R,I501R
pGBKT7-Tpz1-K386A (594)	K386A
pGBKT7-Tpz1-R433A,K434A (597)	R433A,K434A
pGBKT7-Tpz1-E455A,K459A,K460A (598)	E455A,K459A,K460A
pGBKT7-Tpz1-D497A,K500A,R505A (600)	D497A,K500A,R505A
pGBKT7-Tpz1-R433A,K434A,E455A,K459A,K460A (617)	R433A,K434A,E455A,K459A,K460A
pGBKT7-Tpz1-R433A,K434A,D497A,K500A,R505A (619)	R433A,K434A,D497A,K500A,R505A
pGBKT7-Tpz1-E455A,K459A,K460A,D497A,K500A,R505A (620)	E455A,K459A,K460A,D497A,K500A,R505A
pGBKT7-Tpz1-R433E,K434E (643)	R433E,K434E
pGBKT7-Tpz1-K459E,K460E (644)	K459E,K460E
pGBKT7-Tpz1-K500E,R505E (645)	K500E,R505E
pGBKT7-Tpz1-W498A,I501A (794)	W498A,I501A
pGBKT7-Tpz1-L449A (795)	L449A
pGBKT7-Tpz1-Y439A,L445A (796)	Y439A,L445A

[1]From Ishikawa lab.

Table S5 Statistical analysis of ChIP and *TER1* co-IP data by 2-tailed Student's t-test.

Figure		Genotypes and p-values[1]				
5A		no tag	*tpz1-myc*	*tpz1-myc ccq1Δ*	*tpz1-L449R -myc*	*tpz1-Y439R, L445R-myc*
	tpz1-myc	2.5x10^{-4}				
	tpz1-myc ccq1Δ	4.9x10^{-5}	0.024			
	tpz1-L449R-myc	1.9x10^{-4}	9.3x10^{-3}	0.22		
	tpz1-Y439R,L445R-myc	8.9x10^{-3}	7.6x10^{-3}	0.11	0.41	
	tpz1-Y439R,L445R,L449R-myc	0.018	0.029	0.46	0.98	0.61
5B		no tag	*ccq1-myc*	*ccq1-myc tpz1-L449R*	*ccq1-myc tpz1-Y439R, L445R*	
	ccq1-myc	4.6x10^{-7}				
	ccq1-myc tpz1-L449R	5.6x10^{-6}	1.5x10^{-4}			
	ccq1-myc tpz1-Y439R,L445R	2.3x10^{-5}	7.3x10^{-5}	0.037		
	ccq1-myc tpz1-Y439R,L445R,L449R	2.1x10^{-5}	2.0x10^{-4}	0.29	0.21	
5C		no tag	*trt1-myc*	*trt1-myc ccq1Δ*	*trt1-myc tpz1-L449R*	*trt1-myc tpz1-Y439R, L445R*
	trt1-myc	4.9x10^{-6}				
	trt1-myc ccq1Δ	8.2x10^{-3}	4.1x10^{-3}			
	trt1-myc tpz1-L449R	1.7x10^{-5}	3.2x10^{-4}	0.47		
	trt1-myc tpz1-Y439R,L445R	2.7x10^{-3}	5.1x10^{-3}	0.82	0.67	
	trt1-myc tpz1-Y439R,L445R,L449R	8.3x10^{-3}	4.4x10^{-3}	0.96	0.53	0.87
7A		*tpz1-myc*	*tpz1-myc poz1Δ*	*tpz1-W498R, I501R-myc*		
	tpz1-myc poz1Δ	2.2x10^{-3}				
	tpz1-W498R,I501R-myc	1.1x10^{-3}	0.65			
	tpz1-W498R,I501R-myc poz1Δ	7.6x10^{-3}	0.55	0.41		
7B		*ccq1-myc*	*ccq1-myc poz1Δ*	*ccq1-myc tpz1-498R, I501R*		
	ccq1-myc poz1Δ	0.39				
	ccq1-myc tpz1-W498R,I501R	0.10	0.77			
	ccq1-myc tpz1-W498R,I501R poz1Δ	0.07	0.28	0.35		
7C		*poz1-myc*	*poz1-myc rap1Δ*	*poz1-myc tpz1-498R, I501R*		
	poz1-myc rap1Δ	7.7x10^{-3}				
	poz1-myc tpz1-W498R,I501R	3.9x10^{-3}	1.6x10^{-3}			
	poz1-myc tpz1-W498R,I501R rap1Δ	1.8x10^{-3}	4.3x10^{-4}	0.21		

7D		*trt1-myc*	*trt1-myc poz1Δ*			
	trt1-myc poz1Δ	$5.3x10^{-6}$				
	trt1-myc tpz1-W498R,I501R	$6.0x10^{-5}$	0.09			

S8D		no tag	*tpz1-myc*	*tpz1-myc ccq1Δ*	*tpz1-L449R -myc*	*tpz1-Y439R, L445R-myc*
	tpz1-myc	$1.1x10^{-3}$				
	tpz1-myc ccq1Δ	0.46	$2.0x10^{-4}$			
	tpz1-L449R-myc	0.80	$1.3x10^{-4}$	0.19		
	tpz1-Y439R,L445R-myc	0.79	$4.8x10^{-4}$	0.50	0.52	
	tpz1-Y439R,L445R,L449R-myc	0.54	$2.0x10^{-4}$	0.22	0.24	0.64

S9A		no tag	*ccq1-myc*	*ccq1-myc tpz1-L449A*		
	ccq1-myc	$2.7x10^{-3}$				
	ccq1-myc tpz1-L449A	$8.2x10^{-3}$	0.056			
	ccq1-myc tpz1-L449R	$6.4x10^{-6}$	0.035	0.66		

S9B		no tag	*trt1-myc*	*trt1-myc ccq1Δ*	*trt1-myc tpz1-L449A*	*rt1-myc tpz1-L449R*
	trt1-myc	$4.9x10^{-6}$				
	trt1-myc ccq1Δ	$8.2x10^{-3}$	$4.1x10^{-3}$			
	trg1-myc tpz1-L449A	$1.1x10^{-5}$	$1.2x10^{-2}$	0.15		
	trt1-myc tpz1-L449R	$1.7x10^{-5}$	$3.2x10^{-4}$	0.47	0.20	
	trt1-D743A-myc	$8.1x10^{-9}$	$6.9x10^{-3}$	$7.3x10^{-5}$	$4.8x10^{-5}$	$1.1x10^{-7}$

S13A		no tag (*tpz1*$^{+}$)	*poz1Δ* (no tag)	*tpz1-myc*	*tpz1-myc poz1Δ*	*tpz1-W498R, I501R-myc*
	poz1Δ (no tag)	0.54				
	tpz1-myc	$1.8x10^{-9}$	$2.8x10^{-5}$			
	tpz1-myc poz1Δ	$5.2x10^{-9}$	$3.1x10^{-5}$	$1.0x10^{-3}$		
	tpz1-W498R,I501R-myc	$7.9x10^{-10}$	$5.1x10^{-6}$	$2.5x10^{-3}$	0.67	
	tpz1-W498R,I501R-myc poz1Δ	$1.9x10^{-7}$	$3.2x10^{-4}$	$2.6x10^{-3}$	0.66	0.50

S13B		no tag (*tpz1*$^{+}$)	*tpz1-W498R, I501R* (no tag)	*ccq1-myc*	*ccq1-myc poz1Δ*	*ccq1-myc tpz1-498R, I501R*
	tpz1-W498R,I501R (no tag)	$7.2x10^{-2}$				
	ccq1-myc	$1.1x10^{-4}$	$1.1x10^{-4}$			
	ccq1-myc poz1Δ	$2.1x10^{-4}$	$2.1x10^{-4}$	0.037		
	ccq1-myc tpz1-W498R,I501R	$1.0x10^{-4}$	$1.0x10^{-4}$	0.041	0.85	
	ccq1-myc tpz1-W498R,I501R poz1Δ	$1.0x10^{-3}$	$1.0x10^{-3}$	0.31	0.28	0.32

S13C		no tag (*tpz1*$^+$)	*tpz1-W498R, I501R* (no tag)	*poz1-myc*	*poz1-myc rap1Δ*	*poz1-myc tpz1-498R, I501R*
	tpz1-W498R,I501R (no tag)	0.44				
	poz1-myc	$1.7x10^{-3}$	$7.6x10^{-5}$			
	poz1-myc rap1Δ	$1.2x10^{-3}$	$4.3x10^{-5}$	0.048		
	poz1-myc tpz1-W498R,I501R	0.010	$6.2x10^{-4}$	$2.1x10^{-3}$	$1.6x10^{-3}$	
	poz1-myc tpz1-W498R,I501R rap1Δ	$4.5x10^{-3}$	$2.6x10^{-4}$	$5.0x10^{-4}$	$4.1x10^{-4}$	0.27

S13D		no tag (*tpz1*$^+$)	*tpz1-W498R, I501R* (no tag)	*trt1-myc*	*trt1-myc poz1Δ*	
	tpz1-W498R,I501R (no tag)	0.97				
	trt1-myc	$9.8x10^{-6}$	$3.6x10^{-6}$			
	trt1-myc poz1Δ	$2.9x10^{-6}$	$1.7x10^{-6}$	$1.1x10^{-3}$		
	trt1-myc tpz1-W498R,I501R	$3.3x10^{-5}$	$2.6x10^{-5}$	0.011	0.61	

[1]Statistically significant differences (p≤0.05) are highlighted with red letters.
